# Transposase expression, element abundance, element size, and DNA repair determine the mobility and heritability of *PIF*/*Pong*/*Harbinger* transposable elements

**DOI:** 10.3389/fcell.2023.1184046

**Published:** 2023-06-09

**Authors:** Priscilla S. Redd, Lisette Payero, David M. Gilbert, Clinton A. Page, Reese King, Edward V. McAssey, Dalton Bodie, Stephanie Diaz, C. Nathan Hancock

**Affiliations:** ^1^ Department of Biology and Geology, University of South Carolina Aiken, Aiken, SC, United States; ^2^ Department of Molecular Biology and Genetics, Cornell University, Ithaca, NY, United States; ^3^ Department of Crop and Soil Science, Institute of Plant Breeding, Genetics, and Genomics, University of Georgia, Athens, GA, United States

**Keywords:** transposable elements, excision, insertion, replication, rDNA

## Abstract

**Introduction:** Class II DNA transposable elements account for significant portions of eukaryotic genomes and contribute to genome evolution through their mobilization. To escape inactivating mutations and persist in the host genome over evolutionary time, these elements must be mobilized enough to result in additional copies. These elements utilize a “cut and paste” transposition mechanism that does not intrinsically include replication. However, elements such as the rice derived *mPing* element have been observed to increase in copy number over time.

**Methods:** We used yeast transposition assays to test several parameters that could affect the excision and insertion of *mPing* and its related elements. This included development of novel strategies for measuring element insertion and sequencing insertion sites.

**Results:** Increased transposase protein expression increased the mobilization frequency of a small (430 bp) element, while overexpression inhibition was observed for a larger (7,126 bp) element. Smaller element size increased both the frequency of excision and insertion of these elements. The effect of yeast ploidy on element excision, insertion, and copy number provided evidence that homology dependent repair allows for replicative transposition. These elements were found to preferentially insert into yeast rDNA repeat sequences.

**Discussion:** Identifying the parameters that influence transposition of these elements will facilitate their use for gene discovery and genome editing. These insights in to the behavior of these elements also provide important clues into how class II transposable elements have shaped eukaryotic genomes.

## 1 Introduction

Transposable elements (TEs) are mobile segments of DNA that can create new copies of themselves in the genome. When inserted within or near genes, TEs can disrupt gene expression, resulting in phenotypic changes. These mutations, coupled with the fact that TEs make up large portions of genomes, make these elements powerful drivers of genome evolution (Oliver et al., 2013). The observation that only recently replicated TEs are functional (Vitte and Bennetzen, 2006) indicates that movement and replication is crucial to TE survival on an evolutionary time scale. Thus, we must have a clear understanding of the mechanisms controlling TE mobility if we are to understand genome evolution.

Different classes of TEs rely on different transposition mechanisms. Class I TEs (retrotransposons) have a well described mechanism for replication where reverse transcriptase synthesizes a new copy of the element from an RNA template. In contrast, the transposition mechanism for eukaryotic class II (DNA) elements has been classified as “copy-in,” “copy-out/paste-in,” or “cut-out/paste-in” (Curcio and Derbyshire, 2003). Copy-in elements, such as *Mu* and *Tn3*, are hypothesized to only cleave and transfer a single strand of DNA and replication of the resulting “Shapiro” intermediate produces a second copy of the element (Shapiro, 1979). Copy-out/paste-in (*IS3* family) elements form a circular plasmid like structure, which is replicated by the host before reinsertion into the genome (Sekine et al., 1999). Evidence for the Shapiro intermediates or circular elements only exists for the *Maverick* and *Helitron* super families in eukaryotes, respectively (Kapitonov and Jurka, 2001; Pritham et al., 2007). The majority of eukaryotic DNA TEs rely on the cut-out/paste-in mechanism, in which elements are excised from their genomic location by the activity of transposase proteins, then reinserted elsewhere in the genome (Yuan and Wessler, 2011; Craig, 2020). This “cut and paste” mechanism utilized by the majority of eukaryotic DNA TEs lacks an intrinsic replication mechanism and observations of element replication are rare. The finding that the *Ac/Ds* element transposes immediately after DNA replication, when the DNA is hemimethylated, suggests that the timing during the cell cycle may be an important factor (Ros and Kunze, 2001). However, relatively little is known about how the host genetic machinery is exploited to produce new copies of many elements.

The focus of this study is the *PIF/Harbinger* superfamily of TEs, which are found in almost all eukaryotes (Zhang et al., 2004; Han et al., 2015). These class II elements utilize the cut-out/paste-in mechanism catalyzed by two separate proteins referred to as ORF1 and Transposase [TPase] (Yang et al., 2007; Sinzelle et al., 2008; Johnson et al., 2021). The ORF1 protein contains a DNA binding domain that is hypothesized to bind to the terminal inverted repeat (TIR) sequences that delineate the ends of the elements (Sinzelle et al., 2008; Hancock et al., 2010). The TPase protein has a catalytic DDE domain for DNA cleavage (Yuan and Wessler, 2011) and interacts with ORF1 to form the functional transposition complex (Sinzelle et al., 2008; Velanis et al., 2020). The best studied *PIF*/*Pong*/*Harbinger* superfamily elements are the *mPing*, *Ping*, and *Pong* elements naturally found in the rice genome (Jiang et al., 2003; Kikuchi et al., 2003; Nakazaki et al., 2003). *mPing* is a Miniature Inverted-repeat Transposable Element (MITE) derived from the larger *Ping* element and is highly active in some rice cultivars (Naito et al., 2006; Naito et al., 2009). The *mPing* element was shown to increase in copy number during tissue culture treatment (Jiang et al., 2003), and naturally reached hundreds of genomic copies in some rice cultivars (Naito et al., 2006; Naito et al., 2009; Yasuda et al., 2013; Chen et al., 2019). As a non-autonomous element, *mPing* does not encode ORF1 and TPase proteins, but instead is mobilized by the ORF1 and TPase proteins encoded by the autonomous *Ping* or *Pong* elements (Yang et al., 2007; Hancock et al., 2010).

**FIGURE 1:**
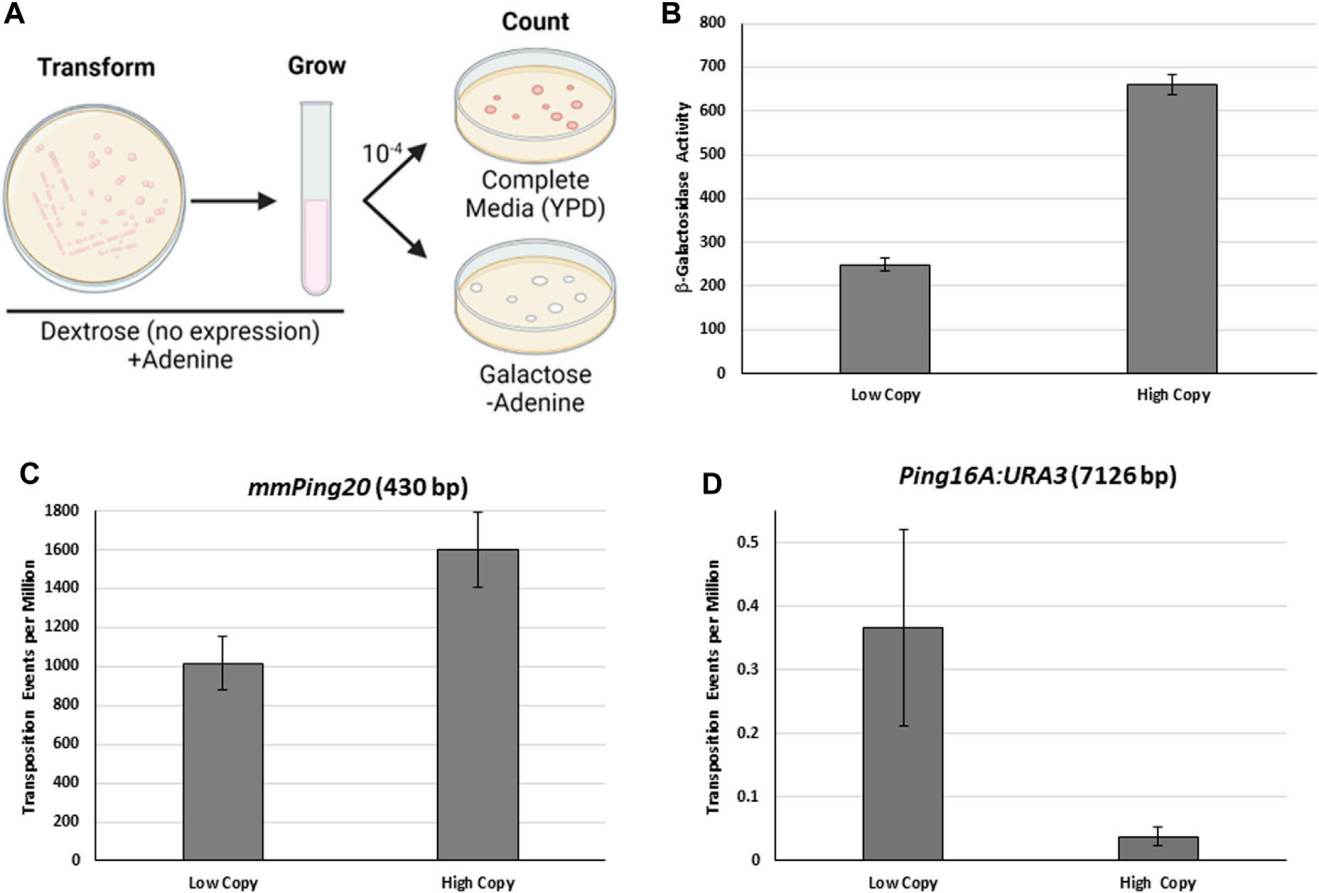
The effect of protein expression on *mPing* excision frequency. **(A)** Diagram of the yeast transposition assay used in this study. The number of colonies on the YPD and galactose plates is used to calculate the transposition frequency. The *ADE2* revertant colonies on the galactose plates can be analyzed further to determine insertion frequency and location. **(B)** β-galactosidase assay comparing expression of *LacZ* from a low copy plasmid (pAG 413 GAL) and a high copy plasmid (pAG 423 GAL) in *S. cerevisiae* grown in 2% Galactose. The *LacZ* reporter provides an approximation for expression of ORF1 and TPase in **(C,D)**. Error bars represent the standard error of three replicates. C&D: Yeast transposition assays using a genomic *mmPing20:ADE2*. **(C)** or *Ping16A:URA3:ADE2*. **(D)** reporter with expression of *TPase:T2A:ORF1* from the same low and high copy plasmids. Error bars represent the standard error of six replicates.

The activity of the *mPing* element can be studied using an established yeast transposition assay, in which *mPing* is inserted into the coding region of *ADE2* to track the excision of *mPing* and subsequent repair of the excision site (Hancock et al., 2010). Expression of ORF1 and TPase proteins is induced by the presence of galactose and yeast that experience excision of *mPing* and precise repair of the excision site result in a colony on plates lacking adenine (Figure 1A). This assay has allowed for the discovery of several factors that regulate transposition of *mPing* and a synthetic *mPong* element. First, it was shown that increasing nuclear localization of the ORF1 and TPase proteins by strengthening a nuclear localization signal and deleting a nuclear export signal respectively results in a significant increase in element mobility (Hancock et al., 2010; Payero et al., 2016). Alteration of the TIR sequences away from the consensus sequence decreases the excision frequency (Chen et al., 2019; Redd et al., 2023). In addition, internal sequences are required for efficient transposition, and alteration can increase or decrease mobility (Redd et al., 2023). Yeast-based experiments also showed that repair of the *ADE2* allele at *mPing* excision sites in haploid yeast is primarily a result of non-homologous end joining [NHEJ] (Gilbert et al., 2015). The goal of this study was to continue to describe factors that determine the mobility and retention of *PIF/Pong/Harbinger* elements in the genome.

## 2 Results

### 2.1 Altering transposase expression affects excision frequency

Many DNA TEs exhibit a phenomenon called overexpression inhibition, where an excess of transposase proteins results in reduced transposition (Heinlem et al., 1994; Geurts et al., 2003). Although previous experiments have shown that expression of ORF1 and TPase is required for *mPing* transposition, it was not known how expression level effected transposition frequency. To test the effect of protein expression, we used a previously developed genomic *ADE2* reporter containing the hyperactive version of *mPing* called *mmPing20* (Johnson et al., 2021). We altered the protein expression levels using low copy (pAG 413 GAL) and high copy (pAG 423 GAL) plasmids which are present at about 3 and 20 copies respectively (Karim et al., 2013). Figure 1B shows β-galactosidase assays for identical *LacZ* genes cloned into these plasmids. The high copy plasmid produces significantly (*t*-test, *p* < 0.001) higher levels of β-galactosidase protein than the low copy plasmid. When hyperactive *Pong* TPase and ORF1 proteins (Payero et al., 2016) fused by a T2A peptide (Kim et al., 2011) were expressed in these plasmids, we observed significantly higher (*t*-test, *p* < 0.05) excision of the 430 bp *mmPing20* element by the high copy expression plasmid (Figure 1C). This result is consistent with increased protein concentration increasing the likelihood of forming an active transposition complex. In contrast, performing the same experiments with the much larger 7,126 bp *Ping16A:Ura3* element produced the opposite effect (Figure 1D), with lower expression producing more transposition (*t*-test, *p* = 0.06). This suggests that overexpression inhibition is occurring for larger elements where the TIRs are farther apart.

### 2.2 Increased element copy number increases excision frequency

**FIGURE 2:**
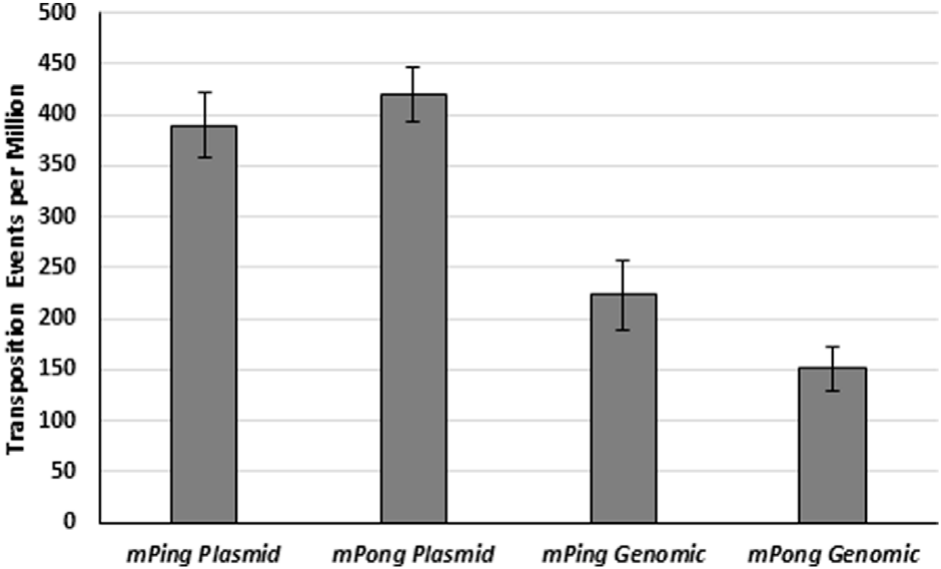
Transposition from plasmid and genome. Yeast transposition assay of *mPing* and *mPong* using pWL89a plasmid or genomic *ADE2* reporters. A low copy plasmid (pAG 413 GAL) was used to express *ORF1:T2A:TPase*. Error bars represent the standard error of five to six replicates.

In addition to differences in the amount of protein expression, significant differences in *mPing* element copy number are found in different rice cultivars (Chen et al., 2019). We predicted that increased element abundance in the yeast genome would increase the probability of transposition complex formation. To test the effect of the element’s copy number, we compared the excision frequencies for *mPing* and *mPong* elements present as a single genomic copy to excision from multiple copies [2-5 copies per cell (Karim et al., 2013)] present on a low copy plasmid (Figure 2). We observed significantly more *ADE2* revertant colonies from the plasmid encoded elements than the genomic copies (*t*-test, *mPing p* < 0.01, *mPong p* < 0.001), indicating that more excision and subsequent *ADE2* repair occurred. This result supports a model in which excision frequency is correlated with the rate at which the ORF1 and TPase proteins form a complex with the element. Increasing the copy number appears to effectively increases its probability of interacting with the ORF1 and TPase proteins.

### 2.3 Smaller elements excise more frequently

**FIGURE 3:**
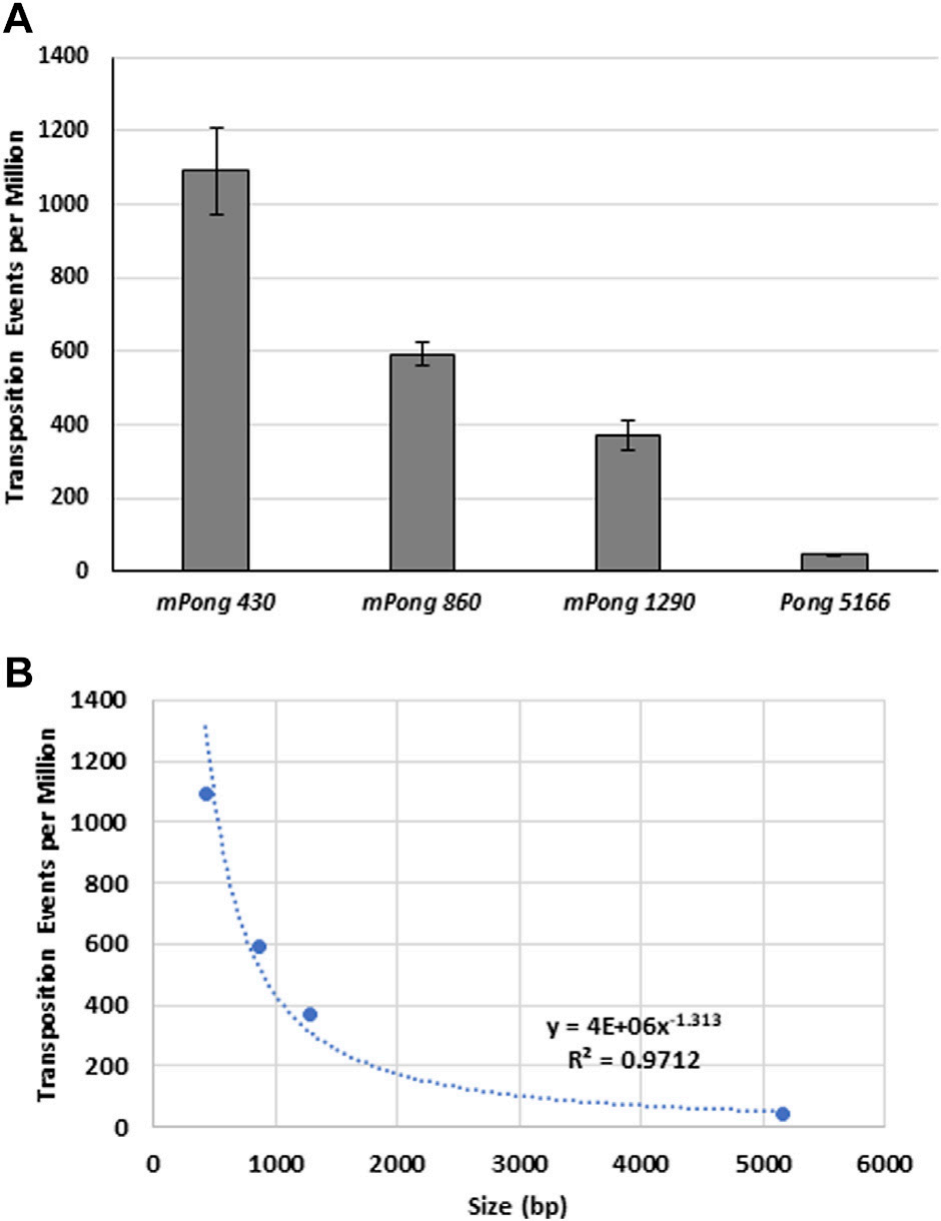
The effect of element size on excision frequency. **(A)** Yeast transposition assay with *mPong* elements of differing sizes on a pWL89a plasmid. Size in bp is indicated in the element name. *ORF1* was expressed from pAG 413 GAL and *TPase* was expressed from pAG 425 GAL. Error bars represent the standard error of six replicates. **(B)** Graph showing the correlation between element size and transposition frequency.

The greater abundance of small elements, especially *Tourist* MITEs, has led to the hypothesis that small *PIF*/*Pong*/*Harbinger* elements transpose more frequently (Feschotte et al., 2002; Zhang et al., 2004). Previous yeast transposition assays were used to show that *mPing* (430 bp) transposes at significantly higher frequency than the larger *Ping16A* (5,341 bp) element (Redd et al., 2023). To further test the effect of size on element excision frequency, we performed transposition assays with synthetic *mPong* elements ranging from 430 bp to 5,166 bp in size (Figure 3). We observed a direct correlation (*R* ^2^ = 0.97) between the element size and the number of *ADE2* revertant colonies. This suggests that increasing the size of the elements decreases the ability of the ORF1 and TPase proteins to bind to the element and induce excision.

### 2.4 Element size effects insertion frequency

**FIGURE 4:**
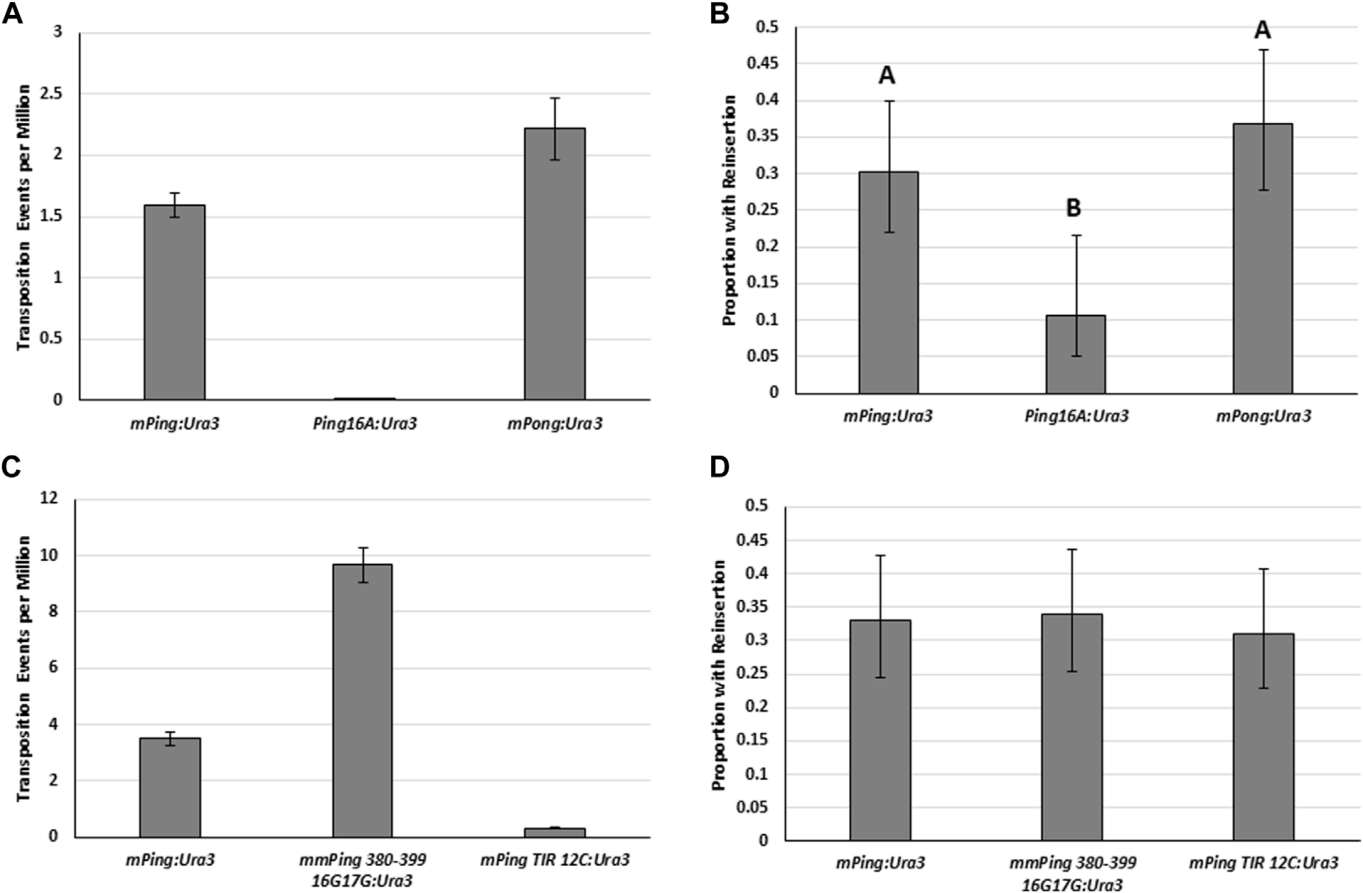
Excision and insertion frequency for *URA3* containing elements. **(A)** Yeast transposition assay for *URA3* containing versions of *mPing* (2,254 bp), *Ping16A* (7,126 bp), and *mPong* (2,254 bp) integrated into the genomic *ADE2*. A low copy plasmid (pAG 413 GAL) was used to express *ORF1:T2A:TPase*. Error bars represent the standard error of six replicates. **(B)** Proportion of *ADE2* revertant colonies that retained *URA3* as measured by the ability to grow on plates lacking uracil. Unique letter indicates statistical difference (*p* < 0.05, d.f. = 2) indicated by Chi^2^ analysis. **(C)** Yeast transposition assay for *URA3* containing versions of *mPing*, *mmPing 380–399 16G17G*, and *mPing TIR 12C* (all 2,254 bp) integrated into the genomic *ADE2*. A low copy plasmid (pAG 413 GAL) was used to express *ORF1:T2A:TPase*. Error bars represent the standard error of six replicates. **(D)** Proportion of *ADE2* revertant colonies that retained *URA3* as measured by the ability to grow on plates lacking uracil.

Although element excision is a good indicator of overall transposition activity, excision is not directly linked to insertion back into the genome. Thus, it is important to also determine the rate at which various *PIF*/*Pong*/*Harbinger* elements insert into the genome after excision. We developed yeast strains containing genomic copies of the *mPing*, *Ping16A* and *mPong* elements with inserted *URA3* selectable markers (*mPing:URA3*, *Ping16A:URA3*, *mPong:URA3*), allowing us to easily detect the elements. As expected, increasing the size by addition of the *URA3* selectable marker reduced the number of *ADE2* revertant colonies for all three elements, but the smaller *mPing:URA3* and *mPong:URA3* elements showed significantly higher excision than *Ping16A:URA3* (Figure 4A). To measure element insertion, the *ADE2* revertant colonies were isolated and tested for retention of the *URA3* gene (Figure 4B). This result shows that the larger *Ping16A:URA3* element (7,126 bp) was lost at a significantly higher frequency than the smaller *mPing:URA3* and *mPong:URA3* elements (2,258 bp). To confirm these results, we repeated these experiments with the natural *mPing*, *Ping16A*, and *Pong* elements encoded on a *URA3* containing plasmid. The insertion rate was tested by isolating *ADE2* revertant colonies, counter selecting against the *URA3* gene to remove the original plasmids, then PCR screening to detect genomic copies of the element. Table 1 shows that the proportion of *ADE2* revertant colonies that retained *mPing* was close to 100%, while the larger *Pong* and *Ping16A* elements were lost 25%–50% of the time.

**TABLE 1:** Observed yeast excision and insertion for natural rice transposable elements.

Element	Size (bp)	Excision frequency	Proportion inserted
*mPing*	430	459.7 ± 25.3	0.99
*Ping16A*	5,341	2.7 ± 0.5	0.47
*Pong*	5,166	111.7 ± 9.4	0.75

**FIGURE 5:**
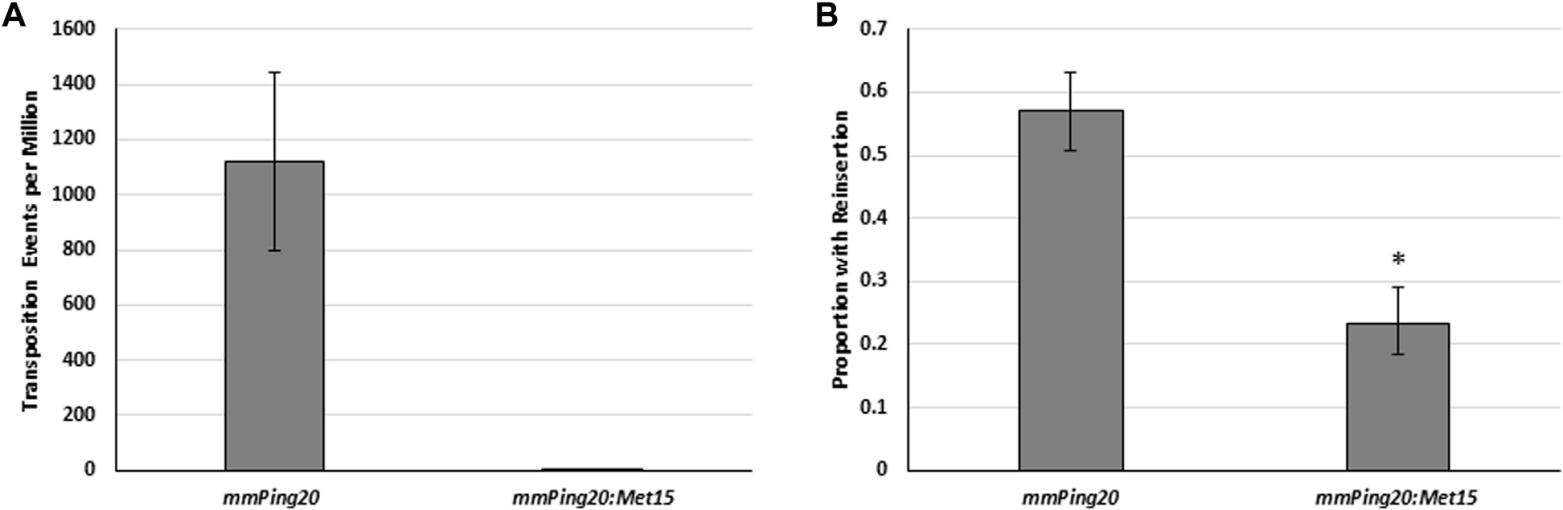
The effect of element size on insertion frequency. **(A)** Yeast transposition assay using genomic *mmPing20* (430 bp) and *mmPing20:MET15* (2,061 bp) *ADE2* reporters. pAG 426 GAL was used to express *ORF1:T2A:TPase*. Error bars represent the standard error of six replicates. **(B)** Proportion of *ADE2* revertant colonies that retained *mmPing20* as measured by PCR. Asterix indicates statistical difference (*p* < 0.05, d.f. = 1) indicated by Chi^2^ analysis.

We hypothesized that the observed differences in insertion ability could be due to differences in transposition complex stability or to the increased potential for self-insertion associated with longer DNA sequence. To address element stability, we tested the insertion frequency of *URA3* versions of previously described hyperactive (*mmPing 380–399 16G17G:URA3*) and hypoactive (*mPing TIR 12C:URA3*) elements that have TIR mutations that are thought to affect protein binding (Redd et al., 2023). Although we observed the expected differences in excision frequency (Figure 4C), we observed no difference in insertion (Figure 4D). This result indicates that there is no direct correlation between excision and insertion efficiency and suggests that once the element has excised its insertion is controlled by other factors. To further explore the effect of size, we compared the insertion frequency of genomic copies of the *mmPing20* (430 bp) and *mmPong20:MET15* (2,361 bp) element using PCR analysis (Figure 5). These results supported the model that larger elements fail to reinsert into the genome as efficiently as smaller elements because they are likely to insert into themselves after excision.

### 2.5 Excision site repair and insertion in diploid yeast

**FIGURE 6:**
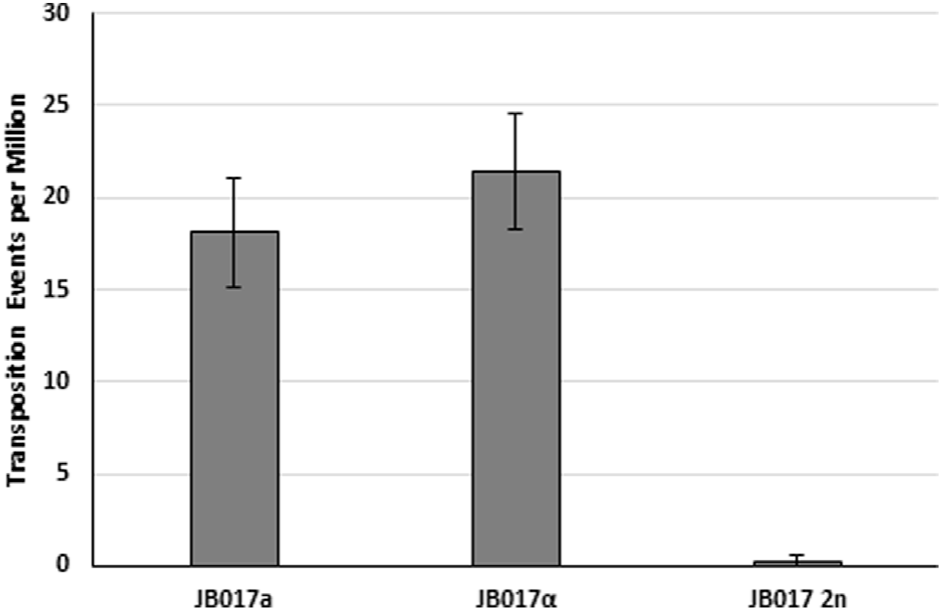
Excision frequency and *ADE2* repair in haploid and diploid yeast. Yeast transposition assay of *mPing* using pWL89a plasmid reporters in haploid (JB017a and JB017α) and diploid (JB017 2n) yeast. A low copy plasmid (pAG 413 GAL) was used to express *ORF1:T2A:TPase*. Error bars represent the standard error of six replicates.

All the previously described experiments were performed in haploid yeast strains, but most of the life cycle of plants is in the diploid state. Thus, we performed yeast transposition assays in diploid yeast created from our haploid strains. Using a plasmid reporter, we see a drastic reduction in excision frequency for the diploid compared to the a and α parent strains (Figure 6). We attribute this reduction in *ADE2* revertant colonies to differences in the repair of the *mPing* excision site. Diploid yeast is known to rely more on homology directed repair (HDR) instead of NHEJ for double strand break repair than haploids (Åström et al., 1999). Thus, we hypothesize that in diploid yeast the *mPing* excision sites are primarily being repaired using a homologous *mPing:ADE2* plasmid, which prevents formation of a functional copy of *ADE2*.

**FIGURE 7:**
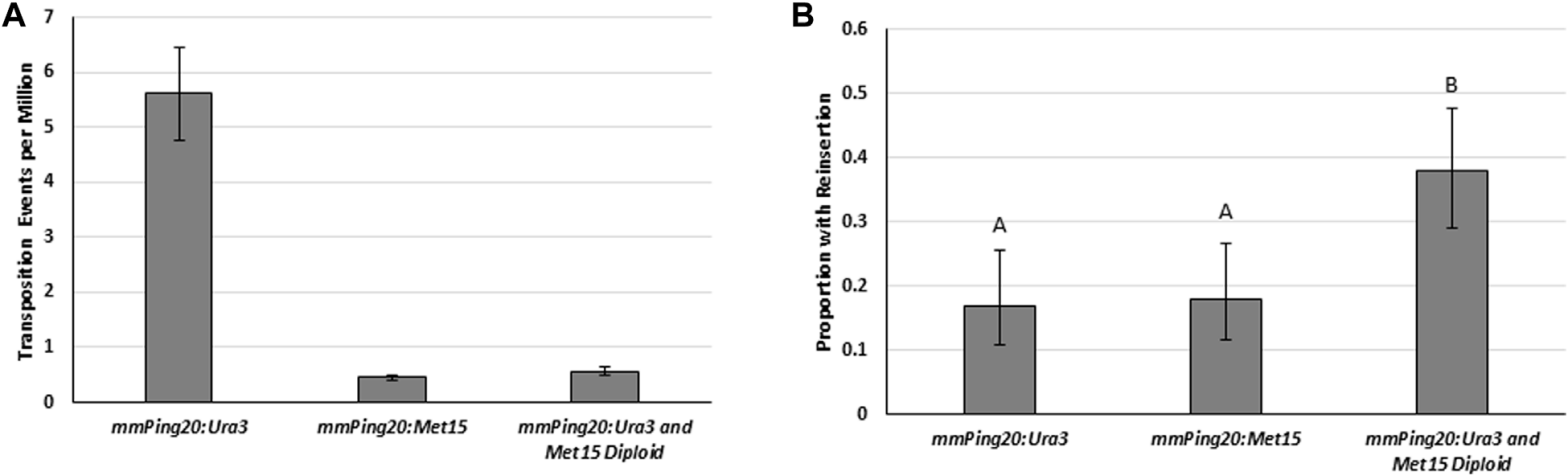
Excision and insertion frequency in haploid and diploid yeast. **(A)** Yeast transposition assay comparing haploid JB017a with genomic *mmPing20:URA3* (2,254 bp) and JB017α with genomic *mmPing20:MET15* (2061 bp) to the diploid JB017 2n made by mating the two haploids (contains both *mmPing20:URA3* and *mmPing20:MET15*). A low copy plasmid (pAG 413 GAL) was used to express *ORF1:T2A:TPase*. Error bars represent the standard error of six replicates. **(B)** Proportion of *ADE2* revertant colonies that retained the *URA3* and *MET15* containing elements as measured by the ability to grow on plates lacking uracil and methionine (diploid reinsertion indicates they retained both elements). Unique letter indicates statistical difference (*p* < 0.05, d.f. = 2) indicated by Chi^2^ analysis.

To test the effect of yeast ploidy on element reinsertion, we made a diploid yeast strain from haploid parents containing genomic copies of *mmPing20:URA3* (a mating type) and *mmPing20:Met15* (α mating type) inserted into the *ADE2* gene. Transposition assays with the haploid parents and the diploid (Figure 7A) showed that 2,254 bp *mmPing20:URA3* transposed at significantly higher frequency than the 2061 bp *mmPing20:Met15* element. This is likely due to base composition of the added sequences which has previously been shown to affect *mPing* transposition efficiency (Johnson et al., 2021; Redd et al., 2023). We also observed the expected drop in *ADE2* revertant colonies expected for the diploid. Analysis of the number of *ADE2* revertant colonies showed no difference in element retention between the two haploid yeast strains but the diploid strain had a higher rate of element retention (Figure 7B). Because the elements were similar sizes, this result is likely from the difference in the amount of homologous repair. One possible explanation for this result is that in the diploid yeast, the *ADE2* revertant colonies experienced multiple transposition events. If a transposition event was first repaired by homologous repair, followed by a second event repaired by NHEJ, it would provide an additional opportunity for the *mmPing20* element to successfully insert into the genome.

### 2.6 Insertion site preference and replicative transposition in yeast

**FIGURE 8:**
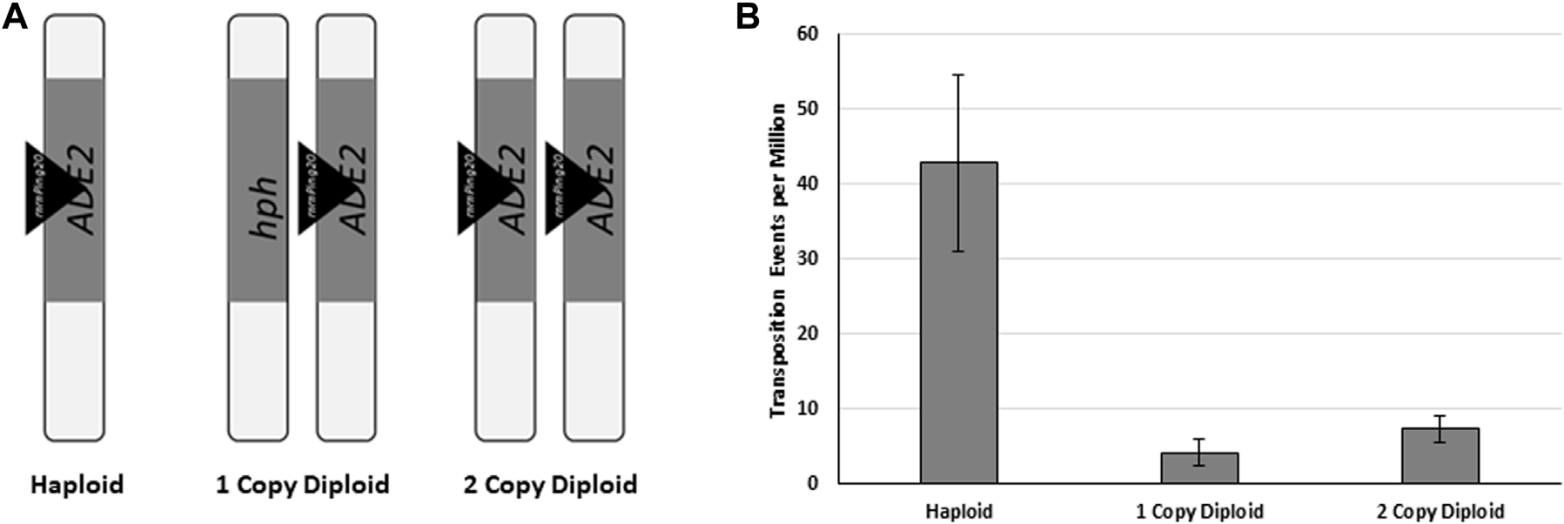
The effect of homologous templates. **(A)** Depiction of the *ADE2* locus of the genomic *mmPing20* yeast strains used. Vertical bars represent individual chromosomes, triangles represent inserted *mmPing20* elements. **(B)** Yeast transposition assay with the strains shown in **(A)**. *ORF1* was expressed from pAG 413 GAL and *TPase* was expressed from pAG 425 GAL. Error bars represent the standard error of six replicates.

The potential for HDR mediated gene conversion of the *mPing* excision sites in yeast provides an opportunity to test if HDR can act as the mechanisms for replication of class II TEs. In this replication model, there must be at least two matching chromosomes with identical class II elements at a particular locus. This could be from homologous chromosomes, or from sister chromatids after S-phase of the cell cycle. When one of these chromosomes has excision of one transposable element, the resulting excision site can be repaired by synthesis-dependent strand annealing or double-Holiday junction HDR (Ramakrishnan et al., 2018), potentially generating an additional copy of the element. To test if HDR-facilitated replication was occurring in our yeast, two diploid yeast strains containing the genomic *ADE2:mmPing20* reporter were developed (Figure 8A). The first (1 Copy Diploid) contained a single copy of the *ADE2:mmPing20* reporter construct on one chromosome and the other homologous chromosome had the *ADE2* gene replaced by the *hph* gene. In this strain, gene conversion of the *mmPing20* excision site does not produce a functional *ADE2* allele, as the sequence including the *hph* gene acts as the HDR template for this strain. Reversion of *ADE2* requires minimal disruption of the coding region, thus, the 1 Copy Diploid strain primarily indicates the number of excision events that are repaired by NHEJ. The second strain (2 Copy Diploid) contains an *ADE2:mmPing20* reporter on both chromosomes. In this strain, HDR repair of the excision site with the homologous *mmPing20* template could result in *mmPing20* replication. However, the transposition assay measures *ADE2* repair, so the number of colonies observed is still a measure of the excision events that are primarily repaired by NHEJ. Performing *ADE2* reversion assays with these strains showed that similar to previous results, the *ADE2* reversion frequency for both of the diploid strains was significantly lower than the haploid control (Figure 8B), consistent with them utilizing HDR repair to resolve the *mmPing20* excision sites. The finding that the 2 Copy Diploid shows a slightly higher rate of *ADE2* reversion than the 1 Copy Diploid is consistent with the increased excision observed when more element copies are present.

The *mmPing20* insertion sites from these haploid and diploid yeast strains were identified by sequencing *mPing20* containing amplicons from pooled genomic DNA from *ADE2* revertant colonies (4 pools from each strain, 12 colonies per pool). We used a previously established protocol that randomly shears the genomic DNA, ligates the fragments to pGEM plasmid, and then amplifies the desired fragments using primers anchored in *mmPing20* (Kanizay et al., 2015). This process resulted in the identification of 134 unique insertions that were mapped to the yeast genome (Supplementary Files S1). A significant percentage (haploid - 41.8%, 1 Copy diploid - 40.4%, and 2 Copy Diploid - 52%) of the *mmPing20* elements were identified at various locations in the rDNA repeat region on Chromosome XII (about 150 tandem copies of a 9.1 Kb sequence). The number of *mmPing20* insertions for each pool showed no statistically significant differences (Supplementary Table S1). All three yeast strains had at least one example of pools with more than the expected 12 copies of *mmPing20*. However, only Haploid and 1 Copy Diploid pools had examples with fewer *mmPing20* copies than the expected 12. The highest number of insertions detected (14) was in a 2 Copy Diploid DNA pool (samples 37–48). While we cannot ensure that our sequencing results detected every copy of *mmPing20*, this result suggests that *mmPing20* copies may be being lost at higher rates in the Haploid and 1 Copy Diploid strains.

**FIGURE 9:**
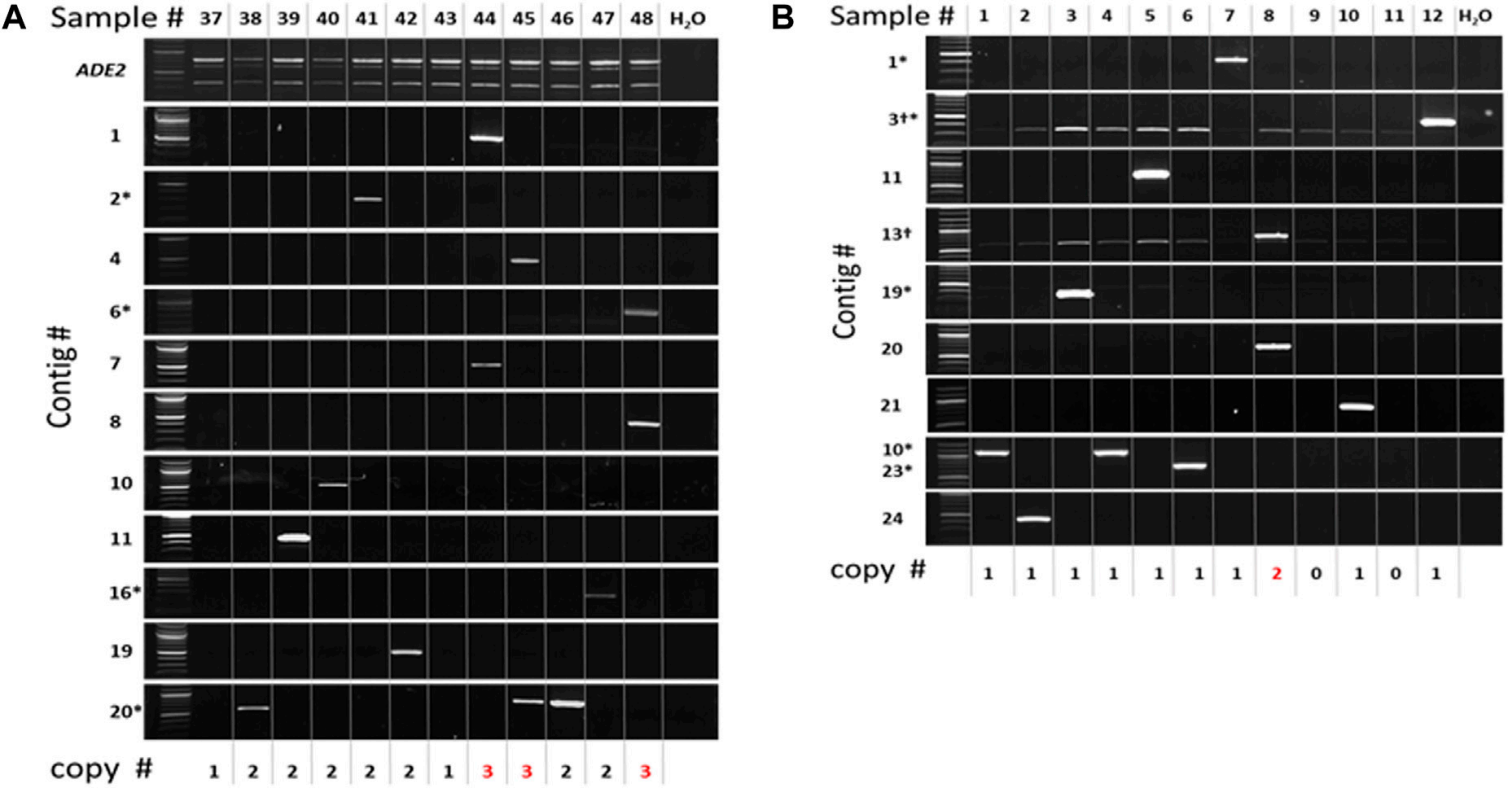
PCR Analysis of Selected *ADE2* Revertant Clones. PCR verification of insertions identified by sequencing. Numbers at the bottom indicate the total number of *mmPing20* elements detected by PCR. Red numbers indicate lines with evidence for replicative transposition. Asterisks indicate insertion in the Chromosome XII hotspot. **(A)** 2 Copy Diploid 37–48 pool. Top gel panel indicates the *ADE2* gene, upper band is *mmPing20* containing, lower band is ADE2 gene after excision. **(B)** 1 Copy Diploid 1–12 Pool.

To gain further insight, the individual *ADE2* revertant colonies from two selected pools [2 Copy Diploid 37–48 (Figure 9A) and 1 Copy Diploid 1–12 (Figure 9B)] were analyzed by PCR with primers flanking insertions identified by sequencing (Supplementary Table S2). As expected, PCR of the *ADE2* site showed that all 2-copy diploid isolates still contained one non-mobilized copy of *mmPing20* at the *ADE2* locus (Figure 9A). Using insertion site-specific primers with an *mmPing20* specific primer (Supplementary Table S2) resulted in appropriately sized amplicons for 11 of the 14 insertions indicated by sequence analysis. Though intended to be insertion site specific, multiple insertion site primers amplified DNA from more than one sample. The non-specific binding of these primers is consistent with multiple *mmPing20* insertions in the chromosome XII rDNA hotspot. Importantly, the PCR confirmation showed that three samples (44, 45, and 48) contained *mmPing20* insertions at two new sites in addition to the original *ADE2:mmPing20* (Figure 9A). This provides evidence that replicative transposition of *mmPing20* can occur in the 2 Copy Diploid strain. This analysis also revealed that two of the samples (37 and 43) did not contain mobilized copies of *mmPing20* despite evidence that it had excised from the *ADE2* reporter (Figure 9A). This is consistent with our other results showing that *mmPing20* does not always insert into the genome after excision. For the 1 Copy Diploid 1–12 pool (Figure 9B), ten of the 13 transposed *mmPing20* copies indicated by sequencing were verified by PCR. Two of the *ADE2* revertants did not contain any *mmPing20* insertion, consistent with the previously described insertion failure. A single sample, clone 8, contained an additional copy of *mmPing20*, suggesting that *mPing20* replication events are also possible in diploids that do not have homologous chromosomes with identical *mPing* insertions.

## 3 Discussion

Our results indicate that like other class II TE superfamilies, increasing the expression of ORF1 and TPase protein in yeast reduced the excision frequency of the large *Ping16A:URA3* element (Figure 1D). The presence of overexpression inhibition in both the *Mariner*/*Tc1* and *hAT* superfamilies is well documented and is thought to prevent DNA elements from reaching extremely high copy number (Bouuaert et al., 2013). For example, *Hsmar1* element transposition is inhibited at high TPase concentrations due to an allosteric effect controlled by a highly conserved linker motif between the N-terminal DNA-binding and C-terminal catalytic domain (Liu and Chalmers, 2014). Similarly, the *Ac* element TPase was found to oligomerize into a potentially catalytically inactive form at high concentrations (Heinlem et al., 1994). Using yeast transposition assays similar to those described in this paper, autoinhibition was detected when *Ac* TPase was expressed from the stronger GAL10 promoter, compared to expression from the weaker GALS promoter (Weil and Kunze, 2000). The absence of a detectable overproduction inhibition for the *mPing20* element in our yeast assays supports a model in which smaller elements, especially MITEs, avoid the production of unproductive transposition complexes that only contain one TIR because of the close proximity of the two ends of the element. Thus, the activity of the smaller *PIF/Pong/Harbinger* element relies on alternative methods to prevent excessive transposition. Previous studies suggest that protein localization, terminal inverted repeat sequences, and internal sequences play a role in regulating the activity of these small elements (Hancock et al., 2010; Payero et al., 2016; Redd et al., 2023).

Our observation that transposition increases as element copy number increases (Figure 2) is consistent with the expected dynamics of enzyme substrate binding. However, we recognize that in situations with limited protein expression, flooding the system with too many copies of the element could inhibit transposition complex formation. Thus, under most cases in the cell, transposition activity would be somewhat self-limiting. However, if large amounts of transposase expression are allowed to go unchecked, it would be possible in theory to get a rapidly growing copy number of small TEs that would be detrimental to the cell.

The experiments testing the effect of element size on *PIF*/*Pong*/*Harbinger* element transposition (Figure 3) are consistent with what has been observed for other class II elements. For example, experiments with the *Drosophila P-element* transformation (Roberts, 1986) and *in vitro Tc1* element transposition assays (Fischer et al., 1999) also indicated that small elements transpose more efficiently. Our results are also consistent with previous yeast transposition results showing that adding enhancer sequences to *mPing* resulted in significantly lower transposition (Johnson et al., 2021). We hypothesize that since both TIRs are part of the transposition complex, increasing the distance between the TIRs reduces the chances of interaction needed for transposition complex formation.

Our observations of *PIF/Pong/Harbinger* element insertion (Figure 4; Table 1) are consistent with what has been observed for other elements. The 627 bp *miniDs* element was reported to reinsert 57% of the time in yeast with either the wild-type or a hyperactive *Ac* Transposase protein (Lazarow et al., 2012). This is similar to the 50%–80% *Ac/Ds* insertion frequency reported for plants (Kunze et al., 1997). Similarly, the reinsertion frequency for the 2001 bp mini-*piggyBac* element in mouse cells with either a hyperactive or control transposase appears to be about 50% (Yusa et al., 2011). Additional experiments will be needed to confirm the role of self-insertion and determine if the effect of size on element insertion extends to other class II transposable elements.

Our finding that element insertion into the genome is correlated with element size has important implications to the replication and survival of *PIF/Pong/Harbinger* elements in the genome. It suggests that the abundance of smaller elements in the genome is not just due to increased excision frequency, but also due to increased genomic insertion. Thus, maintaining a small element size is a critical factor in overall TE replication strategy and may explain why most *PIF/Harbinger* elements do not carry excessive additional sequence outside of the coding regions. This finding also has implications for transposon tagging and genome editing applications, where addition of large cargos may lead to higher rates of element loss.

The results showing that the rDNA repeats are a common *mPing* insertion site is of interest as this region accounts for only 6.8%–12.7% of the genome (Kim et al., 2006). One possible explanation could be that this repetitive sequence can tolerate insertions better than other gene rich regions. However, a saturating mutagenesis of yeast using the *MiniDs* element showed that less than 7% of insertions mapped to the rDNA region (Michel et al., 2017). The insertions in this region were not site specific, suggesting it is not a sequence specific mechanism as observed for the *Pokey* element from *Daphnia* (Penton et al., 2002). Yeast rDNA sequences have previously been shown to be a hotspot for ectopic DNA integration (Tosato et al., 2005). However, our sequencing results indicated the presence of the normal target site duplications flanking the *mPing* insertion, suggesting that they were integrated by the normal catalytic mechanisms of the TPase protein. Analysis of the *mPing* insertion preference in rice (Naito et al., 2009) and soybean (Hancock et al., 2011) indicates that it primarily inserts upstream and downstream of genes and avoids GC rich sequences. However, no insertion hot spots have been observed in plants. The preference for yeast rDNA repeats suggests that these yeast sequences may have characteristics that mimic the normal insertion preference. Thus, this result warrants further study as it may provide more information about how the transposition complex identifies suitable insertion targets.

Though limited, our results provide some support for HDR mediated TE replication. Evidence for this gene conversion type replication mechanism has also been provided by experiments with the *Drosophila P* element where excision allowed for HDR of the double strand break, reverting the excision site to the sequence present on the homologous strand (Engels et al., 1990). This finding led the authors to predict that if chromosomes are homozygous for the TE insertion, excision and homologous repair of the excision site will result in the production of an additional copy of the element elsewhere in the genome. However, they had no mechanism to measure the copy number of the elements. Additional evidence for element replication by HDR comes from studies that found that deletion derivatives of the maize *Mudr* DNA element were derived from interrupted HDR (Plasterk, 1991; Hsia and Schnable, 1996). So, while there is support for this hypothesis, the experimental evidence is far from conclusive, and more information is needed about the frequency at which this phenomenon occurs.

## 4 Materials and methods

### 4.1 Yeast strains

All strains used in the study were derived from BY4741 (Baker Brachmann et al., 1998). JB017 (JIM17) yeast was described previously (Gilbert et al., 2015). To generate genomic reporter constructs, *URA3* or *MET15* containing elements with *ADE2* tail sequences were constructed by bridge fusion PCR or synthesized as DNA fragments. These fragments were transformed into yeast with selection for the selectable marker gene. PCR amplicons of *ADE2* were sequence verified to confirm proper insertion. To create versions lacking the internal *URA3* gene, selection on FOA was used to induce recombination of homologous flanking sequences.

α mating type strains were made by transforming with pGAL *HO* and growing on CSM-URA galactose media to induce mating type switching. Individual clones were obtained on FOA to remove pGAL *HO* and yeast mating type assays were performed by patching on lawns of WS199 Mat α and GWS340 Mat a yeast. Genotype was verified using Mat a and Mat α specific primers (White and Haber, 1990). To create diploids, Mat a strains were transformed with pAG413 and Mat α strains were transformed with pAG 415 before mating, selection on CSM-His-Leu, and PCR verification.

### 4.2 Plasmid construction

Bridge fusion PCR with primers that contain the T2A sequence and attB sequences were used to generate the *TPase:T2A:ORF1* and *ORF1:T2A:TPase* constructs. These were cloned into pDONR ZEO using a BP Clonase reaction. The *LacZ* gene was from pDONR223_LacZ, a gift from David Root [Addgene plasmid # 25893 (Yang et al., 2011)]. Genes were transferred to pAG 413 GAL-ccdb and pAG 423 GAL-ccdb, gifts from Susan Lindquist (Addgene plasmids # 14141 and # 14149) by performing a LR Clonase reaction.

### 4.3 Yeast transposition assays

Assays were performed as previously described (Redd et al., 2023). Briefly, liquid cultures were grown for 24 h before plating 100 μL directly on galactose media lacking adenine, and grown for 10 days at 30°C. For Figure 2, 100 μL of a 10^−2^ dilution was used to reduce the number of colonies to a countable range. To increase the number of *ADE2* revertant colonies in Figures 4, 5, 7, a 5 mL culture was spun down and resuspended in 400 μL before plating on a 150 mm plate and allowed to incubate for 15 days. The quantity of yeast plated was determined by plating 100 μL of the 10^−4^diluted cultures onto YPD.

### 4.4 Determining element retention

*ADE2* revertant colonies were patched out onto CSM-ADE plates to separate them from the yeast that did not experience transposition. For detection of *URA3* and *MET15* containing elements, clones were subsequently replica streaked onto CSM-URA and CSM-MET plates. For PCR detection, yeast was treated with Zymolyase prior to amplification with element specific primers.

### 4.5 DNA purification and sequencing

Yeast genomic DNA was purified from a fresh 3 mL liquid culture by washing in water and resuspending in 400 μL of lysis buffer (100 mM Tris pH 8.0, 50 mM EDTA, 1% SDS) before vortexing with acid washed glass beads. The liquid was transferred to a new tube, 250 μL of 7 M Ammonium Acetate pH 7.0 was added, incubated for 5 min at 65°C and then 5 min on ice before adding 500 μL of chloroform and centrifuging for 2 min. The resulting supernatant was transferred to a fresh tube, 950 μL of isopropanol was added, incubated for 5 min at room temperature, and centrifuged for 5 min to pellet the DNA. The pellet was washed with 70% ETOH, dried, and then resuspended in 50 μL of TE buffer. DNA was quantitated and pooled in groups of twelve before sequencing using a modified version of the procedure described previously (Kanizay et al., 2015). Sequence reads were de-multiplexed using Illumina BaseSpace (San Diego, CA). Trimmomatic (Bolger et al., 2014) was used to quality filter sequences. Sequences that contained the *mPing* target site duplication (TAA/TTA) were selected and the *mmPing20* element sequence was removed. The remaining sequences were assembled into contigs using Cap3 (Huang and Madan, 1999). BLAST searching was used to map the resulting contigs to the *Saccharomyces cerevisiae* genome (Goffeau et al., 1997). The sequencing reads are available at NCBI (BioProject ID: PRJNA949205).

### 4.6 PCR verification of *mmPing20* insertions

Sequencing data was used to design primers approximately 500 bp upstream or downstream of the target site duplication of each unique insertion site. These primers (Supplementary Table S2) were used with *mPing* primers (*mPing* 41 Rev or *mPing* 403 For) to screen DNA from individual *ADE2* revertant colonies. Retention of an *ADE2* inserted *mPing* copy was tested using the *ADE2* CF and *ADE2* CR primers (Hancock et al., 2010).

## Data Availability

The datasets presented in this study can be found in online repositories. The names of the repository/repositories and accession number(s) can be found below: https://www.ncbi.nlm.nih.gov/bioproject/?term=PRJNA949205.
