# Supplementary material for: Transposase expression, element abundance, element size, and DNA repair determine the mobility and heritability of PIF/Pong/Harbinger transposable elements
Source: Front Cell Dev Biol. 2023 Jun 9;11:1184046. doi: 10.3389/fcell.2023.1184046 (PMC10288884; doi:10.3389/fcell.2023.1184046)
Supplement: Supplementary file 1 [file Table1.DOCX]

Supplementary Material

Transposase expression, element abundance, element size, and DNA repair determine the mobility and heritability of *PIF*/*Pong*/*Harbinger* transposable elements

Priscilla S. Redd, Lisette Payero, David Gilbert, Clinton A. Page, Reese King, Edward McAssey, Dalton Bodie, Stephanie Diaz, C. Nathan Hancock^*^

*** Correspondence:** C. Nathan Hancock: nathanh@usca.edu

**Supplementary Table 1.** Number of inserted *mmPing20* elements in *ADE2* revertant colonies detected by sequencing of pooled DNA.

| **Pooled DNA** | **Number of non-redundant *mmPing20* insertions** | **Number of reads with *mmPing20* and TSD sequence** |
| --- | --- | --- |
| 1 Copy Diploid 1-12 | 13 | 64745 |
| 1 Copy Diploid 13-24 | 11 | 7638 |
| 1 Copy Diploid 25-36 | 12 | 21246 |
| 1 Copy Diploid 37-48 | 10 | 5920 |
| 1 Copy Haploid 1-12 | 12 | 7585 |
| 1 Copy Haploid 13-24 | 12 | 9553 |
| 1 Copy Haploid 25-36 | 13 | 6716 |
| 1 Copy Haploid 37-48 | 9 | 17335 |
| 2 Copy Diploid 1-12 | 13 | 3343 |
| 2 Copy Diploid 13-24 | 12 | 2483 |
| 2 Copy Diploid 25-36 | 12 | 3173 |
| 2 Copy Diploid 37-48 | 14 | 3434 |

**Supplementary Table 2.** Primer names and sequences.

| **Primer** | **Sequence** |
| --- | --- |
| Sample 12 Insertion site 1 For | TGTAGCAAGAAGACGAAATCCAGA |
| Sample 12 Insertion site 2 For | TAGGAATTCCTCGTTGAAGAGCAA |
| Sample 12 Insertion site 4 For | TGGCCAGAATGACGCAAATAATTT |
| Sample 12 Insertion site 6 For | TAGCGAACAAGTACAGTGATGGAA |
| Sample 12 Insertion site 7 For | GAACCCGAGAGCAGGAATTAAATG |
| Sample 12 Insertion site 8 For | CCTCAATTTTCTTGGCCAAAAAGC |
| Sample 12 Insertion site 10 For | ACTGTGTAGTGTCAGTAAGTGTCC |
| Sample 12 Insertion site 11 Rev | GGTAGAAACCATTGTATCTGTGCG |
| Sample 12 Insertion site 16 For | GTTGCCCTCTCCAAATTACAACTC |
| Sample 12 Insertion site 19 For | ACGATTACGGTACAGAGGATAACG |
| Sample 12 Insertion site 20 For | TTCGATCCCCTAACTTTCGTTCTT |
| Sample 5 Insertion site 1 For | TCTCGTTCGTTATCGCAATTAAGC |
| Sample 5 Insertion site 2 For | GAAGCCGAGAATTTTGTAACACCA |
| Sample 5 Insertion site 3 For | GAAGAGCTTTTAGTGGGCCATTTT |
| Sample 5 Insertion site 10 For | GCTTAATTGCGATAACGAACGAGA |
| Sample 5 Insertion site 11 For | ACTTGGGTTATTCTGATGAGGCTT |
| Sample 5 Insertion site 13 For | TCGAGAAAGTTGGAGTTTTTCAGC |
| Sample 5 Insertion site 19 For | ACTCATTCCAATTACAAGACCCGA |
| Sample 5 Insertion site 20 For | ACTTCCCTCATGTAGCACTTTGAT |
| Sample 5 Insertion site 21 For | TTTGGCAATAGCTTTTCGTGACTT |
| Sample 5 Insertion site 22 For | CAGGTGTGATTGAAGAGTTTGCAT |
| Sample 5 Insertion site 23 For | GGGGCATCAGTATTCAATTGTCAG |
| Sample 5 Insertion site 24 For | AACGTGCCCTCGTAAAAAGAAAAT |
| Sample 5 Insertion site 25 Rev | CACGTTAATGGCTCCTTTTCCAAT |
| *mPing* 41 Rev | TGCATGACACACCAGTGAAA |
| mPing 403 For | CGTGCAATGACACTAGCCAT |
| Mat a For | ACTCCACTTCAAGTAAGAGTTTG |
| Mat α For | GCACGGAATATGGGACTA CTTCG |
| D Mat Rev | AGTCACATCAAGATCGTTTATGG |
| *ADE2-*CF | GGGTTTTCCATTCGTCTTGAAGTCGAGGAC |
| *ADE2*-CR | CATTTCCACACCAAATATACCACAACCGGGA |

**Supplemental Files:** Contigs generated from *mmPing20* insertion site sequencing.

**Haploid_1-12.contigs**

>Contig1

ACGGTTTAGTGTTTTCTTACCCAATTGTAGAGACTATCCACCAGGAC

>Contig2

ATAAGAGAAATTGAAGAGCGCAACGAACTACGAGCATGGCTGGAGGAAAGATTCCTATTGTAGGAATTGTGGCATGTTTACAGCCGGAGATGGGGATAGGATTTCGTGGAGGTCTACCATGGAGGTTGCCCAGTGAAATGAAGTATTTCAGACAGGTCACTTCATTGACGAAAGATCCAGATCGGAAGAGCACACGTCTGAACTCCAGTCACATGACCAGAACTCGTATGCCGTCTTCT

>Contig3

ACTGAACCTAATTGGAATTTTACTAACAATATGTGGCACAATAGGATACGGATGGGCAATCTTCTTTCATTATCATTTTGTGGTTCTTTTAGTTTTTTCCGCTCTCACTGCCTTTGGTATGACCTGGTGCAGCAACACATCAATGACATATTTAACTGAGTTATTCCCCAAAAGAGCTGCTGGAACTGTTGCTGTTAGTAGTTTCTTTCGAAATGTGGAGATCGGAAGAGCACACGCT

>Contig4

ATACATGCTTAAAATCTCGACCCTTTGGAAGAGATGTATTTATTAGATAAAAAATCAATGTCTTCGGACTCTTTGATGATTCATAATAACTTTTCGAATCGCATGGCCTTGTGCTGGCGATGGTTCATTCAAATTTCTGCCCTATCAACTTTCGATGGTAGGATAGTGGCCTACCATGGTTTCAACGGGTGCACCGTCTGAACTCCAGTCACATGACCAGATCTCGTATGCCGTCTTCTGCTTGA

>Contig5

TGATCCTTCCGCAGGTTCACCTACGGAAACCTTGTTACGACTTTTAGTTCCTCTAAATGACCAAGTTTGTCCAAATTCTCCGCTCTGAGATGGAGTTGCCCCCTTCTCTAAGCAGATCCTGAGGCCTCACTAAGCCATTCAATCAGATCGGAAGAGCACACGTCTGAACTCCAGTCACATGACCAGAACTCGTATGCCGTCTTCTGCTTGAAAAAAAAAAAAAAAA

>Contig6

ACGGTTTAGTGTTTTCTTACCCAATTGAGATCGGAAGGAGCACACGTCTGAACTCCAGTCACATGACCAGATCTCGTATGCCGTCTTCTGCTTGAAAAAAAAAAAAAAAAAAA

>Contig7

ATGATCCTTCCGCAGGTTCACCTACGGAAACCTTGTTACGACTTTTAGTTCCTCTAAATGACCAAGTTTGTCCAAATTCTCCGCTCTGAGATGGAGTTGCCCCCTTCTCTAAGCAGATCCTGAGGCCTCACTAAGCCATTCAATCGGTACTAGCGACGGGCGGTGTGTACAAAGGGCAGGGACGTAATCAACGCAAGCTGATGACTTGCGCTTACTAGGAATTCCTCGTTGAAGAGCAACA

>Contig8

TAGCCAAGAAATGAGAAACGTGGGAGATGTTCAACCTAGCCTGATCC

>Contig9

TAGCCAAGAAATGAGCAACGTGGGAGATGTTCAACCTCGCCTGATCC

>Contig10

CTCCTCAAGGTAAAACTTGGCGTACGGAGAGGGGTCGAGAAAAGGTTCCGAGTGGAAATGTACGCCAATGTTTAGGGCTACGTGGAATGTGATCTCATTCTTACAAAACACGTAGATGATGAAAAGGGCCGCAAAAGTTTCTGTTATTCTTTAAATTGTTCCCAGAAATCCTCGAAGGAATGACCTAAATCTTAAGGCACAGATCGGAAGAGCACACGTCTGAACTCCATAACATGAC

>Contig11

TGCTCATGTGCTGCCAAATGGGTTCAAGATGAACAGTAAAGAACCTATTGACATCACTCCCCCTTCACAAAACAACAGATCGGAAGAGCACACGTCTGAACTCCAGTCACATGACCAGATCTCGTATGCCGTCTTCTGCTTGAAAAAAAAAAAAAAAAAA

>Contig12

GAAACAGCACTTATGAAGAAGATACTGCGATTAACCAAAATATTCTCCTCCTTATGAAAAAAAAAAAAGACTCAGAAACCTATGCAATCGTTTGCTAATATGTCGAAAAATGGATCTATTAAGAATATAAATTA

>Contig13

GAAACAGCACTTATGAAGAAGATACTGCGATTAACCAAAATATTCTCCTCCTTATGAAAAAAAAAAAAAAAACTCAGAAACCTATGCAATCGTTTCCTAATATGT

>Contig14

ACAAGCCTAGCAAGACCGCGCACTTAAGCGCAGGCCCGGCTGGACTCTCCATCTCTTGTCTTCTTGCCCAGTAAAAGCTCTCATGCTCTTGCCAAAACAAAAAAATCCATTTTCAAAATTATTAAATTTCTTTAATGATCCTTCCGCAGG

>Contig15

TACATGCTTAAAATCTCGACCCTTTGGAAGAGATGTATTTATTAGATAAAAAATCAATGTCTTCGGACTCTTTGATGATTCATAATAACTTTTCGAATCGCATGGCCTTGAGATCGGAAGAGCACACGTCTGAACTCCAGTCACATGACCAGATCTCGTATGCCGTCTTCTGCTTGAAAAAAAAAAAAAAAAAAAAACAAC

>Contig16

ACGGTTTAGTGTTTTCTAGATCGGAAGAGCACACGTCTGAACTCCAGTCACATGACCAGATCTCGTATGCCGTCTTCTGCTTGAAAAAAAAAAAAAAAAAC

>Contig17

ACGGTTTCGTGTTTTCTTACCCAATTGTAGAGACTATCCACAAGGAC

>Contig18

ACGGTTTAGTGTTTTCTTACCCAATTGTAGAGACTATCCACAAGGACAATATAGATCGGAAGAGCACACGTCTGAACTCCAGTCACATGACCAGATCTCGTATGCCGTCTTCTGCTTGAAAAAAAAAAAAAAAAACAAAAAA

>Contig19

TAGCCAAGAAATGAGAAACGTGGGCGATGTTCAACCTCGCCTGATCC

>Contig20

GACCAACAAGTAGCATTTTCTACTTGTGTTTTAGCGTTATTGGTCAACATTGGTAGGTTGAACACCACTATAAATTTTTATTTAGAAATGACATCGCAATTGAGAACTTTAGATCGGAAGAGCACACGTCTGAACTCCAGTCACATGACCAGATCTCGTATGCCGTCTTCTGCTTGAAAAAAAAAAAAAAAATA

>Contig21

CTGAACCTAATTGGAATTTTACTAACAATATGTGGCACAATAGGATACGGATAGATCGGAAGAGCACACGTCTGAACTCCAGTCACATGACCAGATCTCGTATGCCGTCTTCTGCTTGAAAAAAAAAAAAAACAAAACC

>Contig22

GACCAACAAGTAGCATTTTCTACTTGTGTTTTCGCGTTATTGGTCAAC

>Contig23

GACCAAAAAGTAGCATTTTCTACTTGTGTTTTAGCGTTATTGGTCAAC

>Contig24

TGCTCATGTGCTGCCAAATGGAGATCGGAAGAGCACACGTCTGAACTCCAGTCACATGACCAGATCTCGTATGCCGTCTTCTGCTTGAAAAAAAAAAAAAAAAACATACAT

>Contig25

TAGCCAAGAAATGAGAAACGTGGGAGATGTTCAACATCGCCTGATCC

>Contig26

TAGCCAAGAAATGAGAAACGTGGGAGATGTTCAACCTCGCCTGATCCAGATCGGAAGAGCACACGTCTGAACTCCAGTCACATGACCAGATCTCGTATGCCGTCTTCTGCTTGAAAAAAAAAAAAAAAAAAACAAAA

>Contig27

ATAGCCAAGAAATGAGAAACGTGGGAGATGTTCAACCTCGCCTGATCCTACCAAAAAAAAAAATCGCAAAAATAAGCGGGAAAAAATGTCAGATTCTAAGTTTCAAACCTCCGGAGATGATTCATCATCACCATCACCGCCGATTTGTTGGATTGCGTGTTTACGATCCATCATTTTTCTTGTAAGCGTTTAGCTTTCTGTCATCTCTTTATCTTTTTTCCTATTTTTTTTTTTTCCCTA

>Contig28

AGAAACAGCACTTATGAAGAAGATACTGCGATTAACCAAAATATTCTCCTCCTTATGAAAAAAAAAAAAAAAAACTCAGAAACCTATGCAATCGTTTGCTAATATTT

>Contig29

ACGGTTTAGTGTTTTCTTACCCAATTGTAGAGACTATCCACAAGGACAATATTTGTGACTTATGTTATGCGCCTGCTAGAGAGATCGGAAGAGCACACGTCTGAACTCCAGTCACATGACCAGATCTCGTATGCCGTCTTCTGCTTGAAAAAAAAAAAAAAAAAAA

>Contig30

TAGCCAAGAAATGAGAAACGTGGGAGATGATCAACCTCGCCTGATCC

>Contig31

ACGGTTTAGTGTTTTCTTACCCAATTGTAGAGACTATCCACAAGGACAATATTTGTGACTTATGTTATGCGCCTGCTAGAGTTCCGGACTCCGTTAGATCGGGAAGAGCACACGTCTGAACTCCAGTCACATGACCAGATCTCGTATGCCGTCTTCTGCTTGAAAAAAAAAAAAAAAAAAAAAAAAAAA

>Contig32

CTGAACCTAATTGGAATTTTACTAACAATATGTGGCACAAGATCGGAAGAGCACACGTCTGAACTCCAGTCACATGACCAGATCTCGTATGCCGTCTTCTGCTTGAAAAAAAAAAAAAAAAAAGAGGAAGACATCAATTAAAAAAAGTAG

>Contig33

ACGGTTTAGTGTTTTCTTACCCAATTGTAGAGACTATCCACAAGGACAATATTTGTGACTTATGTTATGCGCCTGCTAGAGTTCCGGACTCCGTTCAACTTAAGGCGAAGTTGTTGGCAGAAAATGCAATCAAATCTTTTCCCGGTTGTGGTATATTTGGTGTGGAAATGAGATCGGAAGAGCACACGTCTGAACTCCAGTCACATGACCAGATCTCGTATGCCGTCTTCTGCTTGAAAAA

>Contig34

ACGGTTTAGTGTTTTCTTACCCAATTGTAGAGACTATCCAGATCGGAAGAGCACACGTCTGAACTCCAGTCACATGACCAGATCTCGTATGCCGTCTTCTGCTTGAAAAAAAAAAAAACAAATACG

>Contig35

ACAGACCTCACAATCATGACTGCTAATTCTTTAGTAAATGGTGCCCATTTTTCGGCGTACAAAGGACGATCCTTCAGTACTTCCAAAGCTTCCGGAATCATTTCCTTATTCAGATCGGAAAGAGCACACGTCTGAACTCCAGTCACATGACCAGATCTCGTATGCCGTCTTCTGCTTGAAAAAAAAAAAAACAACA

>Contig36

ACGGTTTAGTGTTTTCTTAGATCGGAAGAGCACACGTCTGAACTCCAGTCACATGACCAGATCTCGTATGCCGTCTTCTGCTTGAAAAAAAAAAAAAAAAAA

>Contig37

AGAAACAGCACTTATGAAGAAGATACTGCGATTAACCAAAATATTCTCCTCCTTATGAAAAAAAAAAAAAAAAACTCAGAAACCTAGAACGGAAGAGCACACG

>Contig38

AACGGTTTAGTGTTTTCTTACCCAATTGTAGAGACTATCCACAAGGACAATATTTGTGACTTATGTTATGCGCCTGCTAGAGTTCCGGACTCCGTTCAACTTAAGGCGAAGTTGTTGGCAGAAAATGCAATCAAATCTTTTCCCGGTTGTGGTATAGATCGGAAGAGCACACGTCTGAACTCCAGTCACATGACCAGATCTCGTATGCCGTCTTCTGCTTGAAAAAAAAAAAAAAAAAAA

>Contig39

TGCTCATGTGCTGCCAAATGGGTTCAAGATGAACAGTAAAGAACCTATTGACATCACTCCCCCTTCACAAAACAACTACTTATCCCATGCTAGATCAGCTTCTTTCTCTAGATCGGAAGAGCACAGTCTGAACTCCAGTCACATGACCAGATCTCGTATGCCGTCTTCTGCTTGAAAAAAAAAAAAAAAAAAAAAAAAAAACA

>Contig40

GACCAACAAGTAGCATTTTCTACTTGTGTTTTAGCGTTATTGGACAAC

>Contig41

CTGAACCTAATTGGAATTTTACTAACAATATGTGGCACAATAGGATACGGATGGGCAATCTTCTTTCATTATCATTTTGTGGTTCTTTTAGTTTTTTCCGCTCTCACTGCCTTTGGTATGACCTGGTGCAGCAACACATCAATGACATATTTAACTGAGTTATTCCCCAAAAGAGATCGGAAGAGCACACGTCTGAACTCCAGTCACATGACCAGAACTCGTATGCCGTCTTCTGC

>Contig42

GACCAACAAGTAGCATTTTCTACTTGTGTTTTAGCGTTATTGGTCAACAGATCGGAAGAGCACACGTCTGAACTCCAGTCACATGACCAGATCTCGTATGCCGTCTTCTGCTTGAAAAAAAAAAAAAAAAAAA

>Contig43

GACCAACAAGTAGCATTTTCTACTTGTGTTTTAGCGGTATTGGTCAAC

>Contig44

GACCAACAAGTAGCATTCTCTACTTGTGTTTTAGCGTTATTGGTCAAC

>Contig45

TGATCCTTCCGCAGGTTCACCTACGGAAACCTTGTTACGACTTTTAGTTCCTCTAAATGACCAAGTTTGTCCAAATTCTCCGCTCTGAGATGGAGTTGCCCCCTTCTCTAAGCAGATCCTGAGAGATCGGAAGAGCACACGTCTGAACCAAATCACATGACCAGATCTCGTATGCCGTCTTCTACTTGAAAAAAAAAAAAAAAAA

>Contig46

GACCAACAAGAAGCATTTTCTACTTGTGTTTTAGCGTTATTGGTCAAC

>Contig47

TAACGGTTTAGTGTTTTCTTACCCAATTGTAGAGACTATCCACAAGGACAATATTTGTGACTTATGTTATGCGCCTGCTAGAGTTCCGGACTCCGTTCAACTTAAGGCGAAGTTGTTGGCAGAAAATAGATCGGAAAGAGCACACGTCTGAACTCCAGTCACATGACCAGATCTCGTATGCCGTCTTCTGCTTGAAAAAAAAAAAAAAAAAAAAAAAAAAAA

>Contig48

TAGCCACGAAATGAGAAACGTGGGAGATGTTCAACCTCGCCTGATCC

>Contig49

TAGCCAAGAAATGAGAAACGTGGGAGATGTTCAAACTCGCCTGATCC

>Contig50

ACGGTTTAGTGTTTTCTTACCCAATAGATCGGAAGAGCACACGTCTGAACTCCAGTCACATGACCAGATCTCGTATGCCGTCTTCTGCTTGAAAAAAAAAAAAAAAAAAA

>Contig51

GACCAACAAGTAGCATTTTCTACTTGTGTTTTAGCGTTATTGGTAAAC

>Contig52

TAGCCAAGAAATGCGAAACGTGGGAGATGTTCAACCTCGCCTGATCC

>Contig53

ACGGTTTAGTGTTTTCTTACCCAATTGTAGAGACAGATCGGAAGAGCACACGTCTGAACTCCAGTCACATGACCAGATCTCGTATGCCGTCTTCTGCTTGAAAAAAAAAAAAAAAAAAAAA

>Contig54

AACGGTTTAGTGTTTTCTTACCCAATTTTAGAGACTATCCACAAGGAC

>Contig55

GACCAACAAGTAGAATTTTCTACTTGTGTTTTAGCGTTATTGGTCAA

>Contig56

ATGCTCATGTGCTGCCAAATGGGTTCAAGATGAACAGTAAAGAACCTATTGACATCACTCCCAGATCGGAAGAGCACACGTCTGAACTCCAGTCACATGACCAGATCTCGTATGCCGTCTTCTGCTTGAAAAAAAAAAAAAAAAAAAACA

>Contig57

ATAGCCAAGAAATGAGAAACGTGGGAGATGTTCAACCTCGCCTGATCCTACCAAAAAAAAAAAATCGCAAAAATAAGCGGGAAAAAATGTCAGATTCTAAGTTTCAAACAGATCGGAAGAGCACACGTCTGAACCCCAGTAA

>Contig58

TAGCCAAGAAATGAGAAACGTGGGAGATGTTCAACCTCACCTGATCC

>Contig59

AATGCTCATGTGCTGCCAAATGGGTTCAAGATGAACAGTAAAGAACCTATTGACATCACTCCCCCTTCACAAAACAACTACTTATCCCATGCTAGATCAGCTTCTTTCTCTACCTACACGTCTCCTCCTCTGTCTGCACAAACGGAATTCTCTCACTCTGCTTCGAATGCAAACTACTTTTCCTCGCAATACCTAATGTATTCGCCTCAGAAAAGTCCAGAGGCCCTATATACTGAATTCT

>Contig60

ATGCTCATGTGCTGCCAAAGATCGGAAGAGCACACGTCTGAACTCAGTCACATGACCAGATCTCGTATGCCGTCTTCTGCTTGAAAAAAAAAAAAAAAAACAAT

>Contig61

ACGGTTTAGTGTTTTCTTACCCAATTGTAGAGACTATCCACAAGGACAATATTTGTGACTTATGTTATGCGCCTGCTAGAGTTCCGGACTCCGTTCAACTTAAGGCGAAGTTGTTGGCAGAAAATGCAATCAAATCTTTTCCCGGTTGTGGTATATTTGGTGTGGAAATGTTCTATTTAGAAACAGGGGAATTACATAGATCGGGAAGAGCACACGTCTGAACTCCAGTCACATGACCAGATCTCGTATGC

>Contig62

TAGACAAGAAATGAGAAACGTGGGAGATGTTCAACCTCGCCTGCTCC

>Contig63

AACGGTTTAGTGTTTTCTTACCCAATTGTAGAGACTATCCACAAGAGATCGGAAGAGCACACGTCTGAACTCCAGTCACATGACCAGATCTCGTATGCCGTCTTCTGCTTGAAAAAAAAAAAAAAAAAAAAAA

>Contig64

TAGCCAAGAAATGAGAAACTTGGGAGATGTTCAACCTCGCCTGATCC

>Contig65

CTGAACCTAATTGGAATTTTACTAACAATATAGATCGGAAGAGCACACGTCTGAACTCAGTCACATGACCAGATCTCGTATGCCGTCTTCTGCTTGAAAAAAAAAAAAAACACA

>Contig66

AAGGAAAAAAAAATAAAGAAAATATTGAAAACTAATAAGTTTTTTTCCTGTAAAAGTGTATAAAATATATAGGAGATCCCGTCAAATAATAGAAAAAAAATTTTTTGCCACATAGATCGGAAGAGCACAACGTCTGAACTCCAGTCACATGACCAGATCTCGTATGCCGTCTTCTGCTTGAAAAAAAAAAAAAAAAAAA

>Contig67

CAAGCCTAGCAAGACCGCGCACTTAAGCGCAGGCCCGGCTGGACTCTCCATCTCTTGTCTTCTTAGATCGGAAGAGCACACGTCTGAACTCCAGTCACATGACCAGATCTCGTATGCCGTCTTCTGCTTGAAAAAAAAAAAAAAAAAA

>Contig68

TTAGACCAACAAGTAGCATTTTCTACTTGTGTTTTAGCGTTATTGGTCAACATTGGTAGGTTGAACACCACTATAAATTTTTATTTAGAAATGACATCGCAATTGAGAACTTTTCACTCCGTGCCAGTTTTACAATTACATGCCAATGATCCGAAACTTTTGCAGGATACACCTAGACTGAAATCTATTCTGAAAAATCTGCCGTGGGGCAATGAGCAACTCTCATTGATGGAGACTTACAAAAA

>Contig69

TTAGAAACAGCACTTATGAAGAAGATACTGCGATTAACCAAAATATTCTCCTCCTTATGAAAAAAAAAAAAAAAAAACTCAAAAACCTATACAAGAAGAGCAAACCACTGA

>Contig70

TAGCCAAGAAATGAGAAACGTGGGAGAAGTTCAACCTCGCCTGATCC

>Contig71

AACGGTTTAGTGTTTTCTTACCCAATTGTAGAGACTATCCACAAGGACAATATTTGTGACTTATGTTATGCAGATCGGAAGAGCACACGTCTGAACTCCAGTCACATGACCAGATCTCGTATGCCGTCTTCTGCTTGAAAAAAAAAAAAAAAAAAAAA

>Contig72

ACTTCAGTTCCCGTTTTCGCATCAATGAGTAGCCGGAATATAAGTAGTTCCACTCAATATAAGAATAATTTCGATGCCTTGGCGACGCTTCCGTCTCCCTATAAATGTGCATGACACACCAGTGAAACCCCCATTGTGACTGGCCTTAACGGTTTAGTGTTTTCTTACCCAATTGTAGAGACTATCCACAAGGACAATATTTGTGACTTATGTTATGCGCCTGCTAGAGTTCCGGACTCCGTTCAACTTAAGGCGAAGTTGTTGGCAGAAAATGCAATCAAATCTTTTCCCGGTTGTGGTATATTTGGTGTGGAAATGTTCTATTTAGAAACAGGGGAATTGCTTATTAACGAAATTGCCCCAAGGCCTCACAACTCTGGACATTATACCATTGATGCT

>Contig73

AACGGTTTAGTGTTTTCTTACCCAATTGTAGAGACTATCCACAAGGACAATATTTGTGACTTATGTTATGCGCCTGCTAGAGTTCCGGAGATCGGAAGAGCACACGTCTGAACTCCAGTCACATGACCAGATCTCGTATGCCGTCTTCTGCTTGAAAAAAAAAAAAAAAAACAC

>Contig74

TAGCCAAGAAATGAGAAACGTGGGAGATGTTCAACCTCGCCTAATCC

>Contig75

ATGCTCATGTGCTGCCAAATGGGTTCAAGATGAACAGTAAAGAACCTATTGACATCACTCCCCCTTCACAAAACAACTACTTATCCCATGCTAGATCAGCTTCTTTCTCTACCTACACGTCTCCTCCTCTGTCTGCACAAACGGAATTCTCTCACTCTGCTTCGAATGCAAGATCGGAAGAGCACACGTCTGAACTCCAGTCACATGACCAGATCTCGTATGCCGTCTTCTGCTTGAAA

>Contig76

AGAAACAGCACTTATGAAGAAGATACTGCGATTAACCAAAATATTCTCCTCCTTATGAAAAAAAAAAAAAGACTCAGAAACCTATGCAATCGTTTGCTAATATGTCGAAAAA

**Haploid_13-24.contigs**

>Contig1

TTGTCACTACCTCCCTGAATTAGGATTGGGTAATTTGCGCGCCTGCTGCCTTCCTTGGATGTGGTAGCCGTTTCTCAGGCTCCCTCTCCGGAATCGAACCCTTATTCCCCGTTACCCGTTGAAACCATGGTAGGCCACTATCCTACCATCGAAAGTTGATAGGGCAGAAATTTGAATGAACCATCGCCAGCACAAGGCCATGCGATTCGAAAAGTTATTATGAATCATCAAAGAGACC

>Contig2

ACGGTTTAGTGTTTTCTTACCCAATTGTAGAGACTATCCACAAGGACAATATTTGTGACCTACTTTTCAGATAGGAAAGAGCACACGTCTGAACTCCAGTCACAACCGTTCATCTCGTATGCCGTCTTCTGCTTGAAAAAAAAAAAAAACAA

>Contig3

AACGGTTTAGTGTTTTCTTACCCAATTGTAGAGACTATCCACAAGGACAATATTTGTGACTTATGTTATGCGCCTGCTAGAGTTCCGGACTCCGTTCAACTTAAGGCGAAGTTGTTGGCAGAAAATGCAATCAAATCTTTTCCCGGTTGTGGTATATTTGGTGTGGAAATGTTCTATTTAGAAACAGGGGAATTGCTTATTAACGAAATTGCCCCAAGGCCTCACAACTCTGGACATTATACCATTAATGCTT

>Contig4

TTGTCACTACCTCCCTGAATTAGGATTGGGTAATTTGCGCGCCTGCTGCCTTCCTTGGATGTGGTAGCCGTTTCTCAGGCTCCCTCTCCGGAATCGAACCCTTATACACCGTTAAGATCGGAAGAGCACACGTCTGAACTCCAGTCACAACCGTTCATCTCGTATGCCGTCTTCTGCTTGAAAAAAAAAAAAA

>Contig5

ATTGAGTTTGGAAACAGCTGAAATTCCAGAAAAATTGCTTTTTCAGGTCTCTCTGCTGCCGGAAATGCTCTCTGTTCAAAAAGCTTTTACACTCTTGACCAGCGCACTCCGTCACCATACCATAGCACTCTTTGAGTTTCCTCTAATCAGGTTCCACCAAACAGATACCCCGGTGTTTCACGGAATGGTACGTTTGATATCGCTGATTTGAGAGGAGGTTACACTTGAAGAATCACAG

>Contig6

TTGTCACTACCTCCCTGAATTAGGATTGGGTAATTTGCGCGCCTGCTGCCTTCCTTGGATGTGGTAGCCGTTTCTCAGGCTCCCTCTCCGGAATCGAAACCTTATTCCCCGTTACCCGTTGAAACCATGGTAGAGATCGGAAGAGGCACACGTCTGAACTCCAGTCACAACCGTTCATCTCGTATGCCGTCTTCAGCTAGAAAAAAAAAAA

>Contig7

ACGGTTTAGTGTTTTCTTACCCAATTGTAGAGACTATCCACAAGGACAATATTTGTGACTTATGTTATGCGCCTGCTAGAGTTAGATCGGAAGAGCACACGTCTGAACTCCAGTCACAACCGTTCATCTCGTATGCCGTCTTCTGCTTGAAAAAAAAAAAAAA

>Contig8

AAAAACTTTCAACAACGGATCTCTTGGTTCTCGCATCGATGAAGAACGCAGCGAAATGCGATACGTAATGTGAATTGCAGAATTCCGTGAATCATCGAATCTTTGAACGCACATTGCGCCCCTTGGTATTCCAGGGGGCATGCCTGTTTGAGCGTCATTTCCTTCTCAAACATTCTGTTTGGTAGTGAGTGATACTCTTTGGAGTTAACTTGAAATTGCTGGCCTTTTCATTGGATGTTTTTTTT

>Contig9

ACGGTTTAGTGTTTTCTTACCCAATTGTAGAGACTATCCACAAGGACAATATTTGTGACTTATGTTATGCGCCTGCTAGAGTTCCGGACTCCGTTCAACTTAAGGCGAAGTTGTTGGCAGAAAATGCAATCAAATCTTTTCCCGGTTGTGGTATAGATCGGAAGAGCACACGTCTGAACTCCAGTCACAACCGTTCATCTCGTATGCCGTCTTCTGCTTGAAAAAAAAAAAAAAAAATA

>Contig10

ACGGTTTAGTGTTTTCTTACCCAATTGTAGAGACTATCCACAAGGACAATATTTGTGACTTATGTTATGCGCCTGCTAGAGTTCCGGACTCCGTTCAACTAGATCGGAAGAGCACACGTCTGAACTCCAGTCACAACCGTTCATCTCGTATGCCGTCTTCTGCTTGAAAAAAAAAAAAAAAACAACCAA

>Contig11

AATACATTCAAATATGTATCCGCTCATGAGACAATAACCCTGATAAATGCTTCAATAATATTGAAAAAGGAAGAGTATGAGTATTCAACATTTCCGTGTCGCCCTTATTCCCTTTTTTGCGGCATTTTGCCTTCCTGTTTTTGCTCACCCAGAAACGCTGGTGAAAGTAAAAGATGCTGAAGATCAGTTGGGTGCACGAGTGGGTTACATCGAACTGGATCTCAACAGCGGTAAGATCCT

>Contig12

TTGAGTTTGGAAACAGCTGAAATTCCAGAAAAATTGCTTTTTCAGGTCTTTACAGATCGGAAGAGCACACGTCTGAACTCAGTCACAACCGTTCATCTCGTATGCCGTCTTCTGCTTGAAAAAAAAAAAAACAAAAC

>Contig13

ATACATTCAAATATGTATCCGCTCATGAGACAATAACCCTGATAAATGCTTCAATAATATTGAAAAAGGAAGAGTATGAGTATTCAACAGATCAGATCGGAAGAGCACACATCTGAACTACAGTCACAACCGTTCATCTCGTATGCCGTCTTCTGCTTGAAAAAAAAAAAA

>Contig14

TAGCAACTATTTAAATAACTTAGATGTTTCTTTAACTTATTCCGTCATACGTACAGATCGGAAGAAGCACACGTCTGAACTCCAGTCACAACCGTTCATCTCGTATGCCGTCTTCTGCTTGAAAAAAAAAAAAAACAA

>Contig15

ATAGCAACTATTTAAATAACTTAGATGTTTCTTTAACTTATTCCGTCAGATCGGAAGAGCACACGCCGAAACCCAGTCACAACCGTTCATCTCGTATGCCGTCTTCTGCTTGAAAAAAAAAAAAAAA

>Contig16

ATAGCAACTATTTAAATAACTTAGATGTTTCTTTAACTTATTCCGTCATACGTACACAAACACCCGCTAGATATAGCACTCCTTGCTGTCGAACATTATAAAGGTGCTTTTAAAACTACTAATCGTATATTCAGCAGGTCAGAACGCAAAGTCGGACGATAAACTATGTTGAAGCTAGCTCGTCCATTTATTCCGCCTTTATCAAGGAACAATGCCATTTCTTCAGGAATAGTTCTCACTT

>Contig17

ATACATTCAAATATGTATCCGCTCATGAGACAATAACCCTGATAAATGCTTCAATAATATTGAAAAAGGAAGAGTATGAGTATTCAACATTTACGTGTCGCCCTTATTCCCTTTTTTAGATCGGAAGAGCACACGTCTGAACCCAGTCACAACCGTTCAACTCGTATGCCGTCTTCTGCTTGAAAAAAAAAACAA

>Contig18

ATACATTCAAATATGTATCCACTCATGAGACAATAACCCTGATAAA

>Contig19

ACGGTTTAGTGTTTTCTTACCCAATTGTAGAGACTATCCACAAGGACAATATTTGTGACTTATGTTATGCGCCTGCTAGAGTTCCGGACTCCGTTCAACTTAAGGCGAAGTTGTTAGATCGGAAGAGCACCACGTCTGAACTCCAGTCACAACCGTTCATCTCGTATGCCGTCTTCTGCTTGAAAAAAAAAAAAAAAAAAAA

>Contig20

AGTAACAAGGACTTCTTACATATTTAAAGTTTGAGAATAGGTCAAGGTCATTTCGACCCCGGAACCTCTAATCATTCGCTTTACCTCATAAAACTGATACGAGCTTCTGCTATCCTGAGGGAAACTTCGGCAGGAACCAGCTACTAGATGGTTCGATTAGTCTTTCGCCCCTATACCCAAATTCGACGATCGAT

>Contig21

AGACTTGAAGATTACAGGACTTTTTTTTTTCTTACTGTATTTTTCCGTAGAGTGCACCATTCGGCAGATGGATCGCGGGAGATCG

>Contig22

TACTCCTCAAGGTAAAACTTGGCGTACGGAGAGGGGTCGAGAAAAGGTTCCGAGTGGAAATGTACGCCAATGTTTAGGGCTACGTGGAATGTGATCTCATTCTTACAAAACACGTAGATGATGAAAAGGGCCGCAAAAGTTTCTGTTATTCTTTAAATTGTTCCCAGAAATCCTCGAAGGAATGACCTAAATCTTAAGGCACTTGCTTTTAAAACTATTGTTCGTTAATTTGTTAGTAAT

>Contig23

ACGGTTTAGTGTTTTCTTACCCAATTGTAGAGACTATCCACAAGGACAATATTTGTGACTTATGTTATGCGCCTGCTAGAGTTCCGGACTCCGTTCAACTTAAGGCGAAGTTGTTGGCAGAAAATGCAATCAAAAGATCGGGAAGAGCACACGTCTGAACTCCAGTCACAACCGTTCAACTCGTATGCCGTCTTCTGCTTGAAAAAAAAAAAAAAAACCA

>Contig24

AGAAGTCACAGACGGTAACGCAGCGCCTCGTTTCTAAATCGTATAGCAGGCTGAGCGCCCGCACACCCACCTGCGGGAGTTACTCCGGGGATCGTATGGGGTGATGCATGCTCTTTCGTAAACCTTAAGAATAGCGTTAATTTTTTCCTAAATAGGAATACACATACTCATGCTATGTAACTGTAGAGAAAGGTACAGATTACATGAGGCGCCGGTTGTCGTTGATACATATAGAACGG

>Contig25

ATATGGTAATGATTATACAACTAAAAAAAGATTAGCATTAAAAGCAATGTAATTAACATTATAATTTTTTTGGAAATGTGTGTATGTGAGTAAAGTATACAAGTTATGTGTGGTTTCTCATATTTTTATATTCTTAAAGTTTTATATTTATATTTTATATATCCGGGGAAGGAGAAGGGGAGATTTGATTGAATCTTCTTGCCTATGAAAGAAAGTGGCAGCAAAATGAAATGAGGCAA

>Contig26

ATGAATCATCAAAGAGTCCGAAGACATTGATTTTTTATCTAATAAATACATCTCTTCCAAAGGGTCGAGATTTTAAGCATGTATTAGCTCTAGAATTACCACAGTTATACCATGTAGTAAAGGAACTATCAAATAAACGATAACTGATTTAATGAGCCATTCGCAGTTTCACTGTATAAATTGCTTATACTTAGACATGCATGGCTTAATCTTTGAGACAAGCATATGACTACTGGCAGGATCAACCAGGTCGAAGAGCACACGTCTGAACTCCAGTCACGGAAGGATATCTCGTATGCCGTCTTCTGCTTGAAAAAAAAAAACAGAT

>Contig27

AGACTTGAAGATTACAGGACTTTTTTTTTTCTTACTGTATTTTTCCGTAGAGTGCACCATTCGGCAGATGGATCGCGGGGAAATTGAGAGTTTATGCAAAAAAATCCGAAGGTGGAATTAAATGGGTCCCGCTTTGGAAAGCCCCGGATTTAATCCCCCATTAAGGCAAATTACTCCTCATCCTAGGTGGAGAAAAGAACCGGAAAAAAATGCAGTACCTTTTTTGTTCCCATTTCTTTTCCA

>Contig28

AGGACTTTGAAAACTTTCTTGCGTAAATTCTGTTTAAGTCTGAAACTTTCAACTCTGTATTAAATTTGTTTTAAGATGTGCGACGGCGACGTAAAATGGTAAAGAAAAAGAAAATGAATGATAGTAAATCATACTGAAAACGAATGATCTGAAACAATAAAAAAAGATATTTGTATATAAATTATTCAGCCATGCTCAAACCTCCAGATTAAAAATCAATAATGGATTAATTGAAAGCC

**Haploid_25-36.contigs**

>Contig1

GTACTTATGTGCTTTATGAATGTGAATATTGTTACAGCTATGCAATGGTACAGATCGGAAGAGCACACGTCTGAACTCCAGTCACTCCAATCGATCTCGTATGCCGTCTTCTGCTTGAAAAAAAAAAAAAAAACA

>Contig2

CGGTATTTTACTATGGAATAATCAATCAATTGAGGATTTTATGCAAATATCGTTTGAATATTTTTCCGACCCTTTGAGTACTTTTCTTCATAATTGCATAATATTGTCCGCTGCCCCTTTTTCAGATCGGAAGAGCACACGTCTGAACTCAGTCACTCCAATCGATCTCGTATGCCGTCTTCTGCTTGAAAAAAAAAAAAACAAAACA

>Contig3

ACGGTTTAGTGTTTTCTTACCCAATTGTAGAGACTATCCACAAGAGATCGGAAGAGCACACGTCTGAACTCCAGTCACTCCAATCGATCTCGTATGCCGTCTTCTGCTTGAAAAAAAAAAAACAAA

>Contig4

TGTCTTATGTTACTCCTTGAACAATATAGCTGAAAGAGGGAAAAAGGAAAGTATATTTCCGAAAAAGGATGCCGAAGACGGCGCTATCATAATTATGTGTTTGAGAGGCAGTTCACGGTTACATTCTAGTTCTTCTAGCGTCTGTTGTAGTATGAAAAGAGATCGGAAGAGCACACGTCTGAACTCAGTCACTCCAATCGATCTCGTATGCCGTCTTCTGCTTGAAAAAAAAAAAAA

>Contig5

AGAAAAAATATAGAATAATAAATAATATGAAAAGGGAATGGTCAACAGTCATAGTCGCAATTTCTCTCAGGGCGAAATGGACTTTTTGAATGATGATGAACTAGACTTAGATTTACCGGTAACCGCAGAAATATCGAAAGAGTTATTCGCAACAGAAATAGAGAAGTATCGTGAATCCGAGACTAATGGGACCGATGTTGATAACTTTGATGTCGACAGATTCCTAGTGCAGAAAAATTTCCATTACC

>Contig6

GTAAAAAAAGAGCTGCGAATAGTAGCTTTCCGCCAATCAAACTCAAGAGCAGGACTAAGCTAGATAGTAGATCGGAAAGAGCACACGTCTGAACTCCAGTCACTCCAATCGATCTCGTATGCCGTCTTCTGCTTGAAAAAAAAAAAACAAAACA

>Contig7

ACGGTTTAGTGTTTTCTTACCCAATTGTAGAGACTATCCACAAGGACAATATTTGTGACTTATGTTATGCGCCTGCTAGAGTTCCGGACAGATCGGAAAGAGCACACGTCTGAACTCCAGTCACTCCAATCGATCTCGTATGCCGTCTTCTGCTTGAAAAAAAAAAAAAAAAA

>Contig8

AGGCAATCCCGGTTGGTTTCTTTTCCTCCGCTTATTGATATGCTTAAGTTCAGCGGGTACTCCTACCTGATTTGAGGTCAAACTTTAAGAACATTGTTCGCCTAGACGCTCTCTTCTTATCGATAACGTTCCAATACGCTCAGTATAAAAAAAGATTAGCCGCAGTTGGTAAAACCTAAAACGACCGTACTTGCATTATACCTCAAGCACGCAGAGAAACCTCTCTTTGGAAAAAAAAC

>Contig9

ACGGTTTAGTGTTTTCTTACCCAATTGTAGAGACTATCCACAAGGACAATATTTGTGACTTATAGATCGGAAGAGCACACGTCTAATCCAGTCACTCCAATCGATCTCGTATGCCGTCTTCTGCTTGAAAAAAAAAAAAGAA

>Contig10

ACGGTTTAGTGTTTTCTTACCCAATTGTAGAGGCTATCCACAAGGAC

>Contig11

GGGTCGGGTAGTGAGGGCCTTGGTCAGACGCAGCGGAGATCGGAAGAGGCACACGTCTGAACTCCAGTCACTCCAATCGATCTCGTATGCCGTCTTCTGCTTGAAAAAAAAAAAAACA

>Contig12

ACGGTTTAGTGTTTTCTTACCCAATTGTAGAGACTATCCACAAGGACAATATAGATCGGAAGAGCACACGTCTGAACTCCAGTCACTCCAATCGATCTCGTATGCCGTCTTCTGCTTGAAAAAAAAAAAAAAAAAAAAAGAC

>Contig13

ACGGTTTAGTGTTTTCTTACCCAATTGTAGAGACTATCCACAAGGACAATATTTGTGACTTATGTTATGCGCCTAGATCGGAAGAGCACACGTCTGAACTCCAGTCACTCCAATCGATCTCGTATGCCGTCTTCTGCTTGAAAAAAAAAAAAAAAAAA

>Contig14

ACGGTTTAGTGTTTTCTTACCCAATTGTAGAGACTATCCAAAAGGACAATATTTGTGACTTATGTTATGCGCCTGCTAGAGTTCCGGACTCCGTTCAACTTAAGGCGAAGTTGTTGGCAGAAAATGCAGATCGGGAAAGAGCACACGTCTGAACCCAGTCACTCCAATCGAACTCGTATGCCGTCTTCTGCTTGAAAAAAAAAAAAAAAAAA

>Contig15

AGGGTCGGGTAGTGAGGGCCTTGGTCAGACGCAGCGGGCGTGCTTGTGGACTGCTTGGTGGGGCTTGCTCTGCTAGGCGGACTACTTGCGTGCCTTGTTGTAGACGGCCTTGGTAAGATCGGAAGAGCACCACGTCTGAACTCCAGTCACTCCAATCGATCTCGTATGCCGTCTTCTGCTTGAAAAAAAAAAATAA

>Contig16

ACGGTTTAGTGTTTTCTTACCCAATTGTAGAGACTATCCACAAGGACAATATTTGTGACTTATGTTATGCGCCTGCTAGAGTTCCGGACTCCGTTCAACTAGATCGGAAGAGCACACGTCTGAACTCCAGTCACTCCAATCGATCTCGTATGCCGTCTTCTGCTTGAAAAAAAAAAAAAAA

>Contig17

AGTCTTTCGCCCCTATACCCAAATTCGACGATCGATTTGCACGTCAGAACCGCTACGAGCCTCCACCAGAGTTTCCTCTGGCTTCACCCTATTCAGGCATAGTTCACCATCTTTCGGGTCCCAACAGCTATGCTCTTACTCAAATCCATCCGAAGACATCAGGATCGGTCGATTGTGCACCTCTTGCGAGGCCCCAACCTACGTTCACTTTCATTACGCGTATGGGTTTTACACCCAA

>Contig18

GTACTTATGTGCTTTATGAATGTGAATATTGTTACAGCTATGCAATGGTACGATCATACTCTGAACGATCCATTTAAATTATATATGTCAAACAGAAGAGAAAGGTTTTTTTATTGAAGATTTCACAGAAAATCAATCATTTTGCAGATCGGAAGAGCACCACGTCTGAACTCCAGTCACTCCAATCGACATCGTATGCCGTCTTCTGCTTGAAAAAAAAAAACAAAAAAAAAACAAC

>Contig19

ACGGTTTAGTGTTTTCTTACCCAATTAGATCGGAAGAGCACACGTCTGAACTCCAGTCACTCCAATCGATCTCGTATGCCGTCTTCTGCTTGAAAAAAAAAAAAAAAAAC

>Contig20

ACGGTTTAGTGTTTTCTTACCCAATTGTAGAGACTATCCACAAGGACAATATTTGTGACTTATGTTATGCGCCTGCTAGAGTTCCGGACTCCGTTCAACTTAAGGCGAAGTTGTTGGCAGAAAATGCAATCAAATCTTTTCCCGGTTGGGTAAGAGCACACGTCTGAACTCCAGTCACTCCAATCGATCTCGTATGCCGTCTTCTGCTTGAAAAAAAAAAAAAAAAAA

>Contig21

GTCTTTCGCCCCTATACCCAAATTCGACGATCGATTTGCACGTCAGAAACGCTACGAGCCTCCACCAGAGTTTCCTCTGGCTTCACCCTATTCAGGCATAGTTCACCATCTTTCGGGTCCCAACAGATCGGAAGAGCACACGTCTACTCCAGTCACTCCAATCGATCTCGTATGCCGTCTTCTGCTTGAAAAAAAAAAACAACAACAAAAA

>Contig22

GTACTTATGTGCTTTATGAATGTGAATATTGTTACAGCTATGCAATGGTACGATCATACTCTGAACGATCCATTTAAATTATATATGTCAAACAGAAGAGAAAGGTTTAGATCGGAAGAGCACGTCTGAACTCCAGTCACTCCAATCGATCTCGTATGCCGTCTTCTGCTTGAAAAAAAAAAAAACAAATA

>Contig23

GAAGTGGATAACCAGCAAATGCTAGCACCACTATTTAGTAGGTTAAGGTCTCGTTCGTAGATCGGAAGAGCACACGTCTGAACTCCAGTCACTCCAATCGATCTCGTATGCCGTCTTCTGCTTGAAAAAAAAAAAAAACACAAA

>Contig24

GAAAAAATATAGAATAATAAATAATATGAAAAGGGAATGGTCAACAGTCATAGTCGCAATTTCTCTCAGGGCGAAATGGACTTTTTGAATGATGATGAACTAGAGATCGGAAGGAGCACACGTCTGAACTCCAGTCACTCCAATCGATCTCGTATGCCGTCTTCTGCTTGAAAAAAAAAA

>Contig25

ACGGTTTAGTGTTTTCTTACCCAATTGTAGAGACTATCCACAAGGACAATATTTGTGACTTATGTTATGAGATCGGAAAGAGCACACGTCTGAACTCCAGTCACTCCAATCGATCTCGTATGCCGTCTTCTGCTTGAAAAAAAAAAAAAAAAACA

>Contig26

ACGGTTTAGTGTTTTCTTACCCAATTGTAGAGACTATCCACAAGGACAATATTTGTGACTTATGTTATGCGCCTGCTAGAGTTCCGGACTCCGTTCAACTTAAGGCGAAGTTGAGATCGGAGAGAACACGTCTGAACTCCAGTCACTCCAATCGATCTCGTATGCCGTCTTCTGCTTGAAAAAAAAAACAATAACCA

>Contig27

GGGTCGGGTAGTGAGGGCCTTGGTCAGACGCAGTGGGCGTGCTTGT

>Contig28

GGGTCGGGTAGTGAGGGCCTTGGTCAGACGCAGCGGGCGTGCTTGTAGATCGGAAGAGCACACGTCTGAACTCCAGTCACTCCAATCGATCTCGTATGCCGTCTTCTGCTTGAAAAAAAAAAAAAAAA

>Contig29

ACGGTATTTTACTATGGAATAATCAATCAATTGAGGATTTTATGCAAATATCGTTTGAATATTTTTCCGACCCTTTGAGTACTTTTCTTCATAATTGCATAATATTGTCCGCTGCCCCTTTTTCTGTTAGACGGTGTCTTGATCTACTTGCTATCGTTCAACACCACCTTATTTTCTAACTATTTTTTTTTTAGCTCATTTGAATCAGCTTATGGTGATGGCACATTTTTGCATAAACCT

>Contig30

ACAATAAGTGCCGGGTCCTTCTTACTAGAGGTTAAAGAGGGTATCGCTGTCGGCGATTGATGGTACATATCTCTTATAACAGAATGGTTTGAAAAATCCGAAGGATGAAAATAACATACCATGTCAATTTTTATTGCGTAATTGCGAGATAGAATGTTGCCGATTGAAATCGACCACTCTCATACATTGGCAACTCAATATAGTACGCCAAGAATTGAGGAAGTTTCCTGAAATGACATACTTGATTT

>Contig31

GGGTCGGGTAGTGAGGGCCTTGGTCAGACGCAGCGGGCGTGCTTGTGGACTGCTTGGTGGGGCTTGCTCTGCTAGGCGGACTACTTGCGTGCCTTGTTAGATCGGAAGAGCACACGTCTGAACTCCAGTCACTCCAATCGATCTCGTATGCCGTCTTCTGCTTGAAAAAAAAAAAAAAAAA

>Contig32

AGAAGTGGATAACCAGCAAATGCTAGCACCACTATTTAGTAGGTTAAGGTCTCGTTCGTTATCGCAATTAAGCAGACAAATCACTCCACCAACTAAGAACGGCCATGCACCACCACCCACAAAATCAAGAAAGAGCTCTCAATCTGTCAATCCTTATTGTGTCTGGACCTGGTGAGTTTCCCCGTGTTGAGTCAAATTAAGCCGCAGGCTCCACTCCTGGTGGTGCCCTTCCGTCAATTCC

>Contig33

AGTACTTATGTGCTTTATGAATGTGAATATTGTTACAGCTATGCAATGGTACGATCATACTCTGAACGATCCATTTAAATTATATATGTCAAACAGAAGAGAAAGGTTTTTTTATTGAAGATTTCACAGAAAATCAATCATTTTGCAATATTCCTTCGTCCCAGAGAAGGTTTCGGCTTTTACAGCAACGAGAATGATTCTTCCATTATTTGTACCTAGCTTCCACATGGATTTCGATAAG

>Contig34

AAGGTTTAGTGTTTTCTTACACAATTGTAGATCGGAAGAGCACACGTCTGACTCCAGTCACTCCAATCGATCTCGTATGCCGTCTTCTGCTTGAAAAAAAAAAAAACAAAACAT

>Contig35

GAGCAGCGCGTTGCCTACAGAAACATCTTTATAAAATCTTGGCCGGCCAAGATTCCCAAACGAGACTCACATTTCCCAAAAAAGACATTTCTGTCCAAAAGTAGAAGGCAAGAAAACCCTGAGATCGGAAGAGCACACGTCTGAACTCCAGTCACTCCAATCGAACTCGTATGCCGTCTTCTGCTTGAAAAAAAAAAAAAAAAA

>Contig36

ACGGTTTAGTGTTTTCTTACCCAATTGTAGAGACTATCCACAAGGACAATATTTGTGACTTATGTTATGCGCCTGCTAGAGTTCCGGACTCCGTTCAACTTAAGGCGAAGTTGTTGGCAGAAAATGCAATCAAATCTTTTCCCGGTTGTGGTATATTTGGTGTGGAAAAGATCGGAAGAGCACACGTCTGAACTCAATCACTCCAATCGATCTCGTATGCCGTCTTCTGCTT

>Contig37

ACGGTTTAGTGTTTTCTTAGATCGGAAGAGCACACGTCTGAACTCCATCACTCCAATCGATCTCGTATGCCGTCTTCTGCTTGAAAAAAAAAAAAAAAAA

>Contig38

GGCAATCCCGGTTGGTTTCTTTTCCTCCGCTTATTGATATGCTTAAGAGATCGGAAGAAGCACACGTCTGAACTCCAGTCACTCCAATCGATCTCGTATGCCGTCTTCTGCTTGAAAAAAAAAAAACAAAAAACAA

>Contig39

ATGTCTTATGTTACTCCTTGAACAATATAGCTGAAAGAGGGAAAAAGGAAAGTATATTTCCGAAAAAGGATGCCGAAGACGGCGCTATCATAATTATGTGTTTGAGAGGCAGTTCACGGTTACATTCTAGTTCTTCTAGCGTCTGTTGTAGTATGAAAAGTACTTTATTGAATGTCCTTGACATGTTTTCCCAGGATCCAACTTAATGCTCCTCGCACTCATGCATGGGTCGGCCACACTGG

>Contig40

ATTGAGTTTGGAAACAGCTGAAATTCCAGAAAAATTGCTTTTTCAGGTCTCTCTGCTGCCGGAAATGCTCTCTGTTCAAAAAGCTTTTACACTCTTGACCAGCGCACTCCGTCACCATACCATAGCACTCTTTGAGTTTCCTCTAATCAGGTTCCACCAAACAGATACCCCGGTGTTTCACGGAATGGTACGTTTGATATCGCTGATTTGAGAGGAGGTTACACTTGAAGAATCACAGT

>Contig41

GGGTCGGGTAGTGAGGGCCTTGGTCAGACGCAGCGGGCGTGCTTGTGGACTGCTTGGTGGGGCTTGCTCTGCTAGGCGGACTACTTGCGTGCCTTGTTGTAGACGGCCTTGGTAGGTCTCTTGTAGACCGTCGCTTGCTACAATTAACGATCAACTTAGAACTGGTACGGACAAGGGGAATCTGACTGTCTAATTAAAACATAGCATTGCGATGGTCAGAAAGTGATGTTGACGCAAT

>Contig42

GGCAATCCCGGTTGGTTTCTTTTCCTCCGCTTATTGCTATGCTTAAG

>Contig43

GTAAAAAAAGAGCTGCGAATAGTAGCTTTCCGCCAATCAAACTCAAGAGCAGGACTAAGCTAGATAGTAACACAAGTGGCACAAACCTCTCGAGAATATGAATACCAACAGATCGGAAGAGCACACGTCTGAACCCAATCACTCCAATCGACCTCGTATGCCGTCTTCTGCTTGAAAAAAAAAAACAACAA

>Contig44

GTCTTTCGCCCCTATACCCAAATTCGACGATCGATTTGCACGTCAGAACCGCTACAGAACGGAAAGAGCACACGTCTGAACTCCCAGGCACTACAATCGATCTCGTATGCAGTCTTCTGCTTGAAAAAAAAAA

>Contig45

AGAGCAGCGCGTTGCCTACAGAAACATCTTTATAAAATCTTGGCCGGCCAAGATTCCCAAACGAGACTCACATTTCCCAAAAAAGACATTTCTGTCCAAAAGTAGAAGGCAAGAAAACCCTGGAGGAATCATAGGCAAAGAAAGAAAAGAAGAAGTTCATCTTTAAAACTACCTTTCAAGCCTTTATTCGTTCCTCGTAAAGGACACACGAAAAAAATAAACAGTACCTTGCAGAAGG

>Contig46

AACGGTTTAGTGTTTTCTTACCCAATTGTAGAGACTAACCACAAGGAC

>Contig47

AGTAACATAAATCACTAGTGAATTCGCGGCCGGATCGGAAGAGCACACGTCTGAACTCCCGTCACTCCAATCGATCTCGTATGCCATCTTCTGCTTGAAAAAAAAAAC

>Contig48

AGGGTCGGGTAGTGAGGGCCTTGGTCAGACGCAGCGGGCGTGCTTGTGGACTGCTTGGTGGGGCTTGCTCTGCTAGGCGGACTACTTGCGTGCCTTGTTGTAGACGGCCTTGGTAGGTCTCTTGTAGACCGTCGCTTGCTACAATTAACGATCAACTTAGAACTAGATCGGAAGAGCACACGTCTGAACCTCCAGTAACTCCAATCGAACTCGTATGCCGTCTTCTGCTTGAAAAAAA

>Contig49

TAACGGTTTAGTGTTTTCTTACCCAATTGTAGAGACTATCCACAAGGACAATATTTGTGACTTATGTTATGCGCCTGCTAGAGTTCCGGACTCCGTTCAACTTAAGGCGAAGTTGTTGGCAGAAAATGCAATCAAATCTTTTCCCGGTTGTGGTATATTTGGTGTGGAAATGTTCTATTTAGAAACAGGGGAATTGCTTATTAACGAAATTGCCCCAAGGCCTCACAACTCTGGACATTATACCATT

>Contig50

AAGTAAAAAAAGAGCTGCGAATAGTAGCTTTCCGCCAATCAAACTCAAGAGCAGGACTAAGCTAGATAGTAACACAAGTGGCACAAACCTCTCGAGAATATGAATACCAACGAGTCCGAACATGTTAGCACAAGCCCAGAGGATACTCAGGAGAACGGTGGAAACGCTAGCTCCAGCGGCAGTTTGCAGCAAATTTCCACGCTAAGAGAGCAGGACAGATGGCTACCCATCAACAATGT

>Contig51

AGTGGGTGGTAAATTCCATCTAAAGCTAAATATTGGCGAGAGACCGATAGCGAACAAGTACAGTGATGGAAAGATGAAAAGAACTTTGAAAAGAGAGTGAAAAAGTACGTGAAATTGTTGAAAGGGAAGGGCATTTGATCAGACATGGTGTTTTGTGCCCTCTGCTCCTTGTGGGTAGGGGAATCTCGCATTTCACTGGGCCAGCATCAGTTTTGGTGGCAGGATAAATCCATAGGAATGT

>Contig52

AACGGTTTAGTGTTTTCTTACCCAATTGTAGAGACTAAGATCGGAAGAGCACACGTCTGAACTCCAGTCACTCCAATCGATCTCGTATGCCGTCTTCTGCTTGAAAAAAAAAAAAAAAAA

>Contig53

ACGGTTTAGTGTTTTCTTACCCAATTGTAAGATCGGAAGAGCACACGGTCTGAACTCCAGTCACTCCAATCGATCTCGTATGCCGTCTTCTGCTTGAAAAAAAAAAAAAAAAA

>Contig54

AGGGTCGGGTAGTGAGGGCCTTGGTCAGACGCAGCGGGCGTAGATCGGAAGAGCACACGTCTGAACCCAGTCACTCCAATCGATCTCGTATGCCGTCTTCTGCTTGAAAAAAAAAAAAAAAAAAA

>Contig55

GTACTTATGTGCTTTATGAATGTGAATATTGTTACAGCTATGCAATGGTACGATCATACTCTGAACGATCCATTTAAATTATATATGTCAAACAGATCGGAAGAGCACACGTCTGAACTCCAGTCACTCCAATCGATCTCGTATGACGTCTTCTGCTTGAAAAAAAAAAAAAAA

**Haploid_37-48.contigs**

>Contig1

ACGGTTTAGTGTTTTCTTACCCAATTGTAGAGACTATCCACAAGGACAATATTTGTGACTTATGTTATGCGCCTGCTAGAGTTCCGGACTCCGTTCAACTTAAGGCGAAGAGATCGGAAGAGCACACGTCTGAACTCCATCACCTGCACTTATCTCGTATGCCGTCTTCTGCTTGAAAAAAAAAAAAAAACA

>Contig2

AACGGTTTAGTGTTTTCTTACCCAATTGTAGAGACTATCCACAAGGACAATATTTGTGACTTATGTTATGCGCCTGCTAGAGTTCCGGACTCCGTTCAACTTAAGGCGAAGTTGTTGGCAGAAAATGCAATCAAATCTTTTCCCGGTTGTGGTATATTTGGTGTGGAAATGTTCTATTTAGAAACAGGGGAATTGCTTATTAACGAAATTGCCCCAAGGCCTCACAACTCTGGACATTATACCATT

>Contig3

ACGGTTTAGTGTTTTCTTACCCAATTGTAGAGACTATCCACAAGGACAATATTTGTGACTTATAGATCGGAAGAAGATCGGAAGAGCACACGTTCTGAACTCCAGTCACCTGCACTTATCTCGTATGCCGTCTTCTGCTTGAAAAAAAAAAAAAAAAAAC

>Contig4

ACGGTTTAGTGTTTTCTTACCCAATTGTAGAGACTATCCACAAGGACAATATTTGTGACTTATGTTATGCGCCTGCTAGAGTTCCGGACTCCGTTCAACTTACGGCGAAGTTGTTGGCAGAAAATGCAATCAAATCTTTTCCCGGTTGTGGTATAGATCGGAAGAGCACACGTCTGAACACAAGCACCTGCACTTAACTCGTATGCCGTCTTCTGC

>Contig5

GTCTTCAATAAATCCAAGAATTTCACCTCTGACAATTGAATACTGATGCCCCCGACCGTCCCTATTAATCATTACGATGAGATCGGAAAAAGCACACGTCTGAACTCCAGTCACCTGCACTTATCTCGTATGCCGTCTTCTGCTTGAAAAAAAAAAAAACACAACAAA

>Contig6

ACTCCCCATTTCCTTATTGAACTTTGGGGATTCGGAGTTCCTTTTTTTTTCCGCCACTCCGATAACTAAACTTTGCAGTTTCACTGCTTGTTAAACATTTTCCTACTATAAATGACATTTAAAACGTAATTGAGACAAAAAAAGTTAAATCGTTATTAACTTTCACTGCGGCTCCGCTCTTTTCCTCATAAGGAAATCCCCTCTTATGATCTTGAGAAATGGGGGAATGTTGAGATAAT

>Contig7

AACGCGTCCTTGTACTGCGTCTAACGCTTTTGCCACTTGGATTTCTATTATAGGAAATAGTCTCACTTACTGGGCGACGAATTTTCGCGTTTTGATGAAGCACAGGAAGAATTTCTTTTTTTTTTGGCTTCTTCTGGTTCCGTTTTTTACGCGCACAAATCTAAAAAAAGAAATAATTATAACCTAGTCTCGAAAATTTTCATCGATCCATTCGTTCCTTTTTTTCGATTTTTTCATAT

>Contig8

ACGGTTTAGTGTTTTCTTAGATCGGAAAGAGCACACGTCTGAACTCCAGTCACCTGCACTTATCTCGTATGCCGTCTTCTGCTTGAAAAAAAAAAAAAA

>Contig9

TAGCAATCTATTTCAAAGAATTCAAACTTGGGGGAATGCCTTGTTGAATAGCCGGTCGCAAGACTGTGATTCTTCAAGTGTAACCTCCTCTCAAATCAGATCGGAAGAGCACACGTCTGAACTCCAGTCACCTGCACTTATCTCGTATGCCGTCTTCTGCTTGAAAAAAAAAAAACACACAA

>Contig10

AATGACCAAGTTTGTCCAAATTCTCCGCTCTGAGATGGAGTTGCCCCCTTCTCTAAGCAGATCCTGAGGCCTCACTAAGCCATTCAATCGGTACTAGCGACGGGCGGTGTGTACAAAGGGCAGGGACGTAATCAACGCAAGCTGATGACTTGCGCTTACTAGGAATTCCTCGTTGAAGAGCAATAATTACAATGCTCTATCCCCAGCACGACGGAGTTTCACAAGATTACCAAGACCAC

>Contig11

ACGGTTTAGTGTTTTCTTACCCAATTGTAGAGACTATCCACAAGGACAATATTTGTGACTTATGTTATGCGCCTGCTAGAGTTCCGGACTCCGTTCAACTTAAGGCGAAGTTGTTGGCAGAAAATGCAAAGATCGGAAAGAGCACACGTCTGAACTCCAGTCACCTGCACTTATCTCGTATGCCGTCTTATGCTTGAAAAAAAAAAAAAAA

>Contig12

ACGGTTTAGTGTTTTCTCACCCAATTGTAGAGACTATCCACAAGGACAATATTTGTGACTTATGTTATGCGCCTGCTAGATCGGAAGAGCACACGTCTGAATCCAGTCACCTGCACTTATCTCGTATGCCGTCTTCTGCTTGAAAAAAAAAAACA

>Contig13

ATAGTTTCTTTACTTATTCAATGAAGCGGAGCTGGAATTCATTTTCCACGTTCTAGCATTCAAGGTCCCATTCGGGGCTGATCCGGGTTGAAGACATTGTCAGGTGGGGAGTTTGGCTGGGGCGGCACATCTGTTAAACGATAACGCAGATGTCCTAAGGGGGGCTCATGGAGAACAGAAATCTCCAGTAGAACAAAAGGGTAAAAGCCCCCTTGATTTTGATTTTCAGTGTGAATACA

>Contig14

ATAGCAATCTATTTCAAAGAATTCAAACTTGGGGGAATGCCTTGTTGAATAGCCGGTCGCAAGACTGTGATTCTTCAAGTGTAACCTCCTCTCAAATCAGCGATATCAAACGTACCATTCCGTGAAACACCGGGGTATCTGTTTGGTGGAACCTGATTAGAGGAAACTCAAAGAGTGCTATGGTATGGTGACGGAGTGCGCTG

>Contig15

ATATTCACGTAGTTGCCAAAAGTAATTTTTGGAAAACTATTATTCCTCCGAGAAAACCTCACACAGAAATCCTTGCAGGTCTCATCTGGAATATAATTCCCCCCTCCTGAAGCAAATTTTTCCTTTGAGCCGGAATTTTTGATATTCCGAGTTCTTTTTTTCCATTCGCGGAGGTTATTCCATTCCTAAACGAGTGGCCACAATGAAACTTCAATTCATATCGACCGACTATTTTTCTCC

>Contig16

GTCTTCAATAAATCCAAGAATTTCACCTCTGACAATTGAATACTGATGCCCCCGACCGTCCCTATTAATCATTACGATGGTCCTAGAAACCAACAAAATAGAACCAAACGTCCTATTCTATTATAGATCGGAAGAGCACACGTCTGAACCTCCAGTCACCTGCACTTATCTCGTATGCCGTCTTCTGCTTGAAAAAAAAAAAAACAAACA

>Contig17

ACGGTTTAGTGTTTTCTTACACAATTGTAGAGAATATCCACAAGGACAATATTTGTGACTTATGTTATGCGCCTGCTAGAGTACCGGACTCCGAGATCGGAAGAGCACACGTCTGAACTCAAGTCACCTGCACTTATCTCGTATGCCGTCTTCTGCTTGAAAAAAAAAAAAATAAAAA

>Contig18

AGTCTTCAATAAATCCAAGAATTTCACCTCTGACAATTGAATACTGATGCCCCCGACCGTCCCTATTAATCATTACGATGGTCCTAGAAACCAACAAAATAGAACCAAACGTCCTATTCTATTATTCCATGCTAATATATTCGAGCAATACGCCTGCTTTGAACACTCTAATTTTTTCAAAGTAAAAGTCCTGGTTCGCCAAGAGCCACAAGGACTCAAGGTTAGCCAGAAGGAAAGGCC

>Contig19

ACGGTTTAGTGTTTTCTTACCCAATTGTAGAGACTATCCACAAGGACAATATTTGTGACTTATGTTATGCGCCTGCTAGAGTTCCGGACTCCGTTCAACTTAAGGCGAAGTTGTTGGCAGAAAATGCAATCAAATCTTTTCCCGGTTGTGGTATATTTGGTGTGGAAATGTTCTATTTAGAAACAGATCGGAAGAGCACACGTCTGAACCCAGTCACCTGCACTTAACTCGTATGCCGTCTTCTGCTT

>Contig20

ACGGTTTAGTGTTTTCTTACCCAATTGTAGAGACTATCCACAAGGACAATATTTGTGAGATCGGAAGAGCACACGTCTGAACTCAGTCACCTGCACTTATCTCGTATGCCGTCTTCTGCTTGAAAAAAAAAAAAAAAAAA

>Contig21

TAGCAAGAAAATTTCTTTTGTCAAACCTCCATTTAACCTTTGGTGGTTGATAAACATGAAATGGGCAATTTCACGAAAAGCTAAACTTTGTTTTCGTCTCCGCCTAGATGACTCTACCTTCAGGAGTAATATTTACACTATTTCTTCGCAGGCATTCGTTTATCGGCTTGGCAGGTCTAGATTTTTCTCTCCAATAAATTCTTTCCAAACATGTAAAGAAGATTTAGAATAAGAAATTTT

>Contig22

ATGACCAAGTTTGTCCAAATTCTCCGCTCTGAGATGGAGTTGCCCCCTTCTCTAAGCAGATCCTGAGGCCTCACTAAGCCATTCAAGATCGGAAGAGCACACGTCTGAACCCAGTCACCTGCACTTATCTCGTATGCCGTCTTCTGCTTGAAAAAAAAAAACAT

**1CopyDiploid_1-12.contigs**

>Contig1

CTACTGCGAAAGCATTTGCCAAGGACGTTTTCATTAATCAAGAACGAAAGTTAGGGGATCGAAGATGATCAGATACCGTCGTAGTCTTAACCATAAACTATGCCGACTAGGGATCGGGTGGTGTTTTTTTAATGACCCACTCGGCACCTTACGAGAAATCAAAGTCTTTGGGTTCTGGGGGGAGTATGGTCGCAAGGCTGAAACTTAAAGGAATTGACGGAAGGGCACCACCAGGAGTG

>Contig2

ACGGTTTAGTGTTTTACTTGAAGATTCTTTAGTGTAGGAACATCAACATGCTCAATCTCAATCGTTAGCACATCACATTTTTCAGCTAGTTTTTCGATATC

>Contig3

GGATCGACTAACCCACGTCCAACTGCTGTTGACGTGGAACCTTTCCCCACTTCAGTCTTCAAAGTTCTCATTTGAATATTTGCTACTACCACCAAGATCTGCACTAGAGGCCGTTCGACCCGACCTTACGGTCTAGGCTTCGTCACTGACCTCCACGCCTGCCTACTCGTCAGGGCATCATATCAACCCTGACGGTAGAGTATAGGTAACACGCTTGAGCGCCATCCATTTTCAGAGC

>Contig4

GCGGAAACAAAACGATCAAACCTTATACCATGAATATAATGGTGGATATTTATTACGGTAAGGAAACACTCTGAGCCAGGCTTGTAAATAGCGGTTATCTAAGCTTGTAACTAAAGAAATCAATTTGCAAGATCGGAAGAGCAACACGTCTGAACTCCAGTCACCGCTTAACATCTCGTATGCCGTCTTCTGCTTGAAAAAAAAAAAAAAAA

>Contig5

ACGGTTTAGTGTTTTCTTACCCAATTGTAGAGACTATCCACAAGGACAATATTTGTGACTTATGTTATGCGCCTGCTAGCGTTCCGGACTCCGTTCAACTTAAGGCGAAGTTGTTGGCAGAAAATGCAATCAAATCTTTTCCCGGTTGTAGATCGGAAGAGCACACGTCTGAACTCCAGTCACCGCTTAACATCTCGTATGCCGTCTTCTGCTTGAAAAAAAAAAAACAA

>Contig6

ACGGTTTAGTGTTAGATCGGAAAGAGCACACGTCTGAACTCCAGTCACCGCTTAACATCTCGTATGCCGTCTTCTGCTTGAAAAAAAAAAAAAAAAAA

>Contig7

ACGGTTTAGTGTTTTCTTACCCAATTGTAGAGATCGGAAGAGCACACGTTCTGAACTCCAGTCACCGCTTAACATCTCGTATGCCGTCTTCTGCTTGAAAAAAAAAAAAACAAAA

>Contig8

TTAGATAAAAAATCAATGTCTTCGGACTCTTTGATGATTCATAATAACTTTTCGAATCGCATGGCCTTGTGCTGGCGATGGTTCATTCAAATTTCTGCCCTATCAACTTTCGATGGTAGGCTAGTGGCCTACCATGGTTTCAACGGGTAACGGGGAGATCGGAAGAGCACAACGTCTGAACTCCAGTCACCGCTTAACAAATCGTATGCCGTCTTCTGCTT

>Contig9

TTACATCCTAATGCGGTCTATCGTTTCTGCAATATGAGAACCACATTCAGATCGGAAGAGCGTCGGAAGAGCACACGTCTGAACTCCAGTCACCGCTTAACATCTCGTATGCCGTCTTCTGCTTGAAAAAAAAAAAAAAAAAAAAA

>Contig10

ACAAAAGCACAGAAATCTCTCACCGTTTGGAATAGCAAGAAAGAAACTTACAAGCCTAGCAAGACCGCGCACTTAAGCGCAGGCCCGGCTGGACTCTCCATCTCTTGTCTTCTTGCCCAGTAAAAGCTCTCATGCTCTTGCCAAAACAAAAAAATCCATTTTCAAAATTATTAAATTTCTTTAATGATCCTTCCGCAGGTTCACCTACGGAAACCTTGTTACGACTTTTAGTTCCTCTAA

>Contig11

AGCAGTGGTAGTTTTAATGCGTTGGCCGGATTGTTTATATTTTGTGAAGCAATCCTGGAAATTTTAAGCATCAAAGAAGGTGCGGTGCCACCATAAGACTTCAAAAATAGTTGGAAGTTTTACTATCCTACATAAATGGTTTACTTAAACAAGGATGCCGAACCACTTGGATCTTGGTAAATAATTGTCCCTCAATTCTTGCCACTGGACAATGAATTCATCCACTACCCAATATAATATTT

>Contig12

ACGGTTTAGTGTTTTCTTACCCAATTGTAGAGACTATCCACAAGAGATCGGAAGAGCACACGTCTGAACTCCAGTCACCGCTTAACATCTCGTATGCCGTCTTCTGCTTGAAAAAAAAAAAAAAAAAAA

>Contig13

CTACCTTTATTTTATGTTTACTTTTTATAGATTGTCTTTTTATCCTACTCTTTCCCACTTGTCTCTCGCTACTGCCGTGCAACAAACACTAAATCAAAACAGTGAAATACTACTACATCAAAACGCATATTCCCTAGAAAAAAAAATTTCTTACAATATACTATACTACACAATAAATAATCACTGACTTTCGAAACAA

>Contig14

ACGGTTTAGTGTTTTCTTACCCAATTGTAGAGACTATCCACAAGGACAATATTTGTGACTTATGTTATGCGCCTGCTAGAGTTCCGGACTCCGTTCAACTTAAGGCGAAGTTGTTGGCAGAAGATCGGAAGAGCACACGTCTGAACTCCAGTCACCGCTTAACATCTCGTATGCCGTCTTCTGCTTGAAAAAAAAAAAAAACAAA

>Contig15

ACGGTTTAGTGTTTTCTTACCCAATTGTAGAGACTATCCACAAGGACAATATTTGTGACTTATGTAGATCGGAAGAGCACACGTCTGAATCCAGTCACCGCTTAACATCTCGTATGCCGTCTTCTGCTTGAAAAAAAAAAAAAAAAAT

>Contig16

TTACATCCTAATGCGGTCTATCGTTTCTGCAATATGAGAACCACATTCAGAACATTATCCTCATATGGCAAATAAACGAGATATCAAGATACCGGACCCCCTTTTTTACAGTAGATAAACCAGAGCGGAAGAGCAACACGTCTGAACTCCAGTCACCGCTTAACATCTCGTATGCCGTCTTCTGCTTGAAAAAAAAAAAAAAAA

>Contig17

AACGGTTTAGTGTTTTCTTACCCAATTGTAGAGACTATCCACAAGGACAATATTTGTGACTTATGTTATGCGCCTGCTAGAGTTCCGGACTCCGAGATCGGAAGAGCACACGTCTGAACTCCAGTCACCGCTTAACATCTCGTATGCCGTCTTCTGCTTGAAAAAAAAAACAAA

>Contig18

ACGGTTTAGTGTTTTCTTACCCAATTGTAGAGACTATCCACAAGGACAATAGATCGGAAGAGCACACTCTGAACTCCAGTCACCGCTTAACAACTCGTATGCCGTCTTCTGCTTGAAAAAAAAAAAAAACCA

>Contig19

GGCCTTATTAGATAAAAAATCAATGTCTTCGGACTCTTTGATGATTCATAATAACTTTTCGAATCGCATGGCCTTGTGCTGGCGATGGTTCATTCAAATTTCTGCCCTATCAACTTTCGATGGTAGGATAGTGGCCTACCATGGTTTCAACGGGTAACGGGGAATAAGGGTTCGATTCCGGAGAGGGAGCCTGAGAAACGGCTACCACATCCAAGGAAGGCAGCAGGCGCGCAAATTACCCAATCC

>Contig20

AGCGGAAACAAAACGATCAAACCTTATACCATGAATATAATGGTGGATATTTATTACGGTAAGGAAACACTCTGAGCCAGGCTTGTAAATAGCGGTTATCTAAGCTTGTAACTAAAGAAATCAATTTGCATCTTTCGTCCATGAGTGTCAGCCTTGAGCAAACGCTCGGATTCAGAATAAAAGTTACGAACGTGTTGGATGTAGTTACTGAAGGAAGATTGTATTCGTTCAATTCATCCAA

>Contig21

ATAAATTGCATCTGGATTGCGATTTTGGGTCATGCCTAACTGGGAAAATATTTACATCTCCATGTTCTCATTCTGCCTTTGCTTTCCTTCAAAACATGGTTTTCAATTCAACCTAGGGTCTACCGCGGAACCATATGTACGCCGTAAATGCGAGTGTTCTTGAAAGGTAGTAATGTGTTCCCTCTCAATATGTAAGTCCTTCAAGAGTATGCACTTGCAGTTGCACTGTTTCGGGTAAT

>Contig22

AGCACCACTCAGATGTGATATTTGACGTGTCGATTGTGATACCTGAAGACGCTATTGGAGAGCCATTGCAACGCACAGTCCAACAGTAATAAGCTCTGATCGTTTTGAAAAAGTATGTTTCTTTCCATTGGACGGTTGTCTTCCGATCAAAATTTTATCTCCTATACAATTTCTTCCTATTTACTAACGAAAACCTGCGAACCTGTAAACTACATGATTTTTAATATTAGTGAACTTCAA

>Contig23

AATTACAATGCTCTATCCCCAGCACGACGGAGTTTCACAAGATTACCAAGACCTCTCGGCCAAGGTTAGACTCGCTGGCTCCGTCAGTGTAGCGCGCGTGCGGCCCAGAACGTCTAAGGGCATCACAGACCTGTTATTGCCTCAAACTTCCATCGGCTTGAAACCGATAGTCCCTCTAAGAAGTGGATAACCAGCAAATGCTAGCACCACTATTTAGTAGGTTAAGGTCTCGTTCGTTAT

>Contig24

ATTACATCCTAATGCGGTCTATCGTTTCTGCAATATGAGAACCACATTCAGAACATTATCCTCATATGGCAAATAAACGAGATATCAAGATACCGGACCCCCTTTTTTACAGTAGATAAACCGGAGAATACCAGAGTATTTTCCAAATATAGAAAGAAAAGGGTTATTTCTAATTCTCGCTCTCTGTTTTTGGAAGACGAAGTTATTAAGTTTTTGCCTGCATTTGGAAACCTGCTGCG

>Contig25

TGGCCTTAACGGTTTAGTGTTTTCTTACCCAATTGTAGAGACTATCCACAAGGACAATATTTGTGACTTATGTTATGCGCCTGCTAGAGTTCCGGACTCCGTTCAACTTAAGGCGAAGTTGTTGGCAGAAAATGCAATCAAATCTTTTCCCGGTTGTGGTATATTTGGTGTGGAAATGTTCTATTTAGAAACAGGGGAATTGCTTATTAACGAAATTGCCCCAAGGCCTCACAACTCTGGACATTATACCATTGATGCTTG

**1CopyDiploid_13-24.contigs**

>Contig1

AATTGAACGTGGACATTTGAATGAAGAGCTTTTAGTGGGCCATTTTTGGTAAGCAGAACTGGCGATGCGGGATGAACCGAACGTAGAGTTAAGGTGCCGGAATACACGCTCATCAGACACCACAAAAGGTGTTAGTTCATCTAGACAGCCGGACGGTGGCCATGGAAGTCGGAATCCGCTAAGGAGTGTGTAACAACTCACCGGCCGAATGAACTAGCCCTGAAAATGGATGGCGCCC

>Contig2

CAGTTTTTCTATATGAGAAAAAAAAAAAAAAAAAAAAAAAGAAAAAGCAACAACACAA

>Contig3ATCAGCCCATATAAACACCGCCTATCCCCCGCCGAAAAACTAGCCGGATCTATCTAAAGCGCCAAAAATCATTTCGCCGGAAACAAACCGAGAAAAAAATGCCTTTCTCGAAAGTAAACAAAGCAGTACAACTGTTTTGGGTGTTATTTTGTTCCGTGGATTTTACGCCACTGTTTTGCTTTAAAAAAATTTCTCACACAATAGTCTAGTCTCAACGGCTGTGCATAGTACCCATAC

>Contig4

ACGGTTTAGTGTTTTCTTACCCAATTGTAGAGACTATCCACAGATCGGAAGAGCACACGTCTGAACTCCAGTCACCACCACTAATCTCGTATGCCGTCTTCTGCTTGAAAAAAAAAAAAAAAACA

>Contig5

GGGGGGCTCATGGAGAACAGAAATCTCCAGTAGAACAAAAGGGTAAAAGCCCCCTTGATTTTGATTTTCAGTGTGAATAGATCGAAGAGCACACGTCTGAACTCCAGTCACCACCACTAATCTCGTATGCCGTCTTCTGCTTGAAAAAAAAAAAAAAAAAAA

>Contig6

ACGGTTTAGTGTTTTCTTACCCAATTGTAGAGACTAGATCGGAAGAGCACACGTCTGAACTCCAGTCACCACCACTAATCTCGTATGCCGTCTTCTGCTTGAAAAAAAAAAAAAACAAATAAT

>Contig7

ACGGTTTAGTGTTTTCTTACCCAATTGTAGAGACTATCCACAAGGACAATATTTGTGACTTATGTTATGCGCCTGCTAGAGTTCCGGACTCCGTTCAACTTAAAGATCGGAAGAGCACACGTCTGAACTCCAGTCACCACCACTAATCTCGTATGCCGTCTTCTGCTTGAAAAAAAAAAAAAACAAACAA

>Contig8

ACGGTTTAGTGTTTTCTTACCCAATTGTAGAGACTATCCACAAGGACAATATAGATCGGAAGAGCACACGTCTGAACTCCAGTCACCACCACTAATCTCGTATGCCGTCTTCTGCTTGAAAAAAAAAAAAACAAAAA

>Contig9

ACGGTTTAGTGTTTTCTTACCCAATTGTAGAGATCGGAAGAGCACACGTCTGAACTCCAGTCACCACCACTAATCTCGTATGCCGTCTTCTGCTTGAAAAAAAAAAAAAAACCAC

>Contig10

TGGTTATATGCCGCCCGTCTTGAAACACGGACCAAGGAGTCTAACGTCTATGCGAGTGTTTGGGTGTAAAACCCATACGCGTAATGAAAGTGAACGTAGGTTGGGGCCTCGCAAGAGGTGCACAATCGACCGATCCTGATGTCTTCGGATGGATTTGAGTAAGAGCATAGCTGTTGGGACCCGAAAGATGGTGAACTATGCCTGAATAGGGTGAAGCCAGAGGAAACTCTGGTGGAGG

>Contig11

TAAACTGATGTATACTACTATTTTCCAAAAGAACCTGAAAAATTCAATACCTTCGTAAAACGTGACGACTTTCATAATCTCAGAAATTGATTTGAAGTAACAATGAAGAGCGCTTTAATTTAAAAGTTAGTGTAGAGATCGGAAGAGCACACGTCTGAACTCCAGTCACCACCACTAATCTCGTATGCCGTCTTCTGCTTGAAAAAAAAAAAAAAAAAAAAAAAA

>Contig12

CAGTTTTTCTATATGAGAAAAAAAAAAAAAAAAAAAAAAGAAAAAGCAACAGCACCATTA

>Contig13

ACAGTTTTTCTATATGAGAAAAAAAAAAAAAAAAAAAAAAAAAAAAAAAAAAAAAAAAAAAAA

>Contig14

ACGGTTTAGTGTTTTCTTACCCAATTGTAGAGACTATCCACAAGGACAATATTTGTGACTTATGTTATGCGCCTGCTAGATCGGAAGAGCACACGTCTGAACTCCAGTCACCACCACTAATCTCGTATGCCGTCTTCTGCTTGAAAAAAAAAAAAAAAAAACA

>Contig15

AATACACAGTAGAACGCAGACCCATTCGAGGGGCTCATTGGAAACACGTAGTCGACATTAGTTCTAGATAATCCGCTTGATGGGCCACATATGGTAATGGCTTCTCGAAGCAGATGTTACGAGCCGCCAGAACGAGGCGGTGGCATCTGCCTCGCGCTGTTTTCTAGCGGCAGAGAAAACCCGTAGATATGTTTCTAATGCGCGTGTGCGGGGAATTTCGTTGTCACGTTGTTTTGGTA

>Contig16

ACGGTTTAGTGTTTTCTTACCCAATTGTAGAGACTATCCACAAGGACAATATTTGTGACTTATGAGATCGGAAGAGCACACGTCTGAACTCCAGTCACCACCACTAATCTCGTATGCCGTCTTCTGCTTGAAAAAAAAAAAAAAAAA

>Contig17

AAGGGGGGCTCATGGAGAACAGAAATCTCCAGTAGAACAAAAGGGTAAAAGCCCCCTTGATTTTGATTTTCAGTGTGAATACAAACCATGAAAGTGTGGCCTATCGATCCTTTAGTCCCTCGGAATTTGAGGCTAGAGGTGCCAGAAAAGTTACCACAGGGATAACTGGCTTGTGGCAGTCAAGCGTTCATAGCGACATTGCTTTTTGATTCTTCGATGTCGGCTCTTCCTATCATACCGA

>Contig18

GGCAATCCCGGTTGGTTTCTTTTCCTCCGCTTATTGATATGCTTAAGTTCAGCGGGTACTCCTACCTGATTTGAGGTCAAACTTTAAGAACATTGTTCGCCTAGACGCTCTCTTCTTATCGATAACAGATCGGAAGGAGCACACGTCTGAACTCCAGTCACCACCACTAATCTCGTATGCCGTCTTCTGCTTGAAAAAAAAAAA

>Contig19

ACGGTTTAGTGTTTTCTTACCCAATTGTAGAGACTATCCACAAGGAGATCGGAAGAGCACACGTCTGAACTCCAGTCACCACCACTAATCTCGTATGCCGTCTTCTGCTTGAAAAAAAAAAAAAAAAAC

>Contig20

CAGTTTTTCTATATGAGAAAAAAAAAAAAAAAAAAAAAAAAAAAAAACAACAACAC

>Contig21

ACGGTTTAGTGTTTTCTTACCCAATTGTAGAGACTATCCACAAGGACAATATTTGTGACTTATGTTATGCGCCTGCTAGAGTTCCGGACTCCGTTCAACTTAAGGCGAAGTTGTTGGCAGAAAATGCAATCAAATCTTTTCCCGGTTGAGATCGGAAGAGCACACGTCTGAACTCCAGTCACCACCACTAATCTCGTATGCCGTCTTCTGCTTGAAAAAAAAAAAAAAAAAAAA

>Contig22

ACGGTTTAGTGTTTTCTTACCCAATTGTAGATCGGAAGAGCACCACGTCTGAACTCCAGTCACCACCACTAATCTCGTATGCCGTCTTCTGCTTGAAAAAAAAAAAAAACAAA

>Contig23

ACGGTTTAGTGTTTTCTTACCCAATTGTAGAGACTATCCACAAGGACAATATTTGTGACTTATGTTATGCGCCTGCTAGAGTTCCGGACTCCGTAGATCGGAAGAGCACACGTCTGAACTCCAGTCACCACCACTAATCTCGTATGCCGGCTTCTGCTTGAAAAAAAAAAAAAAACTAAAAAA

>Contig24

CGAAGTCGTTCGAACCCATCAGATTTCTGCTTTGATTACATACACAAGCTTTGTTTCTTTCTACAGGTGATTGTATGTGGGCTTATGGTATGCCTGCAGGACGCGTATGCATGTACGACGGCACGACCCGGAGCAAAACGGCGAGTGCTCTAACGCGGTGTATAGATCGGAAGAGCACACGTCTGAACCCAGTCACCACCACTAATCTCGTATGCCGTCTTCTGCTTGAAAAAAAAAAAAAACAAA

>Contig25

AACGCGAATGCTCTAGTGATGGCACAATAGAATAGTGTCTTAGCACCACAGAGCGTGATTTTTTAAAGCTCTATATTAGCAACTACGATAGGTGTTTATTTAGTTTCTTTGTTGATCTTCCTGTTTCCAGTGTTACGGCCTTGTCAGGTCAAAAATTTTACCCGGATCTCTAGTAATGAGTAACTTAAGAATATCCCATCTGATTATTGGTAGTTTTACTGAACCAAGTTAGAGCTAAAT

>Contig26

AGGCAATCCCGGTTGGTTTCTTTTCCTCCGCTTATTGATATGCTTAAGTTCAGCGGGTACTCCTACCTGATTTGAGGTCAAACTTTAAGAACATTGTTCGCCTAGACGCTCTCTTCTTATCGATAACGTTCCAATACGCTCAGTATAAAAAAAGATTAGCCGCAGTTGGTAAAACCTAAAACGACCGTACTTGCATTATACCTCAAGCACGCAGAGAAACCTCTCTTTGGAAAAAAA

>Contig27

CAGTTTTTCTATATGAGAAAAAAAAAAAAAAAAAAAAAAAAGAAAAAGCA

>Contig28

ACTGGCCTTAACGGTTTAGTGTTTTCTTACCCAATTGTAGAGACTATCCACAAGGACAATATTTGTGACTTATGTTATGCGCCTGCTAGAGTTCCGGACTCCGTTCAACTTAAGGCGAAGTTGTTGGCAGAAAATGCAATCAAATCTTTTCCCGGTTGTGGTATATTTGGTGTGGAAATGTTCTATTTAGAAACAGGGGAATTGCTTATTAACGAAATTGCCCCAAGGCCTCACAACTCTGGACATTATACCATTGA

>Contig29

ATAGGACAATTAAAACCGTTTCAATACAACACACTGTGGAGTTTTCATATCTTTGCAACTTTTTCTTTGGGCATTCGAGCAATCGGGGCCCAGAGGTAACAAACACAAACAATTTTATCTATTCATTAAATTTTTGTCAAAAACAAGAATTTTCGTAACTGGAAATTTTAAAATATTAAAAACTTTCAACAACGGATCTCTTGGTTCTCGCATCGATGAAGAACGCAGCGAAATGCGA

>Contig30

AACGAAGTCGTTCGAACCCATCAGATTTCTGCTTTGATTACATACACAAGCTTTGTTTCTTTCTACAGGTGATTGTATGTGGGCTTATGGTATGCCTGCAGGACGCGTATGCATGTACGACGGCACGACCCGGAGCAAAACGGCGAGTGCTCTAACGCGGTGTATGGGCTAGATTCGTAATAGGGCTCTGGGTTTGCTGCAAGGGGTAATTATCAGCGATGGAATGACTCCGTTACCGCCCG

>Contig31

CGAAGTCGTTCGAACCCATCAGATTTCTGCTTTGATTACATACACAAGCTTTGTTTCTTTCTACAGGTGATTGTAGATCGGAAGAGCACACGTCTGAACTCCAGTCACCACCACTAATCTCGTATGCCGTCTTCTGCTTGAAAAAAAAAAAAAAAACAAA

>Contig32

CCTAATAAACTGATGTATACTACTATTTTCCAAAAGAACCTGAAAAATTCAATACCTTCGTAAAACGTGACGACTTTCATAATCTCAGAAATTGATTTGAAGTAACAATGAAGAGCGCTTTAATTTAAAAGTTAGTGTAGTGGTGTTATAGAAGAATATTACACCTGCTAATATCATTCATTCGTGAAATATAAGCACCTTCTTTCTTTCCAATTTTGGTCTCCTCAAAAAGCAGGTTAATG

>Contig33

ACGCGAATGCTCTAGTGATGGCACAATAGAATAGTGTCTTAGCACCACAGATCGGAAGAGCACACGTCTGAACTCCATCACCACCACTAATCTCGTATGCCGTCTTCTGCTTGAAAAAAAAAAAAAAAACA

>Contig34

CAGTTTTTCTATATGAGAAAAAAAAAAAAAAAAAAAAAAAAAAGCAACCACAC

>Contig35

ACGGTTTAGTGTTTTCTTACCCAATTGTAGAGACTATCCACAAGGACAATATTTGTGACTTATGTTATGCGCCTGCTAGAGTTCCGGACTCCGTTCAACTTAAGGCGAAGTTGTTGGGAGATCGGAAGAGCACACGTCTGAACTCCAGTCACCACCACTAATCTCGTATGCCGTCTTCTGCTTGAAAAAAAAAAAAAAAAAA

>Contig36

ACGCGAATGCTCTAGTGATGGCACAATAGAATAGTGTCTTAGCACCACAGAGCGGGATTTTTTAAAGCTCTATATTAGCAACTAGATCGGAAGAGCCACGTCTGAACTCCAGTCACAACCACTAATCTCGTATGCCGTCTTCTGCTTGAAAAAAAAAAAAAA

>Contig37

TAAACTGATGTATACTACTATTTTCCAAAAGAACCTGAAAAATTCAATACCTTCGTAAAACGTGACGACTTTCATAATCTCAGAAATTGATTTGAAGTAACAATGAAGAGATCGGAAGAGCACACGTCTGAACCCAGTCACCACCACTAATCTCGTATGCCGTCTTCTGCTTGAAAAAAAAAAAAAAACA

>Contig38

AACGGTTTAGTGTTTTCTTACCCAATTGTAGAGACTATCCACAAGGACAATATTTGTGACTTATGTTATGCGCCTGCTAGAGTTCCGGACTCCGTTCAACTTAAGGCGAAGTTGTTGGCAGAAAATGCAATCAAATCTTTTCCCGGTTGTGGTATATTTGGTGTGAGATCGGAAAGGAGCACACGTCTGAACTCCAGTCACCACCACTAATCTCGTATGCCGTCTTCTGCTTGAAAAAAAA

**1CopyDiploid_25-36.contigs**

>Contig1

GCTAGTAATCCACCAAATCCTTCGCTGCTCACCAATGGAATCGCAAGATGCCCACGATGAGACTGTTCAGGTTAAACGCAAAAGAAACACACTCTGGGAATTTCTTCCCAAATTGTATCTCTCAATACGCATCAACCCATGTCAATTAAACACGCTGTATAGAGACTAGGCAGATCTGACGATCACCTAGCGACTCTCTCCACCGTTTGACGAGGCCATTTACAAAAACATAACGAA

>Contig2

AGTAACTGAATGAAAAAGTGAAAATGGAGGAAAAAAAAAAAGACGAAATCCAAAAGGCTAAGGAAACGGGTAACCCGTGGTACCCGGGGCAGAATCAAACTTATAATTACTAAAGATATACCATATATTACGATCCCTGCGCCCGGCGTGCTCCACATTAAGTAAATGTCTGGAGTAAAATGAAGTCCGCCAATTAAGTCACAAAACAAAAACGAAA

>Contig3

GTAACTGAATGAAAAAGTGAAAATGGAGGAAAAAAAAAAAGAAGAAAACCAACAGGCTAAGAAAACGGGTAACACG

>Contig4

GAAATCTCCCGACCTAGAAACTATCAAATCTTGATCTAATCACTAGTGAATTCGCGGCC

>Contig5

ACGGTTTAGTGTTTTCTTACCCAATTGTAGAGACTATCCACAAGGACAATATTTGTGACTTATGTTATGCGCCTGCTAGAGATCGGAAGAGCACACGTCTGAACTCCATCACACAGCAACATCTCGTATGCCGTCTTCTGCTTGAAAAAAAAAAAAAAAAAAAATA

>Contig6

ACTAGTATTTACATTACTAGTATATTATCATATACGGTGTTAGAAGATGACGCAAATGATGAGAAATAGTCATCTAAATTAGTGGAAGCTGAAACGCAAGGATTGATAATGTAATAGGATCAATGAATATAAACATATAAAACGGAATGAGGAATAATCGTAATATTAGTATGTAGAAATATAGATTCCATTTTGAGGATTCCTATATCCTCGAGGAGAACTTCTAGTATATTCTGTAT

>Contig7

GAACGCTGCCTCCAAATGTCTGGTGAACGTTCGTGAATATTCAGAGTGTCGTTATTGCAATGGAATGGCTGCCTTTGTGACGGCGTCAGGATAAATTGTGAGTCACTGAAACATATTCGGCAAAGAGATCGGAAGAGCACACGTCCTGAACTCCAGTCACACAGCAACATCTCGTATGCCGTCTTCTGCTTGAAAAAAAAAAAAAAAAAA

>Contig8

ATGAATCATCAAAGAGTCCGAAGACATTGATTTTTTATCTAATAAATACATCTCTTCCAAAGGGTCGAGATTTTAAGCATGTATTAGCTCTAGAATTACCACAGTTATACCATGTAGTAAAGGAACTATCAAATAAACGATAACTGATTTAATGAGCCATTCGCAGTTTCACTGTATAAATTGCTTATACTTAGACATGCATGGCTTAATCTTTGAGACAAGCATATGACTACTGGCAGG

>Contig9

ACGGTTTAGTGTTTTCTTACCCAATTGAAAACCGAAAGAGCACACGTCTGAACTCCAGTCACACAGCAACATCTCGTATGCCGTCTTCTGCTTGAAAAAAAAAAAAAAAACA

>Contig10

ACGGTTTAGTGTTTTCTTACCCAATTGTAGAGACTATCCACAAGGACAATATTTGTGACTTATGTTATGCGCCTGCTAGAGTTCCGGACTCCGTTCAGATCGGAAGAGCACACGTCTGAACTCAGTCACACAGCAACATCTCGTATGCCGTCTTCTGCTTGAAAAAAAAAAAAAAACAAAAC

>Contig11

GTAACTGAATGAAAAAGTGAAAATGGAGGAAAAAAAAAAAAAGACGAAATCCAAAAGGCAAAGGAAAC

>Contig12

AGTAATTCACTTTCTGATCCCGCACTCATAGCGATGGAATAATATACCGGATTTCACACCTTGTTATTGAGTGAAGTACTGCTTGGTGAAATGATATCTTTATGTTCAATATTAATGGTCGTGTGGATGAATATATGGGCATGGGTTAATTAGTTTTAGGGGCACGGAGTAAACAAGAAAGGAGGGCCAGAATCATTAGTAGAGTACCTCAAGTTTGGTTTCTTTTTGATTTCAGAACG

>Contig13

TGAATCATCAAAGAGTCCGAAGACATTGATTTTTTATCTAATAAATACATCTCTTCCAAAGGGTCGAGATTTTAAGCATGTATTAGCTCTAGAATTACCACAGTTATATATGGAAAGAGCACACGTCTGAACTCCAGTCACACAGCAACATCTCGTATGCCGTCTTCTGCTTGAAAAAAAAAAAAAAAAA

>Contig14

AGGTATTTACATTGTACTCATTCCAATTACAAGACCCGAATGGGCCCTGTATCGTTATTTATTGTCACTACCTCCCAGATCGGAAGAGCACACGTCTGAACTCCAGTCACACAGCAACATCTCGTATGCCGTCTTCTGCTTGAAAAAAAAAAAAAAAACACAAC

>Contig15

GCACAAATTTCATTTTTTTTTTTTTTCATTTTTCTTTAGACTTTAGCCCTAATTAGACCTGAGAAATATTATTATAACCTGAGAAAGGTTTATAAAAAGAACATTATAACCTACAAAAAAGATACTTAAACCCTGAAGTTAATAGGTTTGGACCCTCTTTTTTTTTTTTTTTTTTTTTTTAAAAACCTCAGTATTCAC

>Contig16

GCTAGTAATCCACCAAATCCTTCGCTGCTCACCAATGGAATCGCAAGATGCCCACGATGAGACTGTTCAGGTTAAACGAGATCGGAAAGAGCACACGTCTGAACTCCAGTCACACAGCAACAGCTCGTATGCCGTCTTCTGCTTGAAAAAAAAAAAAAACACAAAA

>Contig17

ATGATCCTTCCGCAGGTTCACCTACGGAAACCTTGTTACGACTTTTAGTTCCTCTAAATGACCAAGTTTGTCCAAATTCTCCGCTCTGAGATGGAGTTGCCCCCTTCTCTAAGCAGATCCTGAGGCCTCACTAAGCCATTCAATCGGTACTAGCGACGGGCGGTGTGTACAAAGGGCAGGGACGTAATCAACGCAAGCTGATGACTTGCGCTTACTAGGAATTCCTCGTTGAAGAGCA

>Contig18

AGTAACTGAATGAAAAAGTGAAAATGGAGGAAAAAAAAAAAGACGAAATCCAAAAGGCTAAGGAAACGAGATCGGAAAAGCACACG

>Contig19

AAGGTATTTACATTGTACTCATTCCAATTACAAGACCCGAATGGGCCCTGTATCGTTATTTATTGTCACTACCTCCCTGAATTAGGATTGGGTAATTTGCGCGCCTGCTGCCTTCCTTGGATGTGGTAGCCGTTTCTCAGGCTCCCTCTCCGGAATCGAACCCTTATTCCCCGTTACCCGTTGAAACCATGGTAGGCCAGATCGGAAGAG

>Contig20

GAATGCCTAAATTAGCTTAAACAAAAGTCATTTCTAGAAAGCAATGAAAAAAGGGCAATCTAATGATACCACCACGAATCGTTCCATAGATCGGAAGAGCACACGTCTGAACTCCAGTCACACAGCAACATCTCGTATGCCGTCTTCTGCTTGAAAAAAAAAAAAAAACAAC

>Contig21

TGATCCTTCCGCAGGTTCACCTACGGAAACCTTGTTACGACTTTTAGTTCCTCTAAATGACCAAGTTTGTCCAAATTCTCCGCTCTGAGATGGAGTTGCCCCCTTCTCTAAGCAGATCGGAAGAGCACACGTCTGAACCCAGTCACACAGCAACATCTCGTATGCCGTCTTCTGCTGGAAAAAAAAAA

>Contig22

ACTCTCCTTTGTCTTCGTTAATGAGTACTGGGGTGAAAAAAAAGGGTTATAATAAATAAATTATCCAAGAACGACTATTCTCTTTTCTTTTTTCTGCTTGCTCTTCCCCACTTTTCTCGTCCAGCCTGTTGTAAAAATTTGTTGTTGCTTCTTTCGTATTAGTTTTTCCGAAAGAAAGCGCCGCAGGGCCTTTACCATCACTCCAGGTTTTCCCGGAGTGGGGTGGCAGTCCGCAGTC

>Contig23

GAATGCCTAAATTAGCTTAAACAAAAGTCATTTCTAGAAAGCAATGAAAAAAGGGCAATCTAATGAGATCGGAAGAGCACACGTCTGAACTCCAGCACACAGCAACATCTCGTATGCCGTCTTCTGCTTGAAAAAAAAAAAAAAAACAACAA

>Contig24

CGGTTTAGTGTTTTCTTACCCAATTGTAGAGACTATCCACAAGGACAATATTTGTGACTTATGTTATGCGCCTGCTAGAGTTCCAGATCGGAAGAGCAACGTCTGAACTCCAGTCACACAGCAACATCTCGTATGCCGTCTTCTGCTTGAAAAAAAAAAAAACAGAGA

>Contig25

ACGGTTTAGTGTTTTCTTACCCAATTGAGATCGGAAGAGCACAACGTCTGAACTCCAGTCACACAGCAACATCTCGTATGCCGTCTTCTGCTTGAAAAAAAAAACAAAAACA

>Contig26

CTAGTATTTACATTACTAGTATATTATCATATACGGTGTTAGAAGATGACGCAAATGATGAGAAATAGTCATCTAAATTAGTGGAAGCTGAAACGCAAGGATTGATAATGTAATAGGATCAATGAATATAAACAAGATCGGAAGAGCACACCTCAAAACTCAGTCACACAGCAACAACTCGTATGACGTCTTCTGCTTGAAAAAAAAAAAAAAAAA

>Contig27

TAGAACGCTGCCTCCAAATGTCTGGTGAACGTTCGTGAATATTCAGAGTGTCGTTATTGCAATGGAATGGCTGCCTTTGTGACGGCGTCAGGATAAATTGTGAGTCACTGAAACATATTCGGCAAAGTAAAAAAACGGACGTAAAAGATTCTTTCTTTATTCTTTTCACGACAGTCTACAAGTAACTGAAATAGCATACATTTACCAAAAAAATCGAAAAAAATGTTTAATACTACACCAA

>Contig28

ACGGTTTAGTGTTTTCTTACCCAATTGTAGAGACTAACAGATCGGAAGAGCACACGTCTGAACCCAGTCACACAGCAACATCTCGTATGCCGTCTTCTGCTTGAAAAAAAAAAAAAAAAACCA

>Contig29

ACGGTTTAGTGTTTTCTTACCCAATTGTAGAGACTATCAGATCGGAAGAGCACACTGAACTCCAGTCACACAGCAACATCTCGTATGCCGTCTTCTGCTTGAAAAAAAAAAAAAAACAA

>Contig30

AAGAATGCCTAAATTAGCTTAAACAAAAGTCATTTCTAGAAAGCAATGAAAAAAGGGCAATCTAATGATACCACCACGAATCGTTCCATGGAGGGATTTTGCTGAACTAGAGGAGCTGAAACTTTGGTTTTACCCGAAAAGCAAAGGAACCATTGAAGATAAGCGACAGCGTGCAGTGCAAAGAGTACAGAGCTACCGACTAAAGGGTTCCCAATATTTACCTCACGTGGTGGACTCTACAGCACAAATAACA

>Contig31

TAACGGTTTAGTGTTTTCTTACCCAATTGTAGAGACTATCCACAAGGACAATATTTGTGACTTATGTTATGCGCCTGCTAGAGTTCCGGACTCCGTTCAACTTAAGGCGAAGTTGTTGGCAGAAAATGCAATCAAATCTTTTCCCGGTTGTGGTATATTTGGTGTGGAAATGTTCTATTTAGAAACAGGGGAATTGCTTATTAACGAAATTGCCCCAAGGCCTCACAACTCTGGACATTATACCATTA

**1CopyDiploid_37-48.contigs**

>Contig1

TAGGATGTATACGTAATACGTAATAGGTATCACGCTATTTACTCTTGCCTTTTATTTGTTTAATAGTTCGAATCACATGAGTAGAGGACGGAAATTGCTCTTCTATGGCGTTCAGTAGATCGGAAGAGGCACACGTCTGAACTCCAGTCACGGAAGGATAACTCGTATGCCGTCTTCTGCTTGAAAAAAAAAACAACAC

>Contig2

ACGGTTTAGTGTTTTCTTACCCAATTGTAGAGACTATCCAGATCGGAAGAGCACACGTCTGACTCCAGTCACGGAAGGATATCTCGTATGCCGTCTTCTGCTTGAAAAAAAAAAAAAAAA

>Contig3

ACGGTTTAGTGTTTTCTTACCCAATTGTAGAGACTATCCACCAGGACA

>Contig4

TTCTACACCCTCTATGTCTCTTCACAATGTCAAACTAGAGTCAAGCTCAACAGGGTCTTCTTTCCCCGCTGATTCTGCCAAGCCCGTTCCCTTGGCTGTGGTTTCGCTAGATAGTAGATAGGGACAGTGGGAATCTCGTTAATCCATTCATGCGCGTCACTAATTAGATGACGAGGCATTTGGCTACCTTAAGAGAGTCATAGTTACTCCCGCCGTTTACCCGCGCTTGGTTGAATTTT

>Contig5

TGCGATTAGTTTTTTAGCCTTATTTCTGGGGTAATTAATCAGCGAAGCGATGATTTTTGATCTATTAACAGATATATAAATGCAAAAACTGCATAACCACTTTAACTAATACTTTCAACATTTTCGGTTTGTATTACTTCTTATTCAAATGTAATAAAAGTATCAACAAAAAATTGTTAATATACCTCTATACTTTAACGTCAAGGAGAAAAAACCCCGGATTCTAGAACTAGTGGA

>Contig6

GAGGAACTAAAAGTCGTAACAAGGTTTCCGTAGGTGAACATGCGGAA

>Contig7

ACGGTTTAGTGTTTTCTTACCCAATTGTAGAGACTATCCAAGATCGGAAGGACACACGTCTGAACTCCAGTCACGGAAGGATATCTCGTATGCCGTCTTCTGCTTGAAAAAAAAAAAAAAAAAAA

>Contig8

ATAGGTATTTACTTTCACAAGTACGTGTACGCAATTCTAAAGGAAGTATATCAAATAAAAGGGCGTGGTACATAAAAGCTATATCCCTAGCATGACCAGTCTAATAGATTTGGGCAGATATGTTGAAAGAACGCATCATGGAGAAGATACAGAGCCAAGATCGAAAAGGGTAAAAATCGCAAAACCTGACTTGTCTTCCTTCCAACCAGGCAGCATTATTAAGATCCGTTTACAGGATT

>Contig9

ACGGTTTAGTGTTTTCTTACCCAATTGTAGAGACTATCCACAAGGACAATATTTGTGACTTATGTTATAGATCGGAAGAGCACACGTCTGAACCCAGTCACGGAAGGATATCTCGTATGCCGTCTTCTGCTTGAAAAAAAAAATATAA

>Contig10

GTGAAGAATTATGGACGTCCGGATGGTGTGCAGAAGCTTACAAACTAACTTCCTGGTGGAGAATAATGGTAGGCTTGGCGGCTCTGAGATGGAGAAATAGTTTCAAGCCGCGCGCTCAATAACTTCCTCGAGATCGGAAGAGCACACGTCTGAACTCCGTCACGGAAGGATATCTCGTATGCCGTCTTCTGCTTGAAAAAAAAAACAAAAA

>Contig11

TAACGGTTTAGTGTTTTCTTACCCAATTGTAGAGACTATCCACAAGGACAATATTTGTGACTTATGTTATGCGCCTGCTAGAGTTCCGGACTCCGTTCAACTTAAGGCGAAGTTGTTGGCAGAAAATGCAATCAAATCTTTTCCCGGTTGTGGTATATTTGGTGTGGAAATGTTCTATTTAGAAACAGGGGAATTGCTTATTAACGAAATTGCCCCAAGGCCTCACAACTCTGGACATTATACCATTGATGCT

>Contig12

ACGGTTTAGTGTTTTCTTACCCAATTGTAGAGACTATCCACAAGGACAATATTTGTGACTTATGTTATGCGCCTGCTAGAGTTCCGGAGATCGGAAGAGCACACCGTCTAAACTCCAGTCACGGAAGGATATCTCGTATGCCGTCTTCTGCTTGAAAAAAAAAAAAAAAAACAG

>Contig13

ACGGTTTAGTGTTTTCTTACCCAATTGTAGAGACTATCCACAAGGACAGATCGGAAGAGCACACGTCTGAACTCCAGTCACGGAAGGATATCTCGTATGCCGTCTTCTGCTTGAAAAAAAAAAAAAACA

>Contig14

AGAGGAACTAAAAGTCGTAACAAGGTTTCCGTAGGTGAACCTGCGGAAGGATCATTAAAGAAATTTAATAATTTTGAAAATGGATTTTTTTGTTTTGGCAAGAGCATGAGAGCTTTTACTGGGCAAGAAGACAAGAGATGGAGAGTCCAGCCGGGCCTGCGCTTAAGTGCGCGGTCTTGCTAGGCTTGTAAGTTTCTTTCTTGCTATTCCAAACGGTGAGAGATTTCTGTGCTTTTGTTAT

>Contig15

TAGGATGTATACGTAATACGTAATAGGTATCACGCTATTTACTCTTGCCTTTTCTTTGTTTCATAGTTCGAATCAGATCGGAAGAGCACACGTCTGAACTCCAGTCACGGAAGGATATCTCGTATGCCGTCTTCTGCTTGAAAAAAAAAAAAAAA

>Contig16

TAAAAAATTAATTAGAGCCTGTAACATAGAGATCAACGCTACTGTAGCGTTAGAAGAGATCGGAAGAGCACACGTCTGAACTCCATCACGGAAGGATATCTCGTATGCCGTCTTCTGCTTGAAAAAAAAAAAACAAAAAGA

>Contig17

ACGGTTTAGTGTTTTCTTAGATCGGAAGAGCACACGTCTGAACTCCAGTCACGGAAGGATATCTCGTATGCCGTCTTCTGCTTGAAAAAAAAAAAAAAAAAACA

>Contig18

ACGGTTTAGTGTTTTCTTACCCAATTGTAGAGATCGGAAGAGCACACGTCTGAACTCAGTCACGGAAGGATATCTCGTATGCCGTCTTCTGCTTGAAAAAAAAAAAAAAAAAAACA

>Contig19

ACGGTTTAGTGTTTTCTTACCCAATTGTAGAGACTATCCACAAGGACAATATTTGTGACTTATGTTATGCGCCTGCTAGAGTTCCGGACTCCGTTCAACTTAAGGCGAAGTTGTTGGCAGAAAATGCAATCAAATCTTTTCCCGGTTGTGGTATATTTGGTGTGGAAATGAGATCGGAAGAGCACACGTCTGAACTCCAGTCACGGAAGGATATCTCGTATGCCGTCTTCTGCTTGAAAAAAAAAAAAAAAAAACGTC

>Contig20

TAAAAAATTAATTAGAGCCTGTAACATAGAGATCAACGCTACTGTAGCGTTAGAAGGACAGTGAGAGAGTGGCATGACAATTAAAGTTTCTTCCGATTACAGATCGGAAGAGCACACGTCTGAACTCCATCACGGAAGGATATCTCGTATGCCGTCTTCTGCTTGAAAAAAAAAAAAAAAAAA

>Contig21

AAGTGAAGAATTATGGACGTCCGGATGGTGTGCAGAAGCTTACAAACTAACTTCCTGGTGGAGAATAATGGTAGGCTTGGCGGCTCTGAGATGGAGAAATAGTTTCAAGCCGCGCGCTCAATAACTTCCTCGATCACTGACTTTCGCACCCATCTGGGAACGGTACGGCGAATAGTCTTCATTTAATTGGAGCTGATCGAAAATGCTTGCTGCCGCTTCAGCGTGAATGACGCAGGTGCTGCGTTTGTCCT

>Contig22

ACGGTTTAGTGTTTTCTTACCCAATTGTAGAGACTATCCACAAGGACAATATTTGTGACTTATGTTATGCGCCTGCTAGAGTTCCGGACTCCGTTCAACTTAAGGCGAAGTTGTTGGCAGAAAATGCAATCAAATCTTTTCCCGGTTGTGGTATATTTGGTGTGGAAATGTTCTATTTAGAAACAGAGATCGGAAGAGCACACGCCGAAACCCAGTCACGGAAGGATATCTCGTATGCC

>Contig23

GAGGAACTAAAAGTCGTAACAAGGTTTCCGTAGGTGAACCTGCGGAAAGATCGGAGAGCACACGTCTGAACTCCAGTCACGGAAGGATATCTCGTATGCCGTCTTCTGCTTGAAAAAAAAAAAAAAAAA

>Contig24

ACGGTTTAGTGTTTTCTTACCCAATTGTAGAGACTATCCACAAGGACAATATTTGTGACTAGATCGGAAGAGCAACACGTCTGAACTCCAGTCACGGAAGGATATCTCGTATGCCGTCTTCTGCTTGAAAAAAAAAACAAA

>Contig25

ACGGTTTAGTGTTTTCTTACCCAATTGTAGAGATCGGAAAGAGCACACGTCTGAACTCCAGTCACGGAAGGATATCTCGTATGCCGTCTTCTGCTTGAAAAAAAAAAAAAAT

>Contig26

GTGAAGAATTATGGACGTCCGGATGGTGTGCAGAAGCTTACAAACTAACTTCCTGGTGGAGAATAATGGTAGGCTTGGCGGCTCTGAGATGGAGAAATAGTTTCAAGCCGCGCGAGATCGGAAAGCCCACGTCTGAACTCCAGTCACGGAAGGATATCTCGTATGCCGTCTTCTGCTTGAAAAAAAAAAAAAAAAAA

>Contig27

TAGGATGTATACGTAATACGTAATAGGTATCACGCTATTTACTCTTGCCTTTTCAGATCGGAAGAAGCACACGTCTGAACTCCAGTCACGGAAGGATATCTCGTATGCCGTCTTCTGCTTGAAAAAAAAAAAAAAA

>Contig28

ATAAAAAATTAATTAGAGCCTGTAACATAGAGATCAACGCTACTGTAGCGTTAGAAGGACAGTGAGAGAGTGGCATGACAATTAAAGTTTCTTCCGATTACTTCATGTCATCTGCATTACAAAGGAAGGAAAGATGTTTCGAATTATTTCCCCTTTAAGGTATTTTTGATGTGAAATTTGACCCCATACTGTGAAAATCAACTGCTAAGAACTCTGTGATCTTCTAAGATAAAAAGGCGG

>Contig29

TACTTAGACATGCATGGCTTAATCTTTGAGACAAGCATATGACTACTGGCAGGATCAACCAGGTCGAAGAGCACACGTCTGAACTCCAGTCACGGAAGGATATCTCGTATGCCGTCTTCTGCTTGAAAAAAAAAAAAAAAAGTCGC

>Contig30

GTGAAGAATTATGGACGTCCGGATGGTGTGCAGAAGCTTACAAACTAACTTCCTGGTGGAGAATAATGGTAGGCTTGGCGGCTCTGAGATGGAGATCGGGAAGAGCACACGTCTGAACTCCAGTCACGGAAGGATAGCTCGTATGCCGTCTTCTGCTTGAAAAAAAAAACAAAAAA

>Contig31

AACGGTTTAGTGTTTTCTTACCCAATTGTAGAGACTATCCACAAGGACAATATTTGTGACTTATGTTATGCGCCTGCTAGAGTTCCGGACTCCGTTCAACTTAAGGCGAAGTTGTTGAGATCGGAAGAGCACACGTCTGAACTCCAGTCACGGAAGGATATCTCGTATGCCGTCTTCTGCTTGAAAAAAAAAAAAAAAAA

>Contig32

GAGGAACTAAAAGTCGTAACAAGGTTTCCGTAGGTGAACCTGCGGAAGGATCATTAAAGAAATTTAATAATTTTGAAAATGGATTTTTTTGTTTTGGCAAGAGCATGAGAGCTTTTACTGGGCAAGAAGAGATCGGAAGAGACACGTCTGAACTCCAGTCACGGAAGGATATCTCGTATGCCGTCTTCTGCTTGAAAAAAAAAAAACATC

>Contig33

ACGGTTTAGTGTTTTCTTACCCAATTGTAGAGACTATCCACAAGGACAATATTTGTGACTTATGTTATGCGCCTGCTAGAGATCGGAAGAGCACACGTCTGAACTCCAGTCACGGAAGGATATCTCGTATGCCGTCTTCTGCTTGAAAAAAAAAAAAAAAAAAA

>Contig34

TAGGATGTATACGTAATACGTAATAGGTATCACGCTATTTACTCTTGCCTTTTCTTTGTTTCATAGTTCGAATCACATGAGTAGAGGACGGAAATTGCTAGATCGGAAGAGCACCACGTCTGAACTCCAGTCACGGAAGGATATCTCGTATGCCGTCTTCTGCTTGAAAAAAAAAAAAAAAA

>Contig35

TTAAATTGTCCATGAACATCACCGACTCTTCCGATTTTACCCCTATTAAATGTGCATGACACACCAGTGAAACCCCCATTGTGACTGGCCTAATAGGATGTATACGTAATACGTAATAGGTATCACGCTATTTACTCTTGCCTTTTCTTTGTTTCATAGTTCGAATCACATGAGTAGAGGACGGAAATTGCTCTTCTATGGCGTTCAGTATTCGAATAAATCCCAGACGCTAGGCTTATATAAATGAATGTAGCTGTACTTTACCAGCGTCTCAGGAATGAAGGCGTGCGTGTGGTCATAGTGGTCAATGAGATCAAATACAGATGCGAATGGA

>Contig36

GTGAAGAATTATGGACGTCCGGATGGTGTGCAGAAGCTTACAAACTAACTTCCTGGTGGAGAATAATGGTAGGCTTGGCGGCTCTGAGATGGAGAAATAGTTTCAAGCCGCGCGCTCAATAACTTCCTCGATCACTGACTTTCGCACCCATCTGGGAACGGTACGGCGAATAGATCGGAAGAGCACACGCCGAAATCCAGTCACGGAAGGATACCTCGTATGCC

>Contig37

GAGGAACTAAACGTCGTAACAAGGTTTCCGTAGGTGAACCTGCGGAA

>Contig38

ATAGAAAGTTCCATTAATCCGACATTAAAATTGGATGGATTAATGGAGAACACAAAGGCAAAAAAACAATGGAAGAACAAAGAAAATTTAGCGGAAGTAAAAATAACAGCCGAAAGCCAAATTCAGGCTTATCTTGCCTACTCTTTCTTTTATCGAATTCCTTTAGGCCGTTGCAATAGAAAAGTAATAAAAACGCATATACGTAAGTTGTAGTCAGTGTAATTGCAATCTATTATGCGCATC

>Contig39

GAGGAACTAAAAGTCGTAACAAGGTTTCAGTAGGTGAACCTGCGGAA

>Contig40

GAACCCTTATTCCCCGTTACCCGTTGAAACCATGGTAGGCCACTATCCTACCATCGAAAGTTGATAGGGCAGAAATTTGAATGAACCATCGCCAGCACAAGGCCATGCGATTCGAAAAGTTATTATGAATCATCAAAGAGTCCGAAGACATTGATTTTTTATCTAATAAATACATCTCTTCCAAAGGGTCGAGATTTTAAGCATGTATTAGCTCTAGAATTACCACAGTTATACCATGTAGTAAAGGAACTATCAAATAAACGATAACTGATTTAATGAGCCATTCGCAGTTTCACTGTATAAATTGCTTATACTTAGACATGCATGGCTTAATCTTTGAGACAAGCATATGACTACTGGCAGGATCAACCAGATAACTATCTTAAAAGAAGAAGCAACAAGCAGTAAAAAAGAAAGAAACCGAAATCTCTTTTTTTTTTTCCCACCTATTCCCTCTTGCTAGAAGATACTTATTGAGTTTGGAAACAGCTGAAATTCCAGAAAAATTGCTTTTTCAGGTCTCTCTGCTGCCGGAAATGCTCTCTGTTCA

**2CopyDiploid_1-12.contigs**

>Contig1

TCGTAACCTGCGCTTACAAAACTAACCACGTGCAGCATCGTCTTGTGTTTGTCCGTCCCTTGGGGTTCTTCGGATTTTCTATCTCCTCTTTTTTTTTCCTTACACTTTCATTTTGGGAAAAATTCGATTCTTTTTCTTTGATTTTTTTTTGGTTTCCTCTTGAATCTGTAAAGAACAAGAGCAGAAACTTAAAGTTAAGAAGGACAATAAAGAGCATACTATTACAAAATAATAGG

>Contig2

TACGCAAAAACGCTTTGAAAATTCTCCAAGAATCCTATTTGAAACTCTTATTAATAAATAAAGTATAAATATAATAAAGTTTATTGTGAGACATGTCGCGATAAGCACCCCTTGTCTTTCTTGGAAGGGAGAAATTTGTAATATAAAGCCAAGTGTTCAGAACTCGATTTTTTTCTGACCAAAGAGCGGAAGCTCCACTATAAAAGTTGGGAGGTACTTTTAGGTTCTCTTAAGTTC

>Contig3

GTAACGGCGAGTGAAGCGGCAAAAGCTCAAATTTGAAATCTGGTACCTTCGGTGCCCGAGTTGTAATTTGGAGAGGGCAACTTTGGGGCCGTTCCTTGTCTATGTTCCTTGGAACAGGACGTCATAGAGGGTGAGAATCCCGTGTGGCGAGGAGTGCGGTTCTTTGTAAAGTGCCTTCGAAGAGTCGAGTTGTTTGGGAATGCAGCTCTAAGTGGGTGGTAAATTCCATCTAAAGCTA

>Contig4

TCATTCGCTTTACCTCATAAAACTGATACGAGCTTCTGCTATCCTGAGGGAAACTTCGGCAGGAACCAGCTACTAGATGGTTCGATTAGTCTTTCGCCCCTATACCCAAATTCGACGATCGATTTGCACGTCAGAACCGCTACGAGCCTCCACCAGAGTTTCCTCTGGCTTCACCCTATTCAGGCATAGTTCACCATCTTTCGGGTCCCAACAGCTATGCTCTTACTCAAATCCATCC

>Contig5

TATAACAAAGAAGAAATAATGAACTGATTCTCTTCCTCCTTCTTGTCCTTTCTTAATTCTGTTGTAATTACCTTCCTTTGTAATTTTTTTTGTAATTATTCTTCTTAATAATCCAAACAAACACACATATTACAATAATGCCAAAGTTAGTTTTAGTTAGACACGGTCAATCCGAATGGAACGAAAAGAACTTATTCACCGGTTGGGTTGATGTTAAATTGTCTGCCAAGGGTCAAC

>Contig6

GTGGGTGGTAAATTCCATCTAAAGCTAAATATTGGCGAGAGACCGATAGCGAACAAGTACAGTGATGGAAAGATGAAAAGAACTTTGAAAAGAGAGTGAAAAAGTACGTGAAATTGTTGAAAGGGAAGGGCATTTGATCAGACATGGTGTTTTGTGCCCTCTGCTCCTTGTGGGTAGGGGAATCTCGCATTTCACTGGGCCAGCATCAGTTTTGGTGGCAGGATAAATCCATAGGAATGTAGCTT

>Contig7

GAACATTGTTCGCCTAGACGCTCTCTTCTTATCGATAACGTTCCAATACGCTCAGTATAAAAAAAGATTAGCCGCAGTTGGTAAAACCTAAAACGACCGTACTTGCATTATACCTCAAGCACGCAGAGAAACCTCTCTTTGGAAAAAAAACATCCAATGAAAAGGCCAGCAATTTCAAGTTAACTCCAAAGAGTATCACTCACTACCAAACAGAATGTTTGAGAAGGAAATGAC

>Contig8

ACGGTTTAGTGTTTTCTTACCCAATTGTAGAGACTATCCACAAGGACAATATTTGTGACTTATGTTATGCGCCTGCTAGAGTTCCGGACTCCGTTCAACTTAAGGCGAAGTTGTTGGCAGAAAATGCAATCAAAACAGATCGGAAGAGCACACGTCTGAACTCCAGTCACGGCGTTATATCTCGTATGCCGTCTTCTGCTTGAAAAAAAAAAAAAAACAA

>Contig9

AACGGTTTAGTGTTTTCTTACCCAATTGTAGAGACTATCCACAAGGACAATATTTGTGACTTATGTTATGCGCCTGCTAGAGTTCCGGACTCCGTTCAACTTAAGGCGAAGTTGTTGGCAGAAAATGCAATCAAATCTTTTCCCGGTTGTGGTATATTTGGTGTGGAAATGTTCTATTTAGAAACAGGGGAATTGCTTATTAACGAAATTGCCCCAAGGCCTCACAACTCTGGACATTATACCATTGA

>Contig10

ACGGTTTAGTGTTTTCTTACCCAATTGTAGAGACTATCCACAAGGACAATATTTGTGACTTATGTTATGCGCCTGCTAGAGTTCCGGACTCCGTTCAACTAGATCGGAAGAGCACGTCTGAACTCCAGTCACGGCGTTATATCTCGTATGCCGTCTTCTGCTTGAAAAAAAAAAAAGAACA

>Contig11

GAGGAAACTCAAAGAGTGCTATGGTATGGTGACGGAGTGCGCTGGTCAAGAGTGTAAAAGCTTTTTGAACAGAGAGCATTTCCGGCAGCAGAGAGACCTGAAAAAGCAATTTTTCTGGAATTTCAGCTGTTTCCAAACTCAATAAGTATCTTCTAGCAAGAGGGAATAGGTGGGAAAAAAAAAAAAAGATTTCG

>Contig12

AGTATCTTCTAGCAAGAGGGAATAGGTGGGAAAAAAAAAAAGAGATTTCGGTTTCTTTCTTTTTTACTGCTTGTTGCTTCTTCTTTTAAGATAGTTATCTGGTTGATCCTGCCAGTAGTCATATGCTTGTCTCAAAGATTAAGCCATGCATGTCTAAGTATAAGCAATTTATACAGTGAAACTGCGAATGGCTCATTAAATCAGTTATCGTTTATTTGATAGTTCCTTTACTACAT

>Contig13

CAGACCTCACAATCAGATCGGAAAGCACACGTCTGAACTCCAGTCACGGCGTTATATCTCGTATGCCGTCTTCTGCTTGAAAAAAAAAAAAACAACA

>Contig14

CTACTGCGAAAGCATTTGCCAAGGACGTTTTCATTAATCAAGAACGAAAGTTAGGGGATCGAAGATGATCAGATACCGTCGTAGTCTTAACCATAAACTATGCCGACTAGGGATCGGGTGGTGTTTTTTTAATGACCCACTCGGCACCTTACGAGAAATCAAAGTCTTTGGGTTCTGGGGGGAGTATGGTCGCAAGGCTGAAACTTAAAGGAATTGACGGAAGGGCAC

>Contig15

GAAAATGAGATAGATACATGCGTGGGTCAATTGCCTTGTGTCATCATTTACTCCAGGCAGGTTGCATCACTCCATTGAGGTTGTGCCCGTTTTTTGCCTGTTTGTGCCCCTGTTCTCAAGATCGGAAGAGCACAACGCCTGAACTCCAGTCACGGCGTTATATCTCGTATGCCGTCTTCTGCTTGAAAAAAAAAAAAACACA

>Contig16

ACGGTTTAGTGTTTTCTTACCCAATTGTAGAGACTATCCACAAGGACAATATTTGTGACTTATGTTATGCGCCTGCTAGAGTTCCGGACTCCGTTCAACTTAAGGCGAAGTTGTTGGCAGAAAATGCAATCAAATCTTTTCCCGGTTGTGGTATATTTGGTGTGGAAATGTTCTAAGATCGGAAGAGCACACGTCTGAACTCCAGTCACGGCGTTATATCTCGTATGCCGTCTTCTGCTTGAAAAAAA

>Contig17

ACGGTTTAGTGTTTTCTTACCCAATTGTAGAGACTATCCACAAGGACAAAGAGCGAAAAACACACGTCTGAACTCCAGTCACGGCGTTATATCTCGTATGCCGTCTTCTGCTTGAAAAAAAAAAACATAACAAA

>Contig18

ACGGTTTAGTGTTTTCTTACCCAATTGTAGAGACTATCCACAAGGACAATATTTGTGACTTATGTTATGCGCCTGCTAGAGTTCCGGACAGATCGGAAGAGCACACGTCTGAACTCCAGTCACGGCGTTATATCTCGTATGCCGTCTTCTGCTTGAAAAAAAAACAAA

>Contig19

ATCGTTTATTTGATAGTTCCTTTACTACATGGTATAACTGTGGTAATTCTAGAGCTAATACATGCTTAAAATCTCGACCCTTTGGAAGAGATGTATTTATTAGATAAAAAATCAATGTCTTCGGACTCTTTGATGATTCATAATAACTTTTCGAATCGCATGGCCTTGTGCTGGCGATGGTTCATTCAAATTTCTGCCCTATCAACTTTCGATGGTAGGATAGTGGCCTACCATGGTTTCA

>Contig20

ACGGTTTAGTGTTTTCTTACCCAATTGTAGAGACTATCCACAAGGACAATATTTGTGACTTATGTTATGCGCCTGCTAGAGTTCCGGACTCCGTTCAACTTAAGGCGAAGAGATCGGAAAGAGCACACGTCTGAACTCCAGTCACGGCGTTATATCTCGTATGCCGTCTTCTGCTTGAAAAAAAAAAAAAAAGTAA

>Contig21

ACGGTTTAGTGTTTTCTTACCCAATTGTAGAGACTATCCACAAGGACAATATTTGTGACTTATGTTATGCGCCAGATCGGAAGAGCACAGTCTGAACTCCAGTCACGGCGTTATATCTCGTATGCCGTCTTCTGCTTGAAAAAAAAAAACAAC

>Contig22

AGAAAATGAGATAGATACATGCGTGGGTCAATTGCCTTGTGTCATCATTTACTCCAGGCAGGTTGCATCACTCCATTGAGGTTGTGCCCGTTTTTTGCCTGTTTGTGCCCCTGTTCTCTGTAGTTGCGCTAAGAGAATGGACCTATGAACTGATGGTTGGTGAAGAAAACAATATTTTGGTGCTGGGATTCTTTTTTTTTCTGGATGCCAGCTTAAAAAGCGGGCTCCATTATATTTAGT

**2CopyDiploid_13-24.contigs**

>Contig1

GTCTTCAATAAATCCAAGAATTTCACCTCTGACAATTGAATACTGATGCCCCCGACCGTCCCTATTAATCATTACGATGGTCCTAGAAACCAACAAAATAGAACCAAACGTCCTATTCTATTATTCCATGCTAATATATTCGAGCAATACGCCTGCTTTGAACACTCTAATTTTTTCAAAGTAAAAGTCCTGGTTCGCCAAGAGCCACAAGGACTCAAGGTTAGCCAGAAGGAAAG

>Contig2

TGAATAATATTTCTTTTTCTGTGCCTTAACTGACCTGTCAACTAGAATTTTGCTTGTTCTTTCGTGATTACGAAACCCTATCTTCGTTTAAGTAAAATAGATAATTAATTTTTTACTCCAGAAAAGAAATAGAAAGTTTCTCTGTCTTTTCCTACGTCATAAAATTTTGATGATTCATTTTTGTATTACATCGGTAGAAAATTTTTCATACAGGGTAAAATTCTTTGTTTCTACAACT

>Contig3

AGGGAACAAAAGCTGGAGCTCTAGTACGGATTAGAAGCCGCCGAGCGGGTGACAGCCCTCCGAAGGAAGACTCTCCTCCGTGCGTCCTCGTCTTCACCGGTCGCGTTCCTGAAACGCAGATGTGCCTCGCGCCGCACTGCTCCGAACAATAAAGATTCTACAATACTAGCTTTTATGGTTATGAAGAGGAAAAATTGGCAGTAACCTGGCCCCACAAACCTTCAAATGAACGAATCAAAAAGAACG

>Contig4

GAGAACCTTTGGTCTTGGCTAATCTCAAAATTAAAGTTCATATCTACCGTATGAAGCGCTGATAAAGAGCGCCAAATCTGCACCTTGTGCATTTTGTTCACACATTCTTGGCGCTCCTAACTGGAGAAACGTCTATCATTGAGGATAACTCCGGATAGTCGCATTCTGATTGAATAGCCGTCTAGCTCCCTATTGTGACTAGAATACTATTGACATCGTTTTCTCATCAAAACCTCCC

>Contig5

ACGGTTTAGTGTTTTCTTACCCAATTGTAGAGACTATCCACAAGAGATCGGAAGAGGCACACGTCTGAACTCCAGTCACCTGTTGACATCTCGTATGCCGTCTTCTGCTTGAAAAAAAAAAAAAAA

>Contig6

AACGGTTTAGTGTTTTCTTACCCAATTGTAGAGACTATCCACAAGGACAATATTTGTGACTTATGTTATGCGCCTGCTAGAGTTCCGGACTCCGTTCAACTTAAGGCGAAGTTGTTGGCAGAAAATGCAATCAAATCTTTTCCCGGTTGTGGTATATTTGGTGTGGAAATGTTCTATTTAGAAACAGGGGAATTGCTTATTAACGAAATTGCCCCAAGGCCTCACAACTCTGGACATTATACCAT

>Contig7

GAAGAGAGCGTCTAGGCGAACAATGTTCTTAAAGTTTGACCTCAAATCAGGTAGGAGTACCCGCTGAACTTAAGCATATCAATAAGCGGAGGAAAAGAAACCAACCGGGATTGCCTTAGTAACGGCGAGTGAAGCGGCAAAAGCTCAAATTTGAAATCTGGTACCTTCGGTGCCCGAGTTGTAATTTGGAGAGGGCAACTTTGGGGCCGTTCCTTGTCTATGTTCCTTGGAACAGGACGT

>Contig8

TAAATACATCTCTTCCAAAGGGTCGAGATTTTAAGCATGTATTAGCTCTAGAATTACCACAGTTATACCATGTAGTAAAGGAACTATCAAATAAACGATAACTGATTTAATGAGCCATTCGCAGTTTCACTGTATAAATTGCTTATACTTAGACATGCATGGCTTAATCTTTGAGACAAGCATATGACTACTGGCAGGATCAACCAGATAACTATCTTAAAAGAAGAAGCAACAAGCAAA

>Contig9

CAAAGAAAGGCTAAGTCATACAATAGGCAAAAGACCTTTGTCTTTCCTTTTTTTCCTCTTTCCTAACATACGGTGGTTTATGTACTATCCCTTATGTAATTGCTTAGCTATACACAGTGAGCACACATAGTGGCGCTCACTACTCCCTTTTAATTTCAACTCGTAGATAGTTGGGCTATTTTCCTTCGAATCCTCGTTTATCATCTCATATTTAAGAGTACCATTTCGGGGCTTTTTTT

>Contig10

ACGGTTTAGTGTTTTCTTACCCAATTGTAGAGACTATCCACAAGGACAATATTTGTGACTTATGTTATGCGCCTGCTAGAGTTCCGGACTCCGTTCAACTTAAGGCGAAGTTGTTGGCAGAAAATGCAATCAAATCTTTTCCCGAGATCGGAAAGAGCACACGTCTGAACTCCAGTCACCTGTTGACATCTCGTATGCCGTCTTCTGCTTGAAAAAAAAAAAAAAAAAA

>Contig11

ACGGTTTAGTGTTTTCTTACCCAATTGTAGAGACTATCCACAAGGACAATATTTGTGACTAGATCGGAAGAGCACACGTCTGAATCCAGTCACCTGTTGACATCTCGTATGCCGTCTTCTGCTTGAAAAAAAAAAAAAAAAAA

>Contig12

ATGACCAAGTTTGTCCAAATTCTCCGCTCTGAGATGGAGTTGCCCCCTTCTCTAAGCAGATCCTGAGGCCTCACTAAGCCATTCAATCGGTACTAGCGACAGATCGGAAGAGCAACACGTCTGAACTCCAGTCACCTGTTGACATCTCGTATGCCGTCTTCTGCTTGAAAAAAAAAAAAAATACAGATAG

>Contig13

GAAGTCATTCGAATTAAAATTCAATTTCCGATACTCCCCATCGAGAATAGACTCACATAGAGTC

>Contig14

ACGGTTTAGTGTTTTCTTACCCAATTGTAGAGACTATCCACAAGGACAATATTTGTGACTTATGTTATGCGCCTGCTAGATCGGAAGAGCACACGTCTGAACTCCCAGTCACCTGTTGACATCTCGTATGCCGTCTTCTGCTTGAAAAAAAAAAAAA

>Contig15

AGAGATGGGGAAGCTCCGTTTCAAAGGCCTGATTTTATGCAGGCCACCATCGAAAGGGAATCCGGTTAAGATTCCGGAACCTGGATATGGATTCTTCACGGTAACGTCGATCGGAGAAAC

>Contig16

ACGAAAATTCTTGTTTTTGACAAAAATTTAATGAATAGATAAAATTGTTTGTGTTTGTTACCTCTGGGCCCCGATTGCTCGAATGCCCAAAGAAAAAGTTGCAAAGATATGAAAACTCCACAGTGTGTTGTATTGAAACGGTTTTAATTGTCCTATAACAAAAGCACAGAAATCTCTCACCGTTTGGAATAGCAAGAAAGAAACTTACAAGCCTAGCAAGACCGCGCACTTAAGCGCAGGCCCGGCTGGACTCTCCATCTCTTGTCTTCTTGCCCAGTAAAAGCTCTCATGCTCTTGCCAAAACAAAAAAATCCATTTTCAAAATTATTAAATTTCTTT

>Contig17

ACGGTTTAGTGTTTTCTTACCCAATTGTAGAGATCGGAGAGCACACGTCTGAACTCCAGTCACCTGTTGACATCTCGTATGCCGTCTTCTGCTTGAAAAAAAAAAAAAAAA

>Contig18

AGAACATTGTTCGCCTAGACGCTCTCTTCTTATCGATAACGTTCCAATACGCTCAGTATAAAAAAAGATTAGCCGCAGTTGGTAAAACCTAAAACGACCGTACTTGCATTATACCTCAAGCACGCAGAGAAACCTCTCTTTGGAAAAAAAACATCCAATGAAAAGGCCAGCAATTTCAAGTTAACTCCAAAGAGTATCACTCACTACCAAACAGAATATTTGAGAAAGAAATAAC

**2CopyDiploid_25-36.contigs**

>Contig1

TGCTGTACAACTCATGTAACCCCCACTATCTCAGGAGTTATATTAAATCAAGGCACGCCAGTGCTGCCTCTCTTGGTACGACAAACAAGTTTTTCCCGATTCTAGGTATTGCATTACATAATATCAACCCTTTGTTGTTTTCTTTTGATTTTTGAAAAGAAATATCTCATACCAACCGGAAAGGAAAAAGGACACAATTTGGGAAAATAGGCAATTTATTATGTACACAAGTCAAAA

>Contig2

GAGAATCGAAAAATAACCCCCAGCTGGGATGTGATCACGATGAGTGACGGGATACATTAAATATAACAAACATACACGTTGCCTTGAACGTTTTCACTACACATCGCATGTGTTTTATCTAGATTTAAAATCATATTACTACAGTTCTACAGTTATACAAATAACAAACAATAGAAAAATGTTTAGACAGTGTGCTAAGAGATATGCATCTTCATTACCCCCAAATGCTTTGAAACC

>Contig3

AACGGTTTAGTGTTTTCTTACCCAATTGTAGAGACTATCCACAAGGACAATATTTGTGACTTATGTTATGCGCCTGCTAGAGTTCCGGACTCCGTTCAACTTAAGGCGAAGTTGTTGGCAGAAAATGCAATCAAATCTTTTCCCGGTTGTGGTATATTTGGTGTGGAAATGTTCTATTTAGAAACAGGGGAATTGCTTATTAACGAAATTGCCCCAAGGCCTCACAACTCTGGACATTAT

>Contig4

GTATTTCTTTTGCTCCTCCTATCCAAGAAGGGAAAAATGCTTGGCGACTAGATATATGGTCTGATTTCGTTTCTTTTTCTAGTTGAAAGAAAAGGAAGAAAGGAATGACGGCAGAAAAAGATCTGAGAAAAGTGCGTATATTGAAGCTATACGGGGTATAAAACTTGATATACACGGTAAAAGAAAATACACTGATAACCATGATCATTTTCCAATTTAAGAATATTAAGGAGTAATTT

>Contig5

AAAAAACACCACCCGATCCCTAGTCGGCATAGTTTATGGTTAAGACTACGACGGTATCTGATCATCTTCGATCCCCTAACTTTCGTTCTTGATTAATGAAAACGTCCTTGGCAAATGCTTTCGCAGTAGTTAGTCTTCAATAAATCCAAGAATTTCACCTCTGACAATTGCATACTGATGCACCCGACCGTCCCTATTAATCATTACGATG

>Contig6

AATCCATTCATGCGCGTCACTAATTAGATGACGAGGCATTTGGCTACCTTAAGAGAGTCATAGTTACTCCCGCCGTTTACCCGCGCTTGGTTGAATTTCTTCACTTTGACATTCAGAGCACTGGGCAGAAATCACATTGCGTCAACATCACTTTCTGACCATCGCAATGCTATGTTTTAATTAGACAGTCAGATTCCCCTTGTCCGTACCAGTTCTAAGTTGATCGTTAATTGTAGCA

>Contig7

TTTGTTTTTTATTTTTTTTTTTGCGCGAAACTTTGCTATATTGGGTAACGCGTAAAATACTTTTTATTATTGCAGTAAGGCGGAAGGGTCTTCCCCTTTGCATGTTAAATAGCATACATGGCACCACTCAGGTCCAGAACGTGACACATCTTTGCACCACTTGTGTATTTTCAAGATGTTAAATTTTTGATACATAAGCTTTATCATGCAGGTGCAGTAGACGCATTTGCGTACGCGTT

>Contig8

ATGACCAAGTTTGTCCAAATTCTCCGCTCTGAGATGGAGTTGCCCCCTTCTCTAAGCAGATCCTGAGGCCTCACTAAGCCATTCAATCGGTACTAGCGACGGGCGGTGTGTACAAAGGGCAGGGACGTAATCAACGCAAGCTGATGACTTGCGCTTACTAGGAATTCCTCGTTGAAGAGCAATAATTACAATGCTCTATCCCCAGCACGACGGAGTTTCACAAGATTACCAAGA

>Contig9

ACGGTTTAGTGTTTTCTTACCCAATTGTAGAGACTATCCACAAGGACAATATTTGTGACTTATGTTATGCGCCTGCTAGAGAGATCGGAAGAGCACACGTCTGAACTCAGTCACGTCATCGAATCTCGTATGCCGTCTTCTGCTTGAAAAAAAAAAAAAAA

>Contig10

GAAAGAAATAAAAAACAAATCAGACAACAAAGGCTTAATCTCAGCAGATCGTAACAACAAGAGATCGGAAGAGCAAAGTCTGAACTCCAGTCACGTCATCGAATCTCGTATGCCGTCTTCTGCTTGAAAAAAAAAAAACAACAAA

>Contig11

AGAAAGAAATAAAAAACAAATCAGACAACAAAGGCTTAATCTCAGCAGATCGTAACAACAAGGCTACTCTACTGCTTACAATACCCCGTTGTACATCTAAGTCGTATACAAATGATTTATCCCCACGCAAAATGACATTGCAATTCGCCAGCAAGCACCCAAGGCCTTTCCGCCAAGTGCACCGTTGCTAGCCTGCTATGGTTCAGCGACGCCACAAGGACGCCTTATTCGTATCCATCT

>Contig12

ACGGTTTAGTGTTTTCTTACCCAATTGTAGAGACTATCCACAAGGAGATCGGAAGAGCACACGTCTGAACTCAGTCACGTCATCGAATCTCGTATGCCGTCTTCTGCTTGAAAAAAAAAAACACATAA

>Contig13

AATATTCACTGGCACTCAAGTTATGCTGATCTTGGAGCGATCTTGAAGCCGAGTATGTCATTCTTACCGGGCTCAACAAGACTTTCTTCACTGACTCTCACGCCGCCAACGGTTTCAATGCTTGCAATGGTGACCTCGCCACCCATGATATACCTGGCAACTTTGCCAACAGGGGTTCCTCGCTTGACCAAAAAACAATCTCTAAATACGTTGGTTCCGTTGCCACCCGTAAAAGTCTG

>Contig14

AAGCTCTTCATTCAAATGTCCACGTTCAATTAAGTAACAAGGACTTCTTACAAGATCGGAAGAAGCACACGTCTGAACTCCAGTCACGTCATCGAATCTCGTATGCCGTCTTCTGCTTGAAAAAAAAAAAAAAAAAAA

>Contig15

ACGGTTTAGTGTTTTCTTACCCAATTGTAGAGACTATCCACAAGGACAATATTTGTGACTTATGTTATGCGCCTGCTAGAGTTCCGGACTCCGTTCAACTTAAGGCGAAGTTGTTGAGATCGGGAAGAGCACACGTCTGAACTCCAGTCACGTCATCGAATCTCGTATGCCGTCTTCTGCTTAAAAAAAAAAAAAAAA

>Contig16

AGGTCACTTCTTCGTTTCCTTTATTGGGGTTTCCGTGTAGCCTTCCCCTGAATAGTGTGGGACGTTTTATGAGAAGCCGTAAGAAATAGGCAAATTGAGTTATGACAAGTAGACATGATGCCGCAGCCTTGCCTGACTTTACGTCTCCTTCATGAATAAGTTTTTCTATCGAGTTCTTTTCCTTTTTTCGCCTTAATTAGCTCAATTAAGCCTGTCCTCACTACTTTTCTTTTTCTTAT

>Contig17

AACGGTTTAGTGTTTTCTTACCCAATTGTAGAGACTATCCACAAGGACAATATTTGTGACTTATGTTATGCGCCTGCTAGAGTTCCGGACTCCGTTCAACTTAAGGCGAAGTTGTTGGCAGAAAATGCAATCAAATCTTTTCCCGGAGATCAAGAGCACACGTCTGAACTCCAGTCACGTCATCGAATCTCGTATGCCGTCTTCTGCTTGAAAAAAAAAAAAAAAAAACA

>Contig18

CCTAAAAGCTCTTCATTCAAATGTCCACGTTCAATTAAGTAACAAGGACTTCTTACATATTTAAAGTTTGAGAATAGGTCAAGGTCATTTCGACCCCGGAACCTCTAATCATTCGCTTTACCTCATAAAACTGATACGAGCTTCTGCTATCCTGAGGGAAACTTCGGCAGGAACCAGCTACTAGATGGTTCGATTAGTCTTTCGCCCCTATACCCAAATTCGACGATCGATTTGCACGTC

>Contig19

AAGCTCTTCATTCAAATGTCCACGTTCAATTAAGTAACAAGGACTTCTTACATATTTAAAGTTTGAGAATAGAGATCGGAAGAGCACACGTCTGAACTCCATCACGTCATCGAATCTCGTATGCCGTCTTCTGCTTGAAAAAAAAAACAAATAGC

**2CopyDiploid_37-48.contigs**

>Contig1

TATAGCAAAAAAGTGCTCGTGAATGTCTCTCTGAAAATGCTGGAAACTGATCACTCTAGGAATGACAATTTAGACGATAAAAGCACTGTCTGCTACAGCGAAAAGACAGATAGCAATGTTGAGAAGTCTACCACGTCTGGCTTGCGCCGTATAGACGCTGTCAACAAGGTCTTGTCTGACTACAGCTCTTTCACAGCTTTTGGAGTTACGTTTAGTTCACTCAAGACCGCCCTACTGG

>Contig2

CGAACGAGACCTTAACCTACTAAATAGTGGTGCTAGCATTTGCTGGTTATCCACTTCTTAGAGGGACTATCGGTTTCAAGCCGATGGAAGTTTGAGGCAATAACAGGTCTGTGATGCCCTTAGACGTTCTGGGCCGCACGCGCGCTACACTGACGGAGCCAGCGAGTCTAACCTTGGCCGAGAGGTCTTGGTAATCTTGTGAAACTCCGTCGTGCTGGGGATAGAGCATTGTAATTATTGC

>Contig3

ACGGTTTAGTGTTTTCTTACCCAATTGTAGAGACTATCCACAAGGACAATATTTGTGACTTATGTTATGCGCCTGCTAGAGTTCCGGACTCCGTTCAACTTAAGGCGAAGAGATCGGAAGAGCACACGTCTGAACTCCAGTCACTGACTTCGACCTCGTATGCCGTCTTCTGCTTGAAAAAAAAAAA

>Contig4

GTATTTCTTTTGCTCCTCCTATCCAAGAAGGGAAAAATGCTTGGCGACTAGATATATGGTCTGATTTCGTTTCTTTTTCTAGTTGAAAGAAAAGGAAGAAAGGAATGACGGCAGAAAAAGATCTGAGAAAAGTGCGTATATT

>Contig5

ATTCTACACCCTCTATGTCTCTTCACAATGTCAAACTAGAGTCAAGCTCAACAGGGTCTTCTTTCCCCGCTGATTCTGCCAAGCCCGTTCCCTTGGCTGTGGTTTCGCTAGATAGTAGATAGGGACAGTGGGAATCTCGTTAATCCATTCATGCGCGTCACTAATTAGATGACGAGGCATTTGGCTACCTTAAGAGAGTCATAGTTACTCCCGCCGTTTACCCGCGCTTGGTTGAATTT

>Contig6

TGCCAGCATCCTTGACTTACGTCGCAGTCCTCAGTCCCAGCTGGCAGTATTCCCACAGGCTATAATACTTACCGAGGCAAGCTACATTCCTATGGATTTATCCTGCCACCAAAACTGATGCTGGCCCAGTGAAATGCGAGATTCCCCTACCCACAAGGAGCAGAGGGCACAAAACACCATGTCTGATCAAATGCCCTTCCCTTTCAACAATTTCACGTACTTT

>Contig7

ATTTCTGGCCTAAACAAGGTAGTGCTACCCTAGAGGAAGTACGATATCAGTACACTTTTGTATTTTTAACGAATTATGGTATTCTTTAAAATATTTAACATGCTATCGAGTTTTTCTTTCTAAAATGCGGTTTCAAAATTTGAGTCCGCTTTCTATGGAAAATATGAATGAGGCGAACGTGCTT

>Contig8

GAAAACTTTAGTCCTTGATGAAAGAAAATTTTCGTTTTTTTTTTCAGGTTCAGCGGGCTTAACCTCGTTTGCCGCCTCGGCCCCGCTCCGTTGTGCTGGCCATCTGTGCGCGGCGGGCTTCCCC

>Contig9

ACGGTTTAGTGTTTTCTTACCCAATTGTAGAGACTATCCACAAGGACAATATTTGTGACTTATGTTATGCGCCTGCTAGAGAGATCGGAAGAAGCACACGTCTGAACTCCAGTCACTGACTTCGATCTCGTATGCCGTCTTCTGCTTGAAAAAAAAAAAAAACAAT

>Contig10

GTCAATTTTACCTCTAGATCATATTAACTAATCTCATCTTGCACAAAAAACGTATCAC

>Contig11

TGCTAATAAACAATTTGATGATAATTCGTACCCTTTGACTCACCTCGAATATATATAGATATATATATACATATAACGTATATTTATATATATACGGTTTTGTTCGATTGTCTCTGTCGTACCATCAAAGAACATACTAACAGTTCACAATGTTAAGAATCAGATCACTCCTAAATAATAAGCGTGCCTTTTCGTCCACAGTCAGGACATTGACCATTAACAAGTCACATGATGTA

>Contig12

CCCCTATTAAATGTGCATGACACACCAGTGAAACCCCCATTGTGACTGGCCTTAACGGTTTAGTGTTTTCTTACCCAATTGTAGAGACTATCCACAAGGACAATATTTGTGACTTATGTTATGCGCCTGCTAGAGTTCCGGACTCCGTTCAACTTAAGGCGAAGTTGTTGGCAGAAAATGCAATCAAATCTTTTCCCGGTTGTGGTATATTTGGTGTGGAAATGTTCTATTTAGAAACAGGGGAATTGCTTATTAACGAAATTGCCCCAAGGCCTCACAACTCTGGACATTATACC

>Contig13

AATAACGATACAGGGCCCATTCGGGTCTTGTAATTGGAATGAGTACAATGTAAATACCTTAACGAGGAACAATTGGAGGGCAAGTCTGGTGCCAGCAGCCGCGGTAATTCCAGCTCCAATAGCGTATATTAAAGTTGTTGCAGTTAAAAAGCTCGTAGTTGAACTTTGGGCCCGGTTGGCCGGTCCGATTTTTTCGTGTACTGGATTTCCAACGGGGCCTTTCCTTCTGGCTAACCTTGAGTCCTT

>Contig14

ACGGTTTAGTGTTTTCTTACCCAATTGTAGAGACTATCCACAAGGACAATATTTGTGACTTATGTTATGCGCCTGCTAGATCGGAAGAGCACACGTCTGAACTCCAGTCACTGACTTCGATCTCGTATGCCGTCTTCTGCTTGAAAAAAAAAAAAAAAATAA

>Contig15

TAACTTTTCGAATCGCATGGCCTTGTACCCGCTGAAGCACGCAGCAACAACCGGTTGACCGACGCAATTCTTTGTTTACTTTTCCTTCTACTCATGATCTTCTGTTCCATTTGCCAC

>Contig16

TAAGAAGAGAGCGTCTAGGCGAACAATGTTCTTAAAGTTTGACCTCAAATCAGGTAGGAGTACCCGCTGAACTTAAGCATATCAATAAGCGGAGGAAAAGAAACCAACCGGGATTGCCTTAGTAACGGCGAGTGAAGCGGCAAAAGCTCAAATTTGAAATCTGGTACCTTCGGTGCCCGAGTTGTAATTTGGAGAGGGCAACTTTGGGGCCGTTCCTTGTCTATGTTCCTTGGAACAGGAC

>Contig17

ACGGTTTAGTGTTTTCTTACCCAATTGAGATCGGAAGAGCACACTCTGAACTCCAGTCACTGACTTCGATCTCGTATGCCGTCTTCTGCTTGAAAAAAAAAAAAAAATATCAAA

>Contig18

ACGGTTTAGTGTTTTCTTACCCAATTGTAGAGACTATCCACAAGGACAATATTTGTGACTTATGTTATGCGCCTGCTAGAGTTCCGGACTCCGTTCAACTTAAGGCGAAGTTGTTGGCAGAAAATGCAATCAAAAGATCGGAAAGAGCACACGTCTGAACTCCAGTCACTGACTTCGATCTCGTATGCCGTCTTCTGCTTGAAAAAAAAAAAAAAAACAAAA

>Contig19

ATAAAAACAATAACAACAGCAACAATAGCACAAGAACATTAACCCAAGCAACTAGCAATAACAAGAACGGTAATTATCAAAACAAAATATGATACCAAATGCGGAAAAAATCGGCACATATCCATAAAACATACACATCGTGCTTAGCGATCTACATACCGTGGAGGAATGGGTGGAACGGTTACTGACGACGGCCGCGCGGGGTTTTGTGTTGGGTGTGATGTCCGGATGCAACAGC

>Contig20

AATAACTTTTCGAATCGCATGGCCTTGTGCTGGCGATGGTTCATTCAAATTTCTGCCCTATCAACTTTCGATGGTAGGATAGTGGCCTACCATGGTTTCAACGGGTAACGGGGAATAAGGGTTCGATTCCGGAGAGGGAGCCTGAGAAACGGCTACCACATCCAAGGAAGGCAGCAGGCGCGCAAATTACCCAATCCTAATTCAGGGAGGTAGTGACAATAAATAACGATACAGGGCCC

>Contig21

TGAATCATCAAAGAGTCCGAAGACATTGATTTTTTATCTAATAAATACATCTCTTCCAAAGGGTCGAGATTTTAAGCATGTATTAGCTCTAGAATTACCACAGTTATACCATG

>Contig22

ACGGTTTAGTGTTTTCTTACCCAATTGTAGAGACTATCCACAAGGACAATATTTGTGACTTATGTTATGCACCTGCTAGAGTTCCGGACTCCGTTCAACTTAAGGCGAAGTTGTTGGCAGAAAATGCAATCAAATCTTTTCCCGAAAACCGAAAGAGCACACGTCTGAACTCCCAGTCACTGACTTCGATCTCGTATGCCGTCTTCTGCTTGAAAAAAAAAAAAAAA

>Contig23

ACGGTTTAGTGTTTTCTTACCCAATTGTAGAGACTATCCACAAGGACAATATTTGTGACTTATGTTATGCGCCTGCTAGAGTTCCGGACTCCGTTCAACTTAAGGCGAAGTTGTTGGCAGAAAATGCAATCAAATCTTTTCCCGGTTGTGGTATATTTGGTGTGGAAATAGATCGAAAAAGCACACGTCTGAACTCCAGTCACTGACTTCGAGCTCGTATGCAGTCTTCTGCTTGAAAA
